# Diagnostic evaluation of institutions as a basis for designing the Brazilian maturity model of telehealth services

**DOI:** 10.1186/s12913-024-10723-8

**Published:** 2024-03-25

**Authors:** Angélica Baptista Silva, Ivan Torres Pisa, Luiz Ary Messina, Andréa Pereira Simões Pelogi, Josceli Maria Tenório, Fernando Sequeira Sousa, Daniela Lacerda Santos, Jessi Maia, Ianê Germano de Andrade Filha, Ana Cristina Carneiro Menezes Guedes, Paulo Roberto de Lima Lopes, Paulo Ricardo da Silva Maia

**Affiliations:** 1 Oswaldo Cruz Foundation, Rio de Janeiro, Brazil; 2 Federal University of São Paulo, São Paulo, Brazil; 3 Brazilian National Research and Education Network - Distrito Federal, Rio de Janeiro, Brazil; 4 Centro Universitário Arthur Sá Earp Neto, Petrópolis, Brazil; 5 Federal University of the State of Rio de Janeiro, Rio de Janeiro, Brazil

**Keywords:** Telemedicine, Telehealth, Digital Health

## Abstract

**Background:**

The number and specificities of telehealth service units that expanded their services and diversified with the COVID-19 pandemic in Brazil need to be discovered. The objective of this manuscript is to present a methodology for the diagnostic evaluation of 19 telehealth units from different regions of the country for federal governmental decision-making.

**Methods:**

A cross-sectional quantitative and qualitative study was carried out in the form of a census based on administrative records with an online survey and in-depth interviews with local telehealth managers.

**Results:**

Despite the discontinuity of regular funding, the results point to a diversity of initiatives and advances. Citizenship, sustainability, security, and budget management are recurring themes in the maturity analysis of telehealth services after the advent of the pandemic.

**Conclusion:**

It is necessary for Brazil to build a resilient model of the maturity of telehealth services that contemplates the different regional scenarios.

**Supplementary Information:**

The online version contains supplementary material available at 10.1186/s12913-024-10723-8.

## Background

Modalities of telehealth emerged during the COVID-19 pandemic [1]. It has highlighted health inequalities, especially in low-income countries [2, 3]. The need for social isolation relied on the creativity of the care teams and managers of health facilities to quickly transpose face-to-face practices to remote ones [4]. Furthermore, global health faces a lack of definition of telehealth services, sometimes presented as digital health activities or e-health services [5, 6].

In Brazil, there are telehealth service units (TSUs) with 30 years of public incentives [7]. In the 1970s-80s, states and municipalities collected vital statistics data, which were computerized to encourage the decentralization of health actions [8]. Before the Unified Health System (SUS) creation in 1988, studies focused on telemedicine and the computerization of health care [9]. By the 1990s, institutional telehealth already existed, which gained momentum in the mid-2000s with federal funding; however such actions still need to be integrated into territorial healthcare networks [10].

Medical societies allowed teleconsultation only in Brazil in 2022, unlike in other countries in South America [11]. Teleconsultation became both strategic and recognized by law for maintaining care in traditionally face-to-face processes. Covid-19 virus’ spreading proved that traditional policy-making models did not adequately explain the reality of government decision-making as Kingdon argued years ago [12]. Decisions don’t seem to follow a strictly logical progression through government, and some issues seem to become ‘hot’ suddenly, with big changes implemented, like the Brazilian law about telehealth [13]. However, evidence-based guidelines are still being established, especially in Low and Middle-Income Countries and conflict territories [14]. Methods for evaluating these initiatives need to be implemented considering the related history and the emerging postpandemic actions [15]. TSUs are part of a face-to-face care institution’s structure, while others specialized in distance care, such as virtual clinics [16]. After the pandemic abated, an assessment of these care units became necessary so that they may identify with each other, advance their services, and integrate healthcare. Therefore, this investigation opted for diagnostic evaluation to blish the amplitude, nature, and implications of the factors that cause difficulties in deciding to improve a situation [17, 18].

The manuscript’s objective is to describe the methodological steps and discuss the results of a TSUs diagnostic evaluation conducted throughout 2022 to improve governmental decision-making.

## Methods

A cross-sectional study was designed as a bottom-up method’s census based on administrative records [19, 20]. The study included 19 TSUs, as the Ministry of Health (MoH) indicated. The units are located in 13 states within Brazil, with 186 million potential direct beneficiaries [21].

**Fig. 1:**
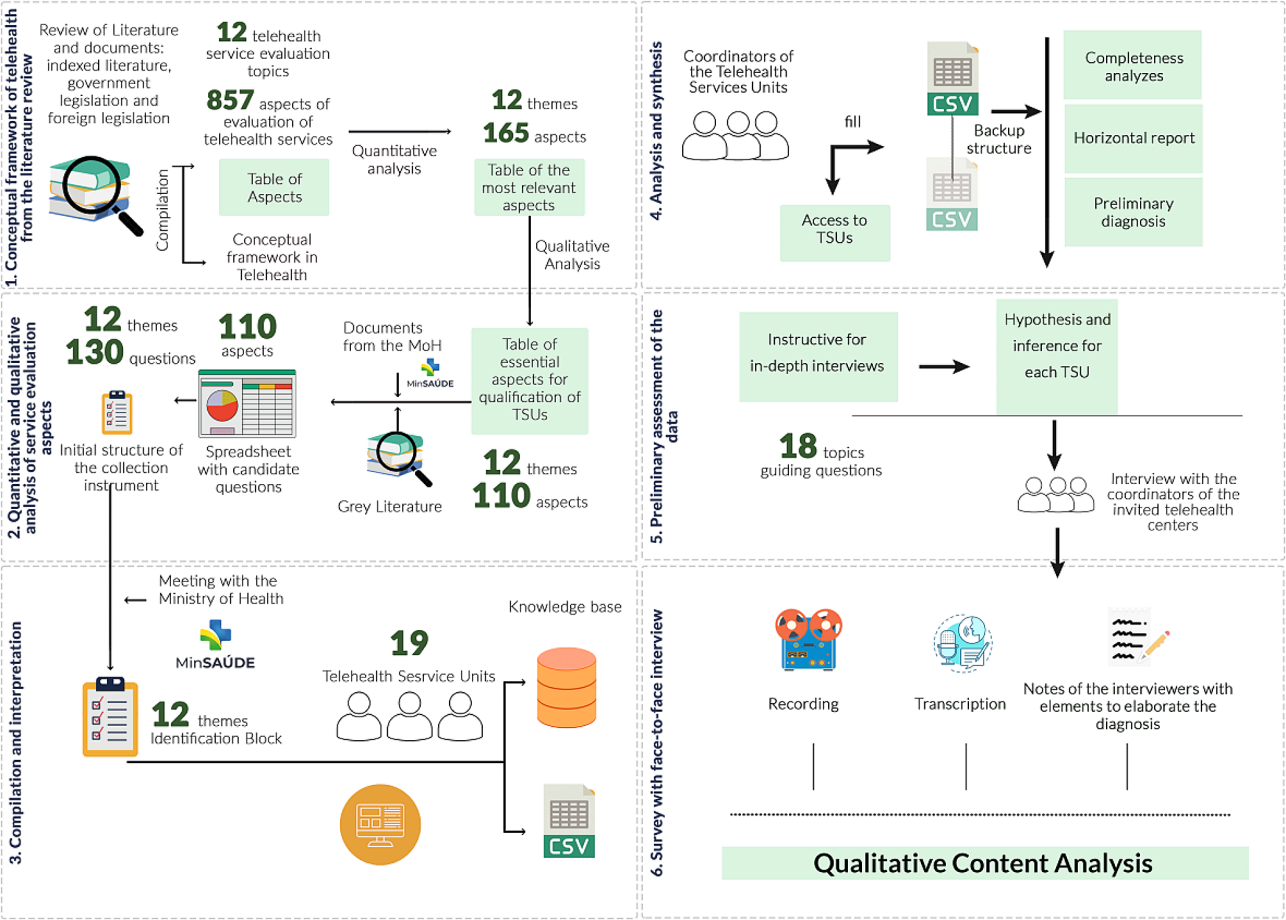
Synthesis of the method used to collect and analyse TSU data

The six steps of diagnostic evaluation were: a conceptual framework of telehealth and literature review; quantitative and qualitative analysis of service evaluation aspects; compilation and interpretation; analysis and synthesis; preliminary assessment of the data; and survey with face-to-face interviews (Fig. 1).

The framework was conceived after a review based on a published and peer-reviewed protocol [22, 23]. The team listed 12 research issues and 839 aspects of the evaluation of services to support a conceptual framework of maturity in telehealth. An aspect was a definition/field that indicates the essential of a service’s indicator or quality characteristic [24]. Concerning the survey, we presented an arrangement supported by ordinances, standards, and regulations at the federal level. We considered the concepts drawn from them regarding maturity models of digital health in the dimensions of health services. With this first step, we sought to ensure a robust instrument following the current scientific evidence. Comparisons were made between literature, technical documentation, and regulations.

The analysis [25] from the second stage supported the macro themes, namely, structure and management; financial and budget management; processes and activities; human resources; training and outreach; infrastructure and technology; monitoring and evaluation; acceptability and suitability; protection and security; legal and ethical aspects; innovation and research networks; and citizenship and sustainable development. We listed them with related aspects (see Supplementary data 1), mined the aspects and checked congruences and overlaps. Meetings with the MoH were held to select the most relevant aspects. Finally, 110 aspects were chosen for composing an online survey with 130 questions, 20 related to the description of the consulted TSUs (see Supplementary data 2).

In the third stage, we close the questions to reduce participants’ time. MoH validated the collection instrument, which opened an informed consent, an identification block and 12 separate blocks (Table 1).

**Table 1:** List of 12 themes with 34 topics adopted in the telehealth unit services assessment model

Themes	#	Related topics
1. Structure and management	1 2 3 4	1.1 Services offered by the TSU 1.2 Regulation and referrals 1.3 Organization chart and career 1.4 Topics of the National Health Plan
2. Financial and budget management	5 6 7	2.1 Financial plan with host institution* 2.2 Direct TSU costs 2.3 Economic result
3. Processes and activities	8 9 10	3.1 Flowchart and clinical protocols 3.2 Patient consent 3.3 Failures and Incidents
4. Human resources	11 12 13	4.1 Composition of the TSU team 4.2 TSU team training 4.3 TSU team qualification
5. Training and outreach	14 15	5.1 Continuing education of TSU staff 5.2 Training of requesting professionals
6. Infrastructure and technology	16 17 18 19 20	6.1 Physical structure 6.2 Telehealth electronic platform 6.3 Use of electronic registration systems 6.4 Technological structure of the storage 6.5 Technical support
7. Monitoring and evaluation	21 22 23 24	7.1 Activities monitoring strategies 7.2 Activities accounting categories 7.3 Satisfaction survey 7.4 Identification of difficulties and barriers
8. Acceptability and suitability	25	8.1 Leadership Engagement
9. Protection and security	26 27	9.1 Patient consent 9.2 Electronic Security
10. Legal and ethical aspects	28	10.1 Health information systems
11. Innovation and research networks	29 30 31	11.1 Education and research 11.2 Connection to National Health Data Network 11.3 Health Surveillance
12. Citizenship and sustainable development	32 33 34	11.2 Sustainability 12.2 Sources of funding 12.3 Citizenship

We created an account in the application *©WhatsApp Version 2.2245.9* on which the researchers took turns answering questions from the managers/respondents. During the month of completion, the fourth stage consisted of analysing the completeness and consistency of the questionnaire. We elaborated a horizontal report of the 19 TSUs, which was the basis for a preliminary diagnosis of the responding units.

A script for in-depth, face-to-face interviews was built based on the diagnosis and the gaps left in filling out the survey (see Supplementary data 3). We recorded the interviews via software *©Microsoft Teams Version 1.5.00.31168 (64 bits)* and performed a qualitative analysis of interview transcriptions.

Regarding the ethical aspects, the interviewees provided informed consent, and the data were aggregated, anonymized, and made available for public and unrestricted access (see Supplementary data 4) [26].

## Results

We present the TSUs diagnostic evaluation, and the global diagnosis on their services maturity. Sixteen managers agreed to participate in the interviews complementary to the online survey. Their profile was female (75%), with an average age of fifty-three, ranging between 29 and 71 years. Regarding the professional training of leaders, medicine predominated (5), followed by nursing (3); computer science and dentistry (both with 2); and administration, biology, biomedicine, and psychology (all with 1).

### TSU diagnosis by theme

The survey mapped the activities according to the theme *structure, and management*. TSUs offer teleeducation (16/17), asynchronous inter-consultation (14/17), and telediagnosis (12/17), via specialists integrated into the National Telediagnosis Platform. Teleconsultation (10/17) had the lowest number of offers. Health promotion activities are offered as support to the lines of care (13/16). Most TSUs are integrated with referrals to social assistance support and support epidemiological surveillance (9/16). They (13/16) are formally included in an institution’s organizational structure and have an organization chart for telehealth (9/16). The services cover primary, chronic disease, outpatient, and mental health care. Attention to indigenous health, and people with disabilities care is provided in three units.

According the second theme, *financial and budget management*, 11/17 TSUs have a financial plan. On average, 53% of TSUs direct costs are allocated to salaries or remuneration per activity of the permanent team. Other costs include fellowships (20%), physical facilities (10%), maintenance of services (7%), and services (9%). Monitoring and evaluation using financial situation indicators are conducted (8/13). Cost-minimization (4/13) and utility cost (3/13) indicators are used.

For *processes and activities*, units had established flows, protocols, and clinical guidelines, even in the form of good practices (6/15). Regarding the patient consent, TSUs (16) receive consent forms electronically through electronic registration platforms for teleconsultation (8/16), telediagnosis (6/16), and asynchronous (4/6) and synchronous (3/10) inter-consultation. Verbal approvals or no consent still occur in 2 to 5 TSUs. TSUs claimed to have procedures for mapping the average time required to solve technical problems (14/16). Thirteen TSUs have a contingency plan for equipment and connectivity failures. The existence of standardized procedures for communicating incidents was rated as satisfactory by 9/16 TSUs.

**Fig. 2:**
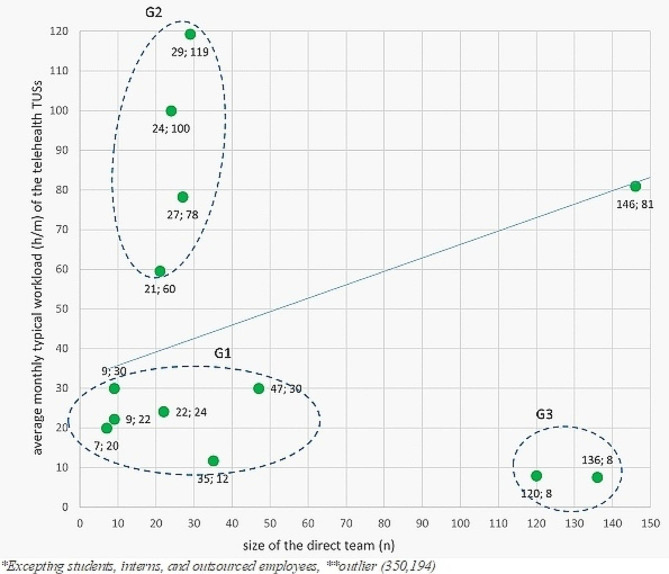
Relationship between the size of the direct team (n) and the average monthly typical workload (h/m) of the telehealth TSUs (n = 17; r = 14) with cluster analysis (G1 = 6, G2 = 4, G3 = 2, nongroup = 1)

About *human resources*, the profiles are teleconsultants (603), IT professionals (110), administrative assistants (97), teleregulators (33), technical-scientific researchers (30), general coordinators (19), education professionals/EAD (18), digital health professionals (18), specialists in artificial intelligence or data analysis (6), monitors (6), field coordinators (6), and digital law specialized attorneys (3). Three groups (G1, G2, and G3) and one distinct TSU were observed when the average workload of the permanent team was related to the number of professionals in the direct team of the TSUs (Fig. 2).

G1 is composed of TSUs with smaller teams and workloads, with an average of two hours a day. G2 comprises TSUs with teams comparable to G1 but with an average daily workload of approximately four hours. G3 is composed of TSUs with large teams but the lowest workloads. Most TSU offer team training (13/14). Training is conducted through documentation in 8/13 of these TSUs. They (11/14) have teams sufficiently qualified. Two TSUs indicated an insufficiency of professionals.

TSUs have permanent education policy (6/14) according to the responses to the theme *training, and outreach*. Emerging issues were continuing education in data protection rules (9/14), and security (10/14). Two-thirds of the TSUs offer training to requesting professionals (10/15).

Regarding the theme *infrastructure and technology*, the TSUs have an exclusive space for the secretariat (10/14) and an environment for the teleconsultant (9/14). Two-thirds use platforms developed locally (10/15). Most TSUs (9/15) use an integrated electronic health record (EHR-S) system, including EHR-S with the SUS’s clinical history (4/15) and registration (4/15); (9/14) units consider their storage space for the next two years satisfactory. There is a proper budget line for the administrative functions, a formal support in financial control and an appropriate line for the maintenance and acquisition of equipment and software in all TSUs. Additionally, noteworthy is the standard support for the needs of the data centre and its high performance (14/15).

For the *monitoring and evaluation* theme, the TSUs automatically monitored services (52%) and complementary manual assessment. Most TSUs account for municipality activities such as teleconsultation (13/17), inter-consultation (8/17), telediagnosis (9/17), and teleeducation (12/17). In addition, the TSUs reported counting by unit served, specialty, team, state of the federation, and synchronous versus asynchronous activities. The practice to conduct a satisfaction survey was undertaken (8/14). Only 3/14 TSUs conduct long-term opinion polls. The most frequent difficulties reported were the inadequacy of the hiring modality and funding discontinuities (8/14), high turnover of professionals and managers in the municipalities (5/14), and low profiles in TSUs and municipalities (3/14). One TSU reported the lack of a national platform with standardized services and poor connectivity quality.

The leaders engage with the team and professionals at the health centres served (12/13) according to the *acceptability and suitability* theme. Engagement with citizens/patients almost did not occur (3/13).

In *protection and security*, most TSUs claimed to record consent forms regularly for services (8/13); only a handful reported doing so in an incipient way (3/13), and 2/13 claimed that the process is not conducted. In 8/11 TSUs, there is a guarantee from the host institution in the strategies implemented to monitor the reliability of the data.

The TSUs maintain the confidentiality of the data provided (12/14), according to the responses to the topic *ethical and legal aspects*. The processing personal data comply with national regulations in 9/14 of the TSUs.

For *innovation and research network’s* theme, TSUs have formal link with research sectors (12/13); in 9/13 TSUs, there is a research department. According to the scenario regarding the TSUs’ participation in the National Health Data Network (RNDS), one unit reported full participation in building the RNDS data repository, and 3/14 TSUs with incipient participation. Considering health surveillance, 4/14 of the TSUs reported satisfactory integration between the RNDS, the app *Conecte SUS*, and the host institution in COVID-19 care procedures.

Planning is linked to the sustainability of TSU actions (12/14), according to the 12th theme, *citizenship, and sustainable development*. The MoH finances 11 of the 15 TSU; in seven, the MoH is the sole or significant funding source. Seven TSUs reported other primary funding sources. To achieve ways to promote citizen participation, they do EAD courses (4/14), advertising campaigns, webinars, and video channels (8/14).

### Global diagnosis

**Fig. 3:**
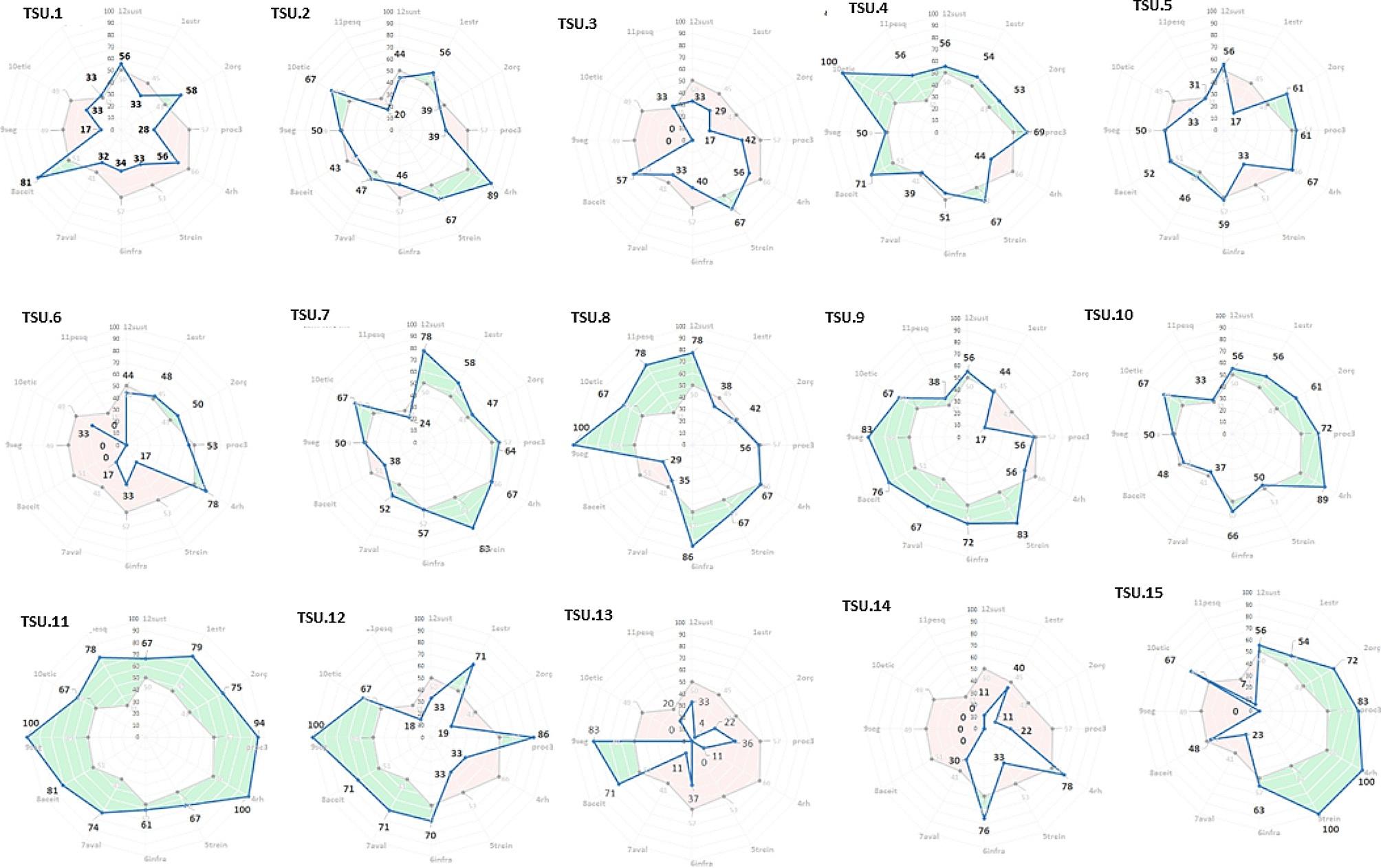
Summary of the diagnostic assessment of maturity by theme

We generated a radar chart for 15 units, presenting the TSU diagnostic evaluation, that allows a visual comparison of the interpreted results against the mean (Fig. 3). A percentage value represents the indicator for each of the twelve themes. The darkest area represents results above the TSUs means, while the other area represents results below the mean.

There were two assessment types: the respondent’s self-assessment and the researchers’ diagnostic assessment. The data collected refer to the services’ description and the level of maturity assessed by the TSUs.

Global results show the self-assessment of maturity as reported by respondents, the diagnostic evaluation produced throughout this study, the difference, and the group in which the TSUs were labeled based on the analysis of Groups M1, M2, and M3 that contextualize the maturity profiles (Table 2).

**Table 2:** Self-assessment (%) and diagnostic assessment (%) comparisons

#	TSU	Evaluated topics	Self-assessment (13 topics)	Diagnostic assessment (34 topics)	Difference	Group
**1**	1	34	38%	39%	1%	M3
**2**	2	34	51%	49%	-2%	M2
**3**	3	34	33%	35%	2%	M3
**4**	4	34	51%	55%	4%	M2
**5**	6	31	62%	48%	-14%	M2
**6**	7	26	62%	34%	-28%	M2
**7**	8	34	46%	57%	11%	M2
**8**	10	34	82%	62%	-20%	M1
**9**	11	34	64%	57%	-7%	M2
**10**	13	33	90%	57%	-33%	M1
**11**	14	34	87%	78%	-9%	M1
**12**	15	34	59%	56%	-3%	M2
**13**	16	21	44%	25%	-19%	M3
**14**	17	25	44%	32%	-12%	M3
**15**	19	33	85%	56%	-29%	M1
			**60%**	**52%**		**M1:4 TSU** **M2:7 TSU** **M3:4 TSU**

**Fig. 4:**
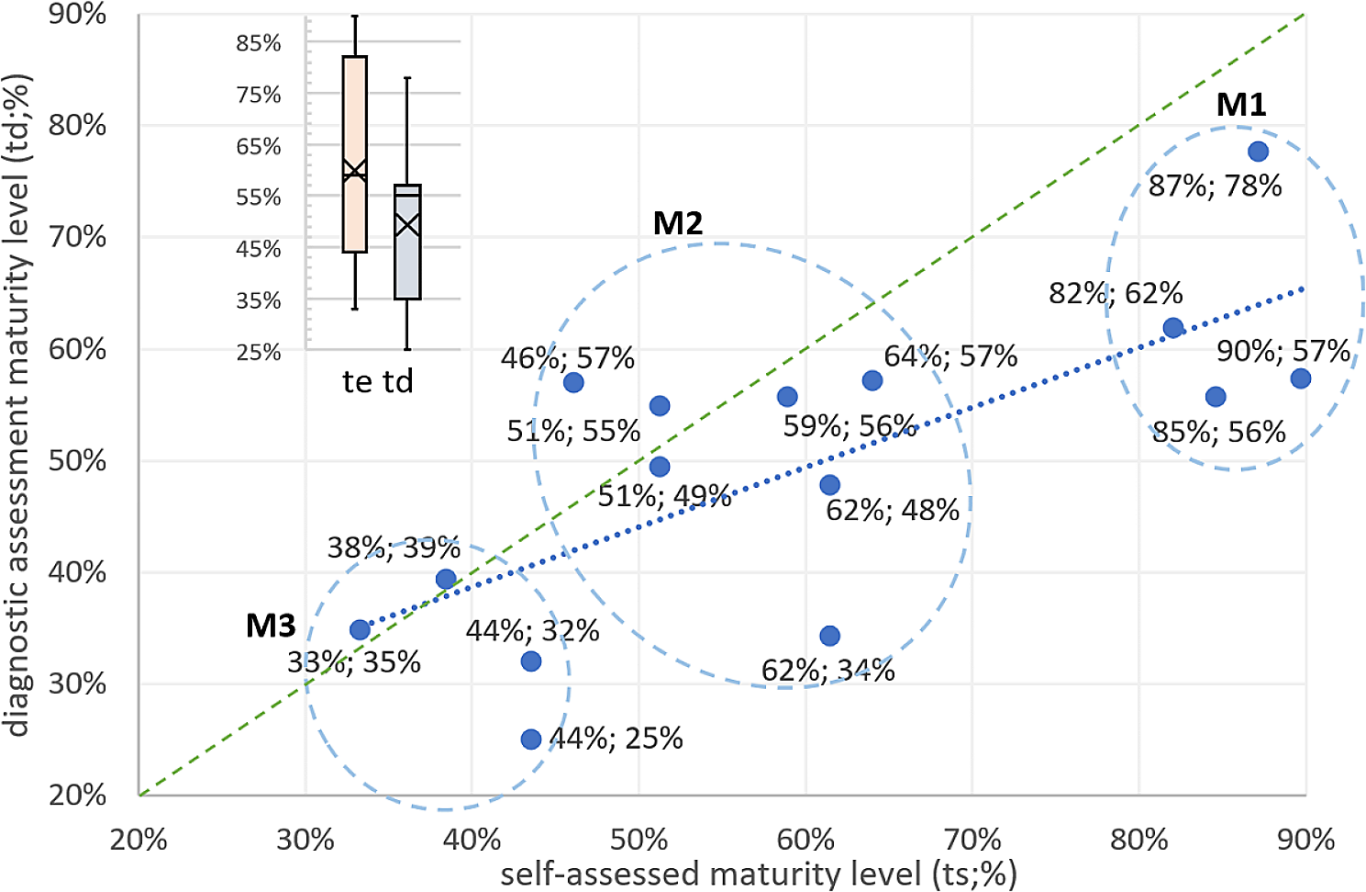
Maturity ratio (%) self-assessed of essential topics (et = 13) versus diagnostic assessment of topics (dt = 34) of telehealth service units (n = 17; r = 15) with cluster analysis (M1, M2, M3) and trend line (pointed)

The relationship between the self-assessment and the diagnostic assessment was measured (Fig. 4).

It is possible to observe the presence of three TSU groups concerning the maturity level assessment (maturity groups, M):

- M1: high; units that have an increased maturity regarding telehealth services, showing stability in the services’ offer and the projects’ participation.
- M2: intermediate; units whose telehealth services are already at a reasonable level, have room for improvement, and have valuable experience in the relationship and exchange of services with other TSUs.
- M3: low; units that present a level of lag in services, with a significant margin to evolve with the potential to form a network of services.

The groups’ interpretation should be made with the self-assessed and diagnostic assessment presented and Table 2. TSUs 7, 16, and 17, which had fewer assessed topics, were the ones that presented the most significant discrepancy between the self-assessed maturity level and the diagnostic assessment maturity level. This caused TSUs 16 and 17 to be grouped in the M3 group. TSU 7, on the other hand, was placed in the M2 group due to its higher level of self-assessment. This does not necessarily mean that the telehealth services of these TSUs are nascent. Instead, the final evaluation had interference due to the lack of completeness of the topics and that the unit has potential for improvement.

On the other hand, the TSU who responded to all topics had a greater tendency for self-assessed maturity levels and diagnostic assessment to be close. It is worth highlighting TSU 1 and 3, which remained in the M3 group but are new or have some level of difficulty in providing services; thus, they have a large margin for evolution. TSUs 10, 13, and 19 were placed in the M1 group. They had a high self-assessment level but a more significant discrepancy for the diagnostic evaluation.

For the M1 group, in which each TSU has a high maturity level concerning telehealth services, we can observe that from the four TSUs, only two (10 and 14) have a self-assessment, like the diagnostic assessment. The two TSUs in M1, which present a discrepant self-assessment from the diagnostic evaluation, have a difference that exceeds more than 30% in both units. For the M2 group with an intermediate maturity level, which has a total of seven TSUs, we can highlight that three have self-assessments that converge on the same maturity levels of the diagnostic assessment (2, 11, 15). Three TSUs in M2 (4, 6, 8) had a gap between the maturity levels presented in the self-assessment and the diagnostic assessment. In the M3 group with low self-assessment and diagnostic evaluation for maturity level, two TSUs (1 and 3) have maturity levels that converge on those of the self-assessment and diagnostic assessment, thus showing little difference between the two assessment processes. The other two TSUs in M3 (16 and 17) have discrepant maturity levels between the two assessments.

## Discussion

The diagnostic evaluation presents a picture of the telehealth’s growth. Other recent telehealth services’ evaluations in Brazilian establishments were conducted. One study describes the telehealth activities carried out by the Telessaúde RS/UFRGS program [27]. Remote inter-consultation, telediagnosis, and teleeducation activities are identified. In this way, the authors described actions, covering two themes proposed in the present work: structure and management and infrastructure and technology. This national network of TSUs was already in use in 2016 to implementing the e-SUS electronic medical record. We showed that TSUs continue to work with inter-consultation, telediagnosis, teleeducation, in addition to implementing teleconsultations.

In 2017, researchers started a qualitative study based on interviews with coordinators and professionals at Telessaúde Santa Catarina [28]. The authors analysed three main dimensions: organizational and management, knowledge and mastery of technology, and a comprehensive health care model. The survey identified that the physical and technological infrastructure was still insufficient. In need of improvements, the work process organization was inadequate, and there was no regulation. The present work indicates an evolution in the organization of operations, with the TSUs responding that there are protocols, and clinical guidelines, as well as some level of documentation and obtaining consent forms electronically. Even so, less than half of the TSUs reported having this type of organization of processes and activities. Our results went further and collected information regarding sufficient procedures to manage failures and incidents. Some professionals said that the program was still little known and that there needed to be knowledgeable of all available services. The services platform for continuous access was described as sufficient. The abovementioned investigation sought answers to questions about management, infrastructure, dissemination, training, and human resources. However, it did not address budget management, citizenship, sustainable development, networking, innovation, and research topics.

In 2022, one study analysed the digital maturity degree of 15 health centres in the northeast region of Brazil based on the Brazilian Digital Health Index (BDHI) [29] and a collection instrument with questions on eight themes: digital health policy and strategy; government investment and resources; legislation, policy and rules/regulations; resources; interoperability and security standards; technological infrastructure; services and applications; and citizenship, sustainability, and knowledge economy [30]. There were similar themes, such as citizenship, sustainability, security, and budget management, management and infrastructure. Of these, 66.7% offer teleeducation, 40% inter-consultation, and 26.7% telediagnosis, while the present study’s percentages were 94.1%, 82.3%, and 70.6%, respectively. The teleconsultations were identified in 26.7% of the centres, while in the present study, it was placed in 58.8%. Despite these results, it is impossible to infer an increase in the services since these are different samples.

Therefore, distinct requirements of telehealth service maturity can be seen to guarantee universality, equity in services, and comprehensiveness of populations’ care. A tool for accommodate several dimensions of maturity combined in a modular way is needed with a self-assessment module to help managers know the telehealth service readiness. Kingdon suggests that the president’s staff and bureaucrats are not ranked as highly regarding their influence on agenda-setting and that less visible actors play a more significant role in identifying specific alternatives to set the agenda [12]. In the case of telehealth in Brazil, this research shows that the voice and opinion of TSU managers can and has been improving public digital health policy in the country. Research has shown that scientific evidence only sometimes influences decisions to adopt innovations in health care. For many decision-makers, experiential knowledge can be more relevant and applicable.

A key limitation of the investigation was the impossibility of personally checking the facilities of the TSUs to audit the recorded responses of managers.

## Conclusions

We summarized a remote diagnostic evaluation that verified advances in telehealth services, such as the presence of a National Telediagnosis Platform and the monitoring of TSUs’ financial situation with cost-minimization and utility cost indicators. However, it highlighted challenges, such as the few long-term evaluation surveys, the need for informed consent for the services, and for knowledge of patients regarding telehealth.

The principal contribution to knowledge is the triangulation of methodologies to support evaluating health services. Although we found scientific evidence typically underpinned the adoption process, the types of evidence most valued by strategic-level decision-makers were insights into real-world implementation challenges and impact obtained from other jurisdictions. These findings contribute to recognized gaps in the literature, including sure how, when, and why different types of evidence are used during decisions to adopt innovations in health care.

### Electronic supplementary material

Below is the link to the electronic supplementary material.

Supplementary Material 1

Supplementary Material 2

Supplementary Material 3

Supplementary Material 4

## Data Availability

We declare that the supplementary data underlying this article are available in Zenodo at 10.5281/zenodo.7647591 and in SciELO Preprints Collection at 10.1590/SciELOPreprints.6416.

## Abbreviations

TSU: Telehealth Services Unit
SUS: Brazilian Unified Health System
MoH: Ministry of Health
IT: Information Technology
EAD: Education at Distance
UFRGS: Federal University of Rio Grande do Sul
RS: Rio Grande do Sul
BDHI: Brazilian Digital Health Index
