# Supplementary material for: Diagnostic evaluation of institutions as a basis for designing the Brazilian maturity model of telehealth services
Source: BMC Health Serv Res. 2024 Mar 25;24:372. doi: 10.1186/s12913-024-10723-8 (PMC10964504; doi:10.1186/s12913-024-10723-8)
Supplement: Supplementary file 1 — Supplementary Material 1 [file 12913_2024_10723_MOESM1_ESM.pdf]

Supplementary data 1: Aspects collected in the first stage of the research (review).

Aspects · Static report

Table of evaluation aspects of telehealth centers compiled from the literature

#	theme	category	aspect	nature	element(s)	ref
1	acceptability and adequacy	acceptability	suitability for context	feature	The solution is adapted to the local context and language. Answer: yes / no / not applicable / don't know	HAOC1, 2019 4,*239.1*
two	acceptability and adequacy	acceptability	work load	feature	The impact on the workload of health professionals health is compatible with their care practice. Answer: yes / no / not applicable / don't know	HAOC1, 2019 4,*234.1*
3	acceptability and adequacy	acceptability	engagement of leaders		Project leaders are engaged with the featureparticipants (health professionals and/or patients). Answer: yes / no / not applicable / don't know	HAOC1, 2019 4,*241.1*
4	acceptability and adequacy	acceptability	ease of use	feature	The solution is user-friendly. Answer: yes / no / not applicable / don't know	HAOC1, 2019 4,*238.1*
5	acceptability and adequacy	acceptability	integration	feature	The solution is integrated into the information registration system. Answer: yes / no / not applicable / don't know Note: Does not clearly specify a PEP.	HAOC1, 2019 4,*232.1*
6	acceptability and adequacy	acceptability	mechanisms for insertion		Mechanisms are developed to insert the feature solution in care practice. Answer: yes / no / not applicable / don't know	HAOC1, 2019 4,*231.1*
7	acceptability and adequacy	acceptability	speed	feature	The solution is quick to implement/use. Answer: yes / no / not applicable / don't know	HAOC1, 2019 4,*236.1*
8	acceptability and adequacy	acceptability	support	feature	There is a support/troubleshooting system available when needed. Answer: yes / no / not applicable / don't know	HAOC1, 2019 4,*235.1*
9	acceptability and adequacy	acceptability	response time	feature	The response time is adequate for the demand of the requesting professionals. Answer: yes / no / not applicable / don't know	HAOC1, 2019 4,*237.1*
10	acceptability and adequacy	acceptability	training		There is training or support/training mechanisms feature for use by healthcare professionals or patients. Answer: yes / no / not applicable / don't know	HAOC1, 2019 4,*233.1*
11	acceptability and adequacy	acceptability	usability		Some standardized/validated method is used to assess the usability of the solution. The main methods are: a) log: sensor-based data, manual scoring by researchers during observations or system log files; b) observation: video-based systems, featurescreen capture or supervision by experts in usability; c) questionnaires, which tend to be the most used method; d) interviews; e) self-descriptive: users perform tasks while thinking aloud and commenting on their experience and impressions during or shortly after the execution of each task. Answer: yes / no / not applicable / don't know	HAOC1, 2019 4,*240.1*
12	acceptability and adequacy	assessment	valued highlights of the activities of core	feature	a) the core capillarity in primary care in state; b) the ability of the nucleus to sustain itself with exclusive funding from the Ministry of Health; c) willingness to participate in the National Telediagnosis Offer; d) consolidation of the telediagnosis service, especially the ECG electrocardiogram report; e) the ability to reorganize the nucleus in the face of funding difficulties, obtaining various sources such as those linked to research projects and the State Health Department; f) its strong integration with research projects and the center's advances in the development of new technologies; h) 24/7 offer of services, in particular telediagnosis and referral contact; i) low turnover of teleconsultants in the nucleus; j) the consolidation of the 0800 as an important tool to support the clinic in Primary Care; k) working with the State Regulation Center; l) the qualification of the queues of specialties; m) the availability of the "Questions of the week" tool; n) the opportunity for problem-based learning from core offerings; o) the university's characteristic of promoting research and its innovative potential linked to services; p) strong integration with the State Health Department; q) the compulsory teleconsulting strategy applied to regulation; r) the capillarity of the issues from core offerings; o) the university's characteristic of promoting research and its innovative potential linked to services; p) strong integration with the State Health Department; q) the compulsory teleconsulting strategy applied to regulation; r) the capillarity of the issues from core offerings; o) the university's characteristic of promoting research and its innovative potential linked to services; p) strong integration with the State Health Department; q) the compulsory teleconsulting strategy applied to regulation; r) the capillarity of the	HAOC3, 2019 5.1, 5.2, 5.3, 5.4,*308.1*

#	theme	category	aspect	nature	element(s)	ref
					core actions in the state, which is a major contributor to the visibility of Primary Care in the territory; s) support for the implementation of the e-SUS AB;	
13	acceptability and adequacy	assessment	difficulties and challenges to core activities	feature	a) the absence of partnerships to expand support and funding for the nucleus' activities; b) the current priority of the State Health Department in medium and high complexity; c) the low responsiveness of the platform to use on cell phones; d) the long time spent filling out the teleconsultation request form and user registration; e) the teleconsultant does not know about activities offered other than those in which he is involved; f) absence of the role of the state government in financing and articulating with municipalities; g) difficulty in maintaining constant and face-to-face field work to monitor the health teams in the municipalities, in view of funding difficulties; h) some teleconsultations carried out demonstrate the fragility of the teams in relation to their work process, mainly the lack of articulation between the professionals of the team itself, constituting a problem that teleconsultants need to deal with; i) the core's internal teams work in a segmented way, with little melee, exchange and synergy; j) the fact that the main axis of the 0800 offer is medical doctors, being limited to professionals from other categories, so fundamental to primary care; k) difficulty for health professionals to use access protocols because they did not participate in its construction process; l) access to the regulation flow for matrix support is only possible for the family doctor and not for the specialist (specialist access is not possible because it is a specific service to support primary care teams); m) underreporting of teleconsultations through the use of other communication tools WhatsApp®) among physicians; n) maintenance of equipment for telediagnosis, which is the municipal counterpart for the service offered, but which often does not happen; o) care for data confidentiality; p) difficulties in understanding teledermatology protocols, for example, which require attention because they are thorough;	HAOC3, 2019 5.1, 5.2, 5.3, 5.4,*309.1*
14	acceptability and adequacy	assessment	level of satisfaction	indicator	Level of user satisfaction with the service	DESD, 2021 , *1806.1*
15	acceptability and adequacy	assessment	resistance to telediagnosis requests	feature	Evaluation of resistance by telediagnosis requesters.	HAOC3, 2019 section 4.4,*306.1*
16	acceptability and adequacy	assessment	resolvability of teleconsulting	feature	The platform has an evaluation questionnaire to be completed by the user, as an integral part of closing the cycle opening x finalizing the teleconsultation: Did the teleconsultation answer answer your question? (completely met; partially met; did not meet);	NTSHU UFMA, 2019 4.6,*493.1*
17	acceptability and adequacy	assessment	applicant satisfaction with teleconsulting	feature	The platform has an evaluation questionnaire to be completed by the user, as an integral part of closing the opening x finalizing cycle of the teleconsultation: What is your degree of satisfaction? (very satisfied; satisfied; indifferent; dissatisfied; very dissatisfied).	NTSHU UFMA, 2019 4.6,*494.1*
18	acceptability and adequacy	assessment	utility and satisfaction of SOF	feature	To evaluate the SOF, there must be a tool to measure the usefulness and satisfaction of the user regarding the content found in the SOF. This evaluation should be done through a quick questionnaire and the results should be available for viewing by everyone who accesses the SOF.	MSNT63, 2014 7,*330.1*
19	acceptability and adequacy	short review term	specialties by unit	indicator	The number of specialties per unit must be increased. Will measure acceptance among physicians and hospital administrators	PAHO, 2016 , *155.1*
20	acceptability and adequacy	short review term	patient satisfaction	indicator	patient satisfaction	PAHO, 2016 , *159.1*
21	acceptability and adequacy	democracy, control social and justice cognitive	sharing of knowledge	feature	Formulation of strategies for the democratization of health information and IT that reveal the relationships between living conditions and health situation, in intelligible language for the universe of the population and the health counselors representing the users	ABRASCO, 2020 5th dimension, *529.1*

#	theme	category	aspect	nature	element(s)	ref
					from SUS. Disseminate the topic of health information and the SUS budget discussion.	
22	acceptability and adequacy	democracy, control social and justice cognitive	verification of authenticity of information	feature	The construction, together with the institutional instances of social control of health, of mechanisms to verify the authenticity of health information disseminated through digital media.	ABRASCO, 2020 5th dimension, *530.1*
23	acceptability and adequacy	efficiency	patient's perception	indicator		PAHO, 2016 , *193.1*
24	acceptability and adequacy	endogenous	patient experience	indicator	reported by the patient	PAHO, 2016 , *199.1*
25	acceptability and adequacy	endogenous	trained professionals	indicator	trained technical staff	PAHO, 2016 , *197.1*
26	acceptability and adequacy	project stage	team acceptance	indicator		PAHO, 2016 , *181.1*
27	acceptability and adequacy	project stage	acceptance by the health team	indicator		PAHO, 2016 , *172.1*
28	acceptability and adequacy	project stage	patient acceptance	indicator		PAHO, 2016 , *179.1*
29	acceptability and adequacy	project stage	accessibility to the patient	indicator		PAHO, 2016 , *178.1*
30	acceptability and adequacy	project stage	accessibility to the professional	indicator		PAHO, 2016 , *180.1*
31	acceptability and adequacy	project stage	awareness of authorities and Decision makers	indicator		PAHO, 2016 , *173.1*
32	acceptability and adequacy	exogenous	service access	indicator		PAHO, 2016 , *201.1*
33	acceptability and adequacy	governance and management of information and technology of health information	information and technology as a public good	feature	Adopt the concept of public good for information systems, file services, repositories archives, platforms and applications defined as complex, relevant and strategic for the SUS, in its tangible and intangible dimensions.	ABRASCO, 2020 1st dimension, 8,*507.1*
34	acceptability and adequacy	governance and management of information and technology of health information	integration with others sectors for definition of the data	feature	It must articulate with other sectors in order to carry out the collection, processing, treatment and progressive dissemination of classifications and variables "occupation" and "economic activity" in the SIS, considered strategic and of interest for the analysis of the social determination of the health-disease-care process.	ABRASCO, 2020 1st dimension, 17,*515.1*
35	acceptability and adequacy	governance and resources organizational	digital health culture	feature	Organizational culture: There is a culture in the institution that favors and encourages the adoption of technologies fingerprints.	IMDS, 2021 , *686.1*
36	acceptability and adequacy	governance and resources organizational	digital strategy	feature	Institutional Support: There is indeed support from the Board and/or Council for the digital transformation.	IMDS, 2021 , *692.1*
37	acceptability and adequacy	identification of needs in health	participation of healthcare professionals and patients	feature	The assessment of health needs relies on the participation of health professionals and patients.<br><br>Answer: yes / no / not applicable / don't know	HAOC1, 2019 1,*217.1*
38	acceptability and adequacy	indicators	Net Promoter Score NPS	indicator	Using the Net Promoter Score, both for end-users (patients) and healthcare professionals, can help to embed innovation.<br>Note: Not included in the questionnaire.	HAOC1, 2019 6,*260.1*
39	acceptability and adequacy	indicators	satisfaction of participants	indicator	a) satisfaction of health professionals; b) patient satisfaction; Numerous works demonstrate that telehealth services are well evaluated. However, this is not reflected in equally matched utilization rates. In fact, satisfaction is very little useful information to be used as an outcome, and at most it should guide the monitoring of services. Note: Not included in the questionnaire.	HAOC1, 2019 6,*262.1*
40	acceptability and adequacy	monitoring and audit	ombudsman	feature	There is an ombudsman service or systematic research with users to evaluate solutions and identify possible barriers.<br>Answer: yes / no / not applicable / don't know	HAOC1, 2019 5,*248.1*
41	acceptability and adequacy	the user as protagonist	actions for involvement of citizens	feature	a) develop a channel of instructional videos on the use of citizen applications and case examples from real life; b) hold webinars in partnership with the National Health Council, with emphasis on the User Forum and other representatives of civil society, to present to Social Control the benefits of using	ESD28, 2020 4.1.1,*717.1*

#	theme	category	aspect	nature	element(s)	ref
					digital health solutions; c) develop and publicize free EaD courses for citizens and offer them on the Government's digital platforms; d) carry out publicity campaigns to disseminate Digital Health actions to citizens; e) establish a specific flow and service at the SUS Ombudsman for Digital Health services.	
42	acceptability and adequacy	the user as protagonist	activities for develop actions to the involvement of Health professionals	featurebut that, above all, allow them to gain experience to	a) identify actors with knowledge, experience and interest in the topic; b) define a range of essential projects to obtain concrete results, advance this priority in a systematic way; c) document and disseminate accumulated knowledge and transform it into action.	ESD28, 2020 4.1.2,*718.1*
43	acceptability and adequacy	the user as protagonist	action plan	feature your health, your family and your community, in addition to	4. Engagement of patients and citizens, to promote the adoption of healthy habits and the management of assist in building the information systems they will use.	ESD28, 2020 4,*715.1*
44	acceptability and adequacy	the user as protagonist	priorities	feature	4. The user as protagonist 4.1 User engagement 4.1.1 Develop actions to involve citizens 4.1.2 Develop actions to involve health professionals 4.2 Information platforms for citizens and users 4.2.1 Implement personal health record services	ESD28, 2020 4,*716.1*
45	acceptability and adequacy	preference of receivers of care	informed choice	feature	Has the healthcare organization provided easy access to the necessary information so that appropriate choices can be made between the various healthcare delivery options, enabling the care recipient or their representative to: a) express informed choices from the completion of specific health activities; b) request the realization health care activities that do not use telehealth services; c) refuse to allow specific health care activities to be carried out through telehealth services; d) change your preferences and switch to another mode of health care delivery.	ISO13131, 2021 11.1.4,*64.1*
46	acceptability and adequacy	preparation organizational	acceptance by the health team	feature comfort of the members of the health team to	Participatory dialogues promote security and telehealth services.	ARGMNMM, 2020 I.26,*568.1*
47	acceptability and adequacy	preparation organizational	acceptance of beneficiaries	feature	A level of acceptance of telehealth services by potential beneficiaries is expected.	ARGMNMM, 2020 I.37,*579.1*
48	acceptability and adequacy	preparation organizational	digital literacy of patients	feature	The digital literacy level of potential patients is well known.	ARGMNMM, 2020 I.40,*582.1*
49	acceptability and adequacy	preparation organizational	cultural barrier	feature difficulties during the provision of services	Is there a cultural or linguistic barrier that cause telehealth.	ARGMNM, 2020 I.38,*580.1*
50	acceptability and adequacy	preparation organizational	communication to beneficiaries	of the featuretelehealth services about their opening or	The institution communicates to potential beneficiaries fortification.	ARGMNMM, 2020 I.35,*577.1*
51	acceptability and adequacy	preparation organizational	internal communication	featureintention to implement or strengthen the services of	People at the institution were informed about the telehealth.	ARGMNMM, 2020 I.33,*575.1*
52	acceptability and adequacy	preparation organizational	connectivity of patients	feature	The connectivity level of potential patients is well known.	ARGMNMM, 2020 I.39,*581.1*
53	acceptability and adequacy	preparation organizational	medical team participant	feature	The medical team agrees with the provision of telehealth services.	ARGMNMM, 2020 I.23,*565.1*
54	acceptability and adequacy	preparation organizational	non-medical staff participant	feature	The non-medical team agrees with offering telehealth services.	ARGMNMM, 2020 I.24,*566.1*
55	acceptability and adequacy	preparation organizational	change management	health team featureso that its members feel safe and	There is management of changes in the routines of the comfortable to work in telehealth services.	ARGMNMM, 2020 I.25,*567.1*
56	acceptability and adequacy	preparation organizational	incentive for institution	feature	There is an incentive mechanism for the institution to use telehealth.	ARGMNMM, 2020 I.28,*570.1*
57	acceptability and adequacy	preparation organizational	incentive for telehealth	featureThe institution establishes incentive mechanisms for	the use of telehealth.	ARGMNMM, 2020

#	theme	category	aspect	nature	element(s)	ref
						I.27,*569.1*
58	acceptability and adequacy	process	ombudsman	feature	There are procedures and tools for the health team to share their concerns, suggestions and comments about the development of the telehealth program.	ARGMNMM, 2020 I.52,*594.1*
59	acceptability and adequacy	process	ombudsman	feature	There are procedures and tools for patients to share their concerns, suggestions and comments about the telehealth program.	ARGMNMM, 2020 I.53,*595.1*
60	acceptability and adequacy	process	contingency plan	use telemarketing consider that	Are there procedures or plans for when featureprofessionals who a face-to-face assessment is required.	ARGMNMM, 2020 I.56,*598.1*
61	acceptability and adequacy	process	patient satisfaction	feature	There are procedures for recording patient satisfaction of telehealth services.	ARGMNMM, 2020 I.48,*590.1*
62	acceptability and adequacy	production	satisfaction of applicants for teleconsultations	graphic	Graph 10. Degree of Satisfaction of Teleconsultation responses July/2018.	HAOC2, 2019 4.2,*283.1*
63	acceptability and adequacy	service design of health	health service design	featureyour plans for telehealth services, your	The health organization includes care recipients and their representatives in the community in consultations about purposes, projects, operations, management and access to telehealth services	ISO13131, 2021 11.1.8,*70.1*
64	acceptability and adequacy	quality	patient satisfaction	indicator	Determines the satisfaction of patients assisted by telemedicine. Interpretation: Determines the user's perception after using telemedicine services. It helps to improve the functioning of the program. Formula: Number of satisfied patients x 100/total of patients assisted by telemedicine. Frequency: Monthly. Indicator Type. Quality. Notes: A simple satisfaction survey based on Likert scale responses is required. It can also be used by doctors.	PAHO, 2016 , *211.1*
65	acceptability and adequacy	results and assessment	assessment of satisfaction with objects of tele-learning education	indicator	Description: global assessment of job satisfaction with learning objects per month; Numerator: Likert scale (with 5 levels) of applicant satisfaction; Unit: levels of satisfaction; Source: data from tele-education offers;<br><br>Note: For the classification of tele-education topics, BIREME's DeCS must be used. Note: the evaluation of the professionals' satisfaction will be done by accessing courses, seminars and web conferences.	MSNT5, 2014 annex I, frame 6,*422.1*
66	acceptability and adequacy	results and assessment	evaluation of satisfaction in tele-education	indicator	Description: global assessment of the satisfaction of participating professionals per month; Numerator: Likert scale (with 5 levels) of applicant satisfaction; Unit: levels of satisfaction; Source: data from tele-education offers;<br><br>Note: For the classification of tele-education topics, BIREME's DeCS must be used. Note: the evaluation of the professionals' satisfaction will be done by accessing courses, seminars and web conferences.	MSNT5, 2014 annex I, frame 6,*420.1*
67	acceptability and adequacy	services of healthcare suitable	suitability of services of health	exclusion; b) assessment by	The healthcare organization ensures that: a) healthcare using telehealth services is suitable for care recipients based on criteria of featuredocumented inclusion or health professional of the adequacy of the provision of health care using telehealth services continues during the activity.	ISO13131, 2021 11.1.6,*67.1*
68	acceptability and adequacy	systems and services, patterns and interoperability	applications for the patient	feature	Patient Applications: The institution has mobile applications or portals to promote patient engagement and/or facilitate access to information by professionals.	IMDS, 2021 , *672.1*
69	acceptability and adequacy	technology	access	feature	The solution has the ability to improve access for the target population.<br>Answer: yes / no / not applicable / don't know	HAOC1, 2019 two,*219.1*
70	acceptability and adequacy	technology	suitability for context	feature	The solution is adaptable to the existing scenario and structure.<br>Answer: yes / no / not applicable / don't know	HAOC1, 2019 two,*224.1*
71	acceptability and adequacy	technology	suitability for the public target	feature	The solution is adaptable to the target population.<br>Answer: yes / no / not applicable / don't know	HAOC1, 2019 two,*225.1*
72	acceptability and adequacy	technology	participation of health professionals and	feature	The choice and format of the solution are discussed with the health professionals and/or patients.	HAOC1, 2019 two,*226.1*

#	theme	category	TheOP Oients	nature	Rleesmfootao:(yes) / no / not applicable / don't know	ref
73	acceptability and adequacy	technology	use of evidence scientific	feature	Incorporation of the solution is based on evidence scientific evidence of moderate or high quality.<br>Answer: yes / no / not applicable / don't know	HAOC1, 2019<br>two,*221.1*
74	acceptability and adequacy	technology	use of solutions previously tested	feature	The telehealth service seeks solutions previously Tested to resolve the issue<br>Answer: yes / no / not applicable / don't know	HAOC1, 2019<br>two,*218.1*
75	acceptability and adequacy	user like protagonist	patient satisfaction	indicator	Permanent monitoring, evaluation and auditing of the population's level of satisfaction regarding their experience with digital health services, based on the creation of functional interfaces, with an intuitive design and that follow the principles of user experience, considering the diversity of target audiences ;	PNIIS, 2021<br>chapter II, section IV, art 7, IV, *795.1*
76	legal aspects and ethical	agents of data processing	responsibility		Art. 42. The controller or operator who, due to the exercise of personal data processing activity, causes property, moral, individual or collective damage to others, in violation of personal data protection legislation, is obliged to repair it. it. § 1 In order to ensure effective compensation to the data subject: I the operator is jointly and severally liable for damages caused by the treatment when it fails to comply with the obligations of data protection legislation or when it has not followed the lawful instructions of the controller, in which case the operator is equivalent to the controller, except in the cases of exclusion provided for in art. 43 of this Law; II the controllers who are directly involved in the treatment which caused damage to the data subject are jointly and severally liable, except in the cases of exclusion provided for in art. 43 of this Law. § 2 The judge, in civil proceedings, may reverse the burden of proof in favor of the data subject when, in his opinion, the allegation is credible, there is insufficient sufficiency for the purpose of producing evidence or when the production of evidence by the data subject results in excessively burdensome. § 3 Actions for reparation for collective damages whose object is liability under the terms of the caput of this article may be exercised collectively in court, observing feature the provisions of the relevant legislation. § 4 The person who repairing the damage to the holder has the right of recourse against the other responsible parties, to the extent of their participation in the harmful event. Art. 43. Processing agents will only be held liable when they prove: I that they did not carry out the processing of personal data assigned to them; II that, although they carried out the processing of personal data assigned to them, there was no violation of data protection legislation; or III - that the damage is due to the sole fault of the data subject or a third party. Art. 44. The processing of personal data will be irregular when it fails to observe the legislation or when it does not provide the security that the data subject can expect, considering the relevant circumstances, including: I the way in which it is carried out; II the result and the risks reasonably expected of it; III the personal data processing techniques available at the time it was carried out. Single paragraph. The controller or operator who, by failing to adopt the security measures provided for in art. 46 of this Law, causes the damage. Article 45.	LGPD, 2018<br>chapter VI, section III, *147.1*
77	legal aspects and ethical	agents of data processing	data processing	feature	Art. 39. The operator must carry out the treatment according to the instructions provided by the controller, who will verify compliance with the instructions and the rules on the matter.	LGPD, 2018<br>cap VI, art 39, *144.1*
78	legal aspects and ethical	legal aspects and ethical	local norms	feature	The solution complies with local regulations and current legislation.<br>Answer: yes / no / not applicable / don't know	HAOC1, 2019<br>3,*230.1*
79	legal aspects and ethical	political aspects	assessment of indicators	feature	Are there any routines from telehealth service leaders for evaluation of indicators and replanning.<br>Answer: yes / no / not applicable / don't know	HAOC1, 2019<br>7,*269.1*
80	legal aspects and ethical	political aspects	coma compatibility health policies	feature	The solution is compatible with the health policies of systems/services in which it is embedded.<br>Answer: yes / no / not applicable / don't know	HAOC1, 2019<br>7,*264.1*

#	theme	category	aspect	nature	element(s)	ref
81	legal aspects and ethical	political aspects	government participation	feature	There is collaboration, commitment and/or involvement of government officials. Answer: yes / no / not applicable / don't know	HAOC1, 2019 7,267.1
82	legal aspects and ethical	political aspects	strategic audience participation	featureof the strategic public (stakeholders).	There is collaboration, commitment and/or involvement Answer: yes / no / not applicable / don't know	HAOC1, 2019 7,266.1
83	legal aspects and ethical	political aspects	accountability	feature	There is promotion, dissemination and accountability for funders and sponsors of the telehealth service. Answer: yes / no / not applicable / don't know	HAOC1, 2019 7,268.1
84	legal aspects and ethical	regulatory aspects	patient's right	feature	Health team members are aware of their obligations arising from patients' rights.	ARGMNMM, 2020 V.101,641.1
85	legal aspects and ethical	regulatory aspects	georeferencing	featurepatients (country, state, county) who use services	Does the institution know the correct geolocation of of telehealth.	ARGMNMM, 2020 V.105,645.1
86	legal aspects and ethical	regulatory aspects	regulation mark	feature	The health team is aware of the national rules applicable to teleconsultation.	ARGMNMM, 2020 V.100,640.1
87	legal aspects and ethical	regulatory aspects	regulation mark	feature	The institution has in-house legal advice or access to a legal consulting service specializing in legality, ethics, privacy and security in telehealth.	ARGMNMM, 2020 V.104,644.1
88	legal aspects and ethical	regulatory aspects	regulation mark	norms for the regulation of	The institution understands that new featurelegal telehealth services.	ARGMNMM, 2020 V.107,647.1
89	legal aspects and ethical	regulatory aspects	data protection	obligations arising from data protection laws	The members of the health team know the feature personal data, and what data is sensitive.	ARGMNMM, 2020 V.102,642.1
90	legal aspects and ethical	regulatory aspects	responsibility of legal entities and physical in telemedicine	feature	Art. 17. Legal entities that provide telemedicine services, communication platforms and data archiving must have their headquarters established in Brazilian territory and be registered with the Regional Council of Medicine of the State where they are headquartered, with the respective technical responsibility of a regularly registered physician on the same Council. § 1 In case the provider is an individual, he must be a doctor duly registered with the Regional Council of Medicine of his jurisdiction and inform the entity of his option to use telemedicine. § 2 The investigation of any ethical violation of this resolution will be made by the CRM of the patient's jurisdiction and judged in the CRM of the responsible physician's jurisdiction.	CFM2314, 2022 art 17,1856.1
91	legal aspects and ethical	knowledge skilled	regulation mark	feature	There is a regulatory framework for the implementation of telehealth.	ARGMNMM, 2020 V.115,655.1
92	legal aspects and ethical	consent informed	authorization from attendance by telemedicine	feature	Art. 15. The patient or his legal representative must authorize the telemedicine service and the transmission of his images and data by means of (agreement and authorization term) free and informed consent, sent by electronic means or recording of reading of the text with the agreement, which must be part of the patient's SRES. Single paragraph. In all telemedicine care, explicit consent must be ensured, in which the patient or his legal representative must be aware that his personal information may be shared and your right to withhold permission to do so, except in a medical emergency.	CFM2314, 2022 art 15,1854.1
93	legal aspects and ethical	expenses of receivers of care	consideration financial for the service rendered	consideration for the service	Art. 16 The provision of telemedicine services, as a medical care method, in any modality, must follow the usual normative and ethical standards of face-to-face care, including in featureregarding the financial provided. Single paragraph. The doctor must agree in advance with the patient and health providers the value of the care provided, as in the personal assistance.	CFM2314, 2022 art 16,1855.1
94	legal aspects and ethical	rights over the data	data access	featureArt. 18. The holder of personal data has the right to obtain	of the controller, in relation to the subject's data processed by him, at any time and upon request: I confirmation of the existence of treatment; II access to data; III correction of incomplete data,	LGPD, 2018 chapter III, art 18, 126.1

#	theme	category	aspect	nature	element(s)	ref
					inaccurate or out of date; IV anonymization, blocking or deletion of data that is unnecessary, excessive or treated in violation of the provisions of this Law; V data portability to another service or product provider, upon express request and observing commercial and industrial secrets, in accordance with the regulation of the controlling body; V data portability to another service or product provider, upon express request, in accordance with the regulations of the national authority, observing commercial and industrial secrets; VI elimination of personal data processed with the consent of the holder, except in the cases provided for in art. 16 of this Law; VII information on public and private entities with which the controller carried out shared use of data; VIII information on the possibility of not providing consent and on the consequences of the refusal; IX revocation of consent, pursuant to § 5 of art. 8 of this Law. § 1 The holder of personal data has the right to petition in relation to their data against the controller before the national authority. § 2º The data subject may object to treatment carried out based on one of the hypotheses of waiver of consent, in case of non-compliance with the provisions of this Law. , the treatment agent. § 4 In case of impossibility of immediate adoption of the measure referred to in § 3 of this article, the controller will send the holder a response in which he may: I inform that he is not a data processing agent and indicate, whenever possible, the agent; or II indicate the factual or legal reasons that prevent the immediate adoption of the measure. § 5 The request referred to in § 3 of this article will be met at no cost to the holder, within the deadlines and under the terms set forth in the regulation. § 6 The person in charge must immediately inform the processing agents with whom the data has been shared with the correction, deletion, anonymization or blocking of the data, so that they repeat the same procedure, except in cases where this communication is demonstrably impossible or involves disproportionate effort. anonymized by the controller. § 8 The right referred to in § 1 of this article may also be exercised before consumer protection bodies.	
95	legal aspects and ethical	rights over the data	confirmation of existence or access	feature	Art. 19. Confirmation of existence or access to personal data will be provided, upon request by the holder: I in simplified format, immediately; or II by means of a clear and complete statement, which indicates the origin of the data, the lack of registration, the criteria used and the purpose of the treatment, observing commercial and industrial secrets, provided within a period of up to 15 (fifteen) days, counted the date of application by the holder. § 1 Personal data will be stored in a format that favors the exercise of the right of access. § 2 The information and data may be provided, at the holder's discretion: I by electronic means, safe and suitable for this purpose; or II in printed form. § 3 When the treatment has its origin in the consent of the holder or in a contract, the holder may request a complete electronic copy of his personal data, observing commercial and industrial secrets, in accordance with the regulations of the national authority, in a format that allows its subsequent use, including in other processing operations. § 4 The national authority may have different provisions regarding the deadlines provided for in items I and II of the main section of this article for specific sectors.	LGPD, 2018 chapter III, art 19, *127.1*
96	legal aspects and ethical	rights over the data	defense of interests and rights	or collectively, in	Art. 22. The defense of the interests and rights of data subjects may be exercised in court, featureindividually accordance with the provisions of relevant legislation, regarding the instruments of individual and collective protection.	LGPD, 2018 chapter III, art 22, *130.1*

#	theme	category	aspect	nature	element(s)	ref
97	legal aspects and ethical	rights over the data	patient's right to data	feature	Art. 3º § 6º It is the right of the patient or his representative legal to request and receive a copy on digital media and/or printout of your registration data.	CFM2314, 2022 art 3,*1842.1*
98	legal aspects and ethical	rights over the data	decision review	feature	Art. 20. The data subject has the right to request the review of decisions taken solely on the basis of automated processing of personal data that affect his interests, including decisions aimed at defining his personal, professional, consumption and credit profile or the aspects of your personality. § 1 The controller must provide, whenever requested, clear and adequate information regarding the criteria and procedures used for the automated decision, observing commercial and industrial secrets. § 2 In case of non-offering of information referred to in § 1 of this article based on the observance of commercial and industrial secrecy, the national authority may carry out an audit to verify discriminatory aspects in automated processing of personal data.	LGPD, 2018 chapter III, art 20, *128.1*
99	legal aspects and ethical	rights over the data	ownership	feature	Art. 17. Every natural person is assured the ownership of their personal data and guaranteed the fundamental rights of freedom, intimacy and privacy, under the terms of this Law.	LGPD, 2018 chapter III, art 17, *125.1*
100	legal aspects and ethical	rights over the data	data usage	feature	Art. 21. Personal data regarding the exercise regular rights by the holder cannot be used to your detriment.	LGPD, 2018 chapter III, art 21, *129.1*
101	legal aspects and ethical	structure	regulatory aspects	feature	Regulatory aspects: rules and procedures that must be considered in the provision of services of telehealth.	ARGMNMM, 2020 1.4.1,*537.1*
102	legal aspects and ethical	ethics, privacy and confidentiality	professional ethics in digital health	feature	Develop information policies that ensure structures and mechanisms for agents' conduct involved in health records; in development of ITIS; and in the management of health information within the scope of services.	ABRASCO, 2020 4th dimension, *525.1*
103	legal aspects and ethical	ethics, privacy and confidentiality	qualification of health agents	feature	Develop the qualification of health agents - public and outsourced - for changing the habitus in relation to the use of mechanisms and structures to ensure the authenticity of records, maintenance of these records in the chain of custody and the security of these records in health information systems.	ABRASCO, 2020 4th dimension, *528.1*
104	legal aspects and ethical	ethics, privacy and confidentiality	regulation on the use of information	feature	ITIS will be contemplated, within the scope of health policies, as a space for practices and knowledge that impact behaviors and value systems and beliefs of humans in the generation, dissemination and use of health information.	ABRASCO, 2020 4th dimension, *524.1*
105	legal aspects and ethical	governance and management of information and technology of health information	public interest in market expansion	feature	The market expansion of the economic-industrial complex of information technology and telecommunications must be in line with the interest of the res publica, with the documents agreed for health management, for social control and for the normative framework within the scope of the State's health and social security policy.	ABRASCO, 2020 1st dimension, 3,*502.1*
106	legal aspects and ethical	governance and resources organizational	patient privacy	feature	Privacy: The institution adheres to the legislation, norms and best practices for preserving the patient privacy.	IMDS, 2021 , *685.1*
107	legal aspects and ethical	legislation, policy and norms/regulations	legal basis for data protection	indicator	Legal basis for data protection (security): There is a law on data security (storage, transmission, use) that is relevant to digital health.	BDHI, 2021 5,*443.1*
108	legal aspects and ethical	legislation, policy and norms/regulations	sharing of data	feature	Secure cross-border sharing of data: There are protocols, policies, frameworks or processes that support the secure cross-border exchange and storage of data. This includes health data entering and leaving the country or relating to a foreign individual.	BDHI, 2021 8,*446.1*
109	legal aspects and ethical	legislation, policy and norms/regulations	data privacy	feature	Laws or regulations on privacy, confidentiality and access to health information (privacy): There is a law to protect privacy individual relating to ownership, access, and sharing of individually identifiable digital health data.	BDHI, 2021 6,*444.1*
110	legal aspects and ethical	preparation organizational	permission to telehealth services	feature	Telehealth services are allowed by national regulatory frameworks.	ARGMNMM, 2020

#	theme	category	aspect	nature	element(s)	ref
						I.6,*543.1*
111	legal aspects and ethical	Privacy	data rights		Privacy rules for telehealth technology feature(selective application of the Portability Law and HIPAA Health Insurance Liability	ICT-health, 2021 Use of technologies of information and from the communication in the area of health: a telehealth in 2021,*844.1*
112	legal aspects and ethical	regulation	regulation of services	feature	Licensing: Regulation of telehealth across states and by more types of healthcare professionals	ICT-health, 2021 Use of technologies of information and from the communication in the area of health: a telehealth in 2021,*845.1*
113	legal aspects and ethical	responsibility	responsibility for data preservation of telemedicine	feature	Art. 3º § 3º The anamnesis and work-up data, the results of complementary exams and the medical conduct adopted, related to the care provided by telemedicine must be preserved, in accordance with current legislation, under the custody of the physician responsible for the care in his own office or the director /technical manager, in the case of intervention by a company and/or institution. Art. 3º § 4º In case of contracting outsourced archiving services, the responsibility for keeping patient data and care must be contractually shared between the doctor and the contractor.	CFM2314, 2022 art 3,*1841.1*
114	legal aspects and ethical	security of information	governance	feature	Art. 50. Controllers and operators, within the scope of their competences, for the processing of personal data, individually or through associations, may formulate rules of good practices and governance that establish the organizational conditions, the functioning regime, the procedures, including holders' complaints and petitions, security norms, technical standards, specific obligations for the various parties involved in the treatment, educational actions, internal mechanisms for supervision and risk mitigation and other aspects related to the processing of personal data. § 1 When establishing rules of good practices, the controller and the operator will take into account, in relation to the processing and data, the nature, scope, purpose and probability and severity of the risks and benefits arising from the processing of data of the holder. § 2 In the application of the principles indicated in items VII and VIII of the caput of art. 6 of this Law, the controller, observing the structure, scale and volume of its operations, as well as the sensitivity of the data processed and the probability and severity of damage to data subjects, it may: I implement a privacy governance program that, at a minimum: a) demonstrates the commitment of the controller to adopt internal processes and policies that ensure comprehensive compliance with rules and good practices relating to the protection of personal data; b) is applicable to the entire set of personal data under its control, regardless of how it was collected; c) be adapted to the structure, scale and volume of its operations, as well as the sensitivity of the data processed; d) establish adequate policies and safeguards based on a systematic assessment process of impacts and risks to a) demonstrates the commitment of the controller to adopt internal processes and policies that ensure comprehensive compliance with rules and good practices related to the protection of personal data; b) is applicable to the entire set of personal data under its control, regardless of how it was collected; c) be adapted to the structure, scale and volume of its operations, as well as the sensitivity of the data processed; d) establish adequate policies and safeguards based on a systematic assessment process of impacts and risks to a) demonstrates the commitment of the controller to adopt internal processes and policies that ensure comprehensive compliance with rules and good practices related to the protection of personal data; b) is applicable to the entire set of personal data under its control, regardless of how it was collected; c) be adapted to the structure, scale and volume of its operations, as well as the sensitivity of the data processed; d) establish adequate policies and safeguards based on a systematic assessment process of impacts and risks to regardless of how the collection was carried out; c) be adapted to the structure, scale and volume of its operations, as well as the sensitivity of the data processed; d) establish adequate policies and safeguards based on a systematic assessment process of impacts and risks to regardless of how the collection was carried out; c) be adapted to the structure, scale and volume of its operations, as well as the sensitivity of the data processed; d) establish adequate policies and safeguards based on a systematic assessment process of impacts and risks to privacy; e) has the objective of establishing a relationship of trust with the holder, through transparent action and which ensures mechanisms for the holder's participation; f) is integrated into its overall governance structure and establishes and applies internal and external oversight mechanisms; g) have incident response and remediation plans in place; eh) be constantly updated with	LGPD, 2018 chap VII, section II, article 50,*152.1*

#	theme	category	aspect	nature	element(s)	ref
					based on information obtained from continuous monitoring and periodic evaluations; II demonstrate the effectiveness of its privacy governance program when appropriate and, in particular, at the request of the national authority or other entity responsible for promoting compliance with good practices or codes of conduct, which, independently, promote compliance with this Law. § 3 The rules of good practices and governance must be published and updated periodically and may be recognized and disclosed by the authority national.	
115	legal aspects and ethical	security of information	information systems of health	safety requirements, standards of	Art. 49. The systems used for the processing of personal data must be structured in such a way as to featuremeet good practices and governance and the general principles set forth in this Law and other regulatory standards.	LGPD, 2018 chap VII, section I, article 49, *151.1*
116	legal aspects and ethical	telehealth service	georeferencing	indicator	Location of patients in care	ICT-health, 2021 Online services line offered to the patient and telehealth, *833.1*
117	legal aspects and ethical	transfer international of data	contracts	feature	Art. 35. The definition of the content of standard contractual clauses, as well as the verification of specific contractual clauses for a given transfer, global corporate standards or seals, certificates and codes of conduct, referred to in item II of the caput of art. . 33 of this Law, will be carried out by the national authority. § 1 In order to verify the provisions of the caput of this article, the minimum requirements, conditions and guarantees for the transfer that observe the rights, guarantees and principles of this Law must be considered. § 2 In the analysis of contractual clauses, documents or global corporate standards submitted for approval by the national authority, may be required supplementary information or measures taken to verification of processing operations, where necessary. § 3 The national authority may designate certification bodies to carry out the provisions of the caput of this article, which will remain under its supervision under the terms defined in the regulation. § 4 The acts carried out by a certification body may be reviewed by the national authority and, in case of non-compliance with this Law, submitted to review or annulled. § 5 The sufficient guarantees of observance of the general principles of protection and the holder's rights referred to in the caput of this article will also be analyzed in accordance with the technical and organizational measures adopted by the operator, in accordance with the provisions of §§ 1 and 2 of art. . 46 of this Law.	LGPD, 2018 cap V, art 35, *140.1*
118	legal aspects and ethical	transfer international of data	management	holder referred to in item II of	Art. 36. Changes in the guarantees presented as sufficient for the observance of the general principles of featureprotection and rights of the art. 33 of this Law must be communicated to the authority national.	LGPD, 2018 chapter V, art 36, *141.1*
119	legal aspects and ethical	data processing by the government	public companies and economist	companies and	Art. 24. Public companies and government-controlled companies that operate on a competitive basis, subject to the provisions of art. 173 of the Federal Constitution, will have the same treatment given to private legal entities, under the terms featureof this Law. Sole Paragraph. Public government-controlled companies, when operationalizing public policies and within the scope of their execution, will have the same treatment given to bodies and entities of the Public Power, under the terms of this Chapter.	LGPD, 2018 chapter IV, art 24, *132.1*
120	legal aspects and ethical	data processing by the government	purpose of data processing	feature	Art. 23. Processing of personal data by people public law legal entities referred to in the sole paragraph of art. 1 of Law No. 12,527, of November 18, 2011 (Access to Information Law), must be carried out to serve its public purpose, in pursuit of the public interest, with the aim of executing legal powers or fulfilling legal attributions of the public service, provided that: I are informed of the hypotheses in which, in the exercise of their competences, they carry out the processing of personal data, providing	LGPD, 2018 chapter IV, art 23, *131.1*

#	theme	category	aspect	nature	element(s)	ref
					clear and up-to-date information on the legal provision, purpose, procedures and practices used to carry out these activities, in easily accessible vehicles, preferably on their websites; III - a person in charge is appointed when processing personal data, pursuant to art. 39 of this Law; and § 1 The national authority may rule on the forms of publicity for treatment operations. § 2 The provisions of this Law do not exempt the legal entities mentioned in the caput of this article from establishing the authorities referred to in Law No. 12,527, of November 18, 2011 (Access to Information Law). § 3 The deadlines and procedures for exercising the holder's rights before the Government shall observe the provisions of specific legislation, in particular the provisions contained in Law No. of January 29, 1999 General Law of Administrative Procedure), and Law No. 12,527, of November 18, 2011 Law on Access to Information). public administration, in view of the purposes referred to in the caput of this article.	
121	legal aspects and ethical	data processing by the government	request data processing	about the	Art. 29. The national authority may request, at any time, public bodies and entities to carry out data processing operations. featurepersonal data, specific information scope and nature of the data and other details of the treatment carried out and may issue a complementary technical opinion to ensure compliance with this Law.	LGPD, 2018 chapter IV, art 29, *136.1*
122	legal aspects and ethical	data processing data deletion		body, guaranteed, whenever possible, the	Art. 16. Personal data will be eliminated after the end of its processing, within the scope and within the technical limits of activities, with conservation authorized for the following purposes: I compliance with a legal or regulatory obligation by the controller; II study by featureresearch anonymization of personal data; III transfer to a third party, provided that the data processing requirements set forth in this Law are respected; or IV exclusive use by the controller, access by a third party is forbidden, and provided that the data is anonymized.	LGPD, 2018 chapter II, section IV, article 16,*124.1*
123	legal aspects and ethical	data processing	end of treatment of the data	communication	Art. 15. The end of the processing of personal data will occur in the following hypotheses: I verification that the purpose has been achieved or that the data is no longer necessary or relevant to achieve the specific purpose sought; II end of period featuretreatment; III from the holder, including the exercise of your right to revoke consent as provided for in § 5 of art. 8 of this Law, safeguarding the public interest; or IV determination of the national authority, when there is a violation of the provisions of this Law.	LGPD, 2018 chapter II, section IV, article 15,*123.1*
124	legal aspects and ethical	data processing data use		feature	Art. 10. The legitimate interest of the controller only may justify the processing of personal data for legitimate purposes, considered based on concrete situations, which include, but are not limited to: I support and promotion of the controller's activities; and II protection, in relation to the holder, of the regular exercise of his rights or provision of services that the benefit, respecting his legitimate expectations and fundamental rights and freedoms, under the terms of this Law. § 1 When the treatment is based on the legitimate interest of the controller, only personal data strictly necessary for the intended purpose may be processed. § 2 The controller must adopt measures to ensure the transparency of data processing based on its legitimate interest. § 3 The national authority may ask the controller for a report on the impact on the protection of personal data, when the treatment is based on its legitimate interest, observing commercial and industrial secrets. § 4 The communication or shared use between controllers of sensitive personal data relating to health with the aim of obtaining	LGPD, 2018 cap II, section I, art 10,*118.1*

#	theme	category	aspect	nature	element(s)	ref
					economic advantage, except in cases relating to the provision of health services, pharmaceutical assistance and health care, provided that paragraph 5 of this article is observed, including auxiliary diagnosis and therapy services, for the benefit of the interests of data subjects, and to allow: I data portability when requested by the data subject; or	
125	legal aspects and ethical	data processing	anonymization of sensitive data sensitive	feature	Art. 12. Anonymized data will not be considered personal data for the purposes of this Law, except when the anonymization process to which they were submitted is reversed, using exclusively their own means, or when, with reasonable efforts, it can be reversed. § 1 The determination of what is reasonable must take into account objective factors, such as the cost and time needed to reverse the anonymization process, according to the technologies available, and the exclusive use of own means. § 2 For the purposes of this Law, those used to form the behavioral profile of a certain natural person, if identified, may also be considered as personal data. § 3 The national authority may rule on standards and techniques used in anonymization processes and carry out checks on their security, after consulting the National Council for the Protection of Personal Data.	LGPD, 2018 cap II, section II, art 12,*120.1*
126	citizenship and development sustainable	political aspects	sustainability	feature	There is planning by the team to ensure the sustainability of the service.<br>Answer: yes / no / not applicable / don't know	HAOC1, 2019 7,*265.1*
127	citizenship and development sustainable	regulatory aspects	commitment constitutional of face-to-face assistance	face assistance	Art. 19. Distance medical services can never replace the constitutional commitment to guarantee featureface-to- according to the SUS principles of comprehensiveness, equity, universality to all patients.	CFM2314, 2022 art 19,*1857.1*
128	citizenship and development sustainable	assessment	sustainability and continuity	feature	In order for the municipality, the Federal District or the state to continue participating and receiving resources from the Requalification Program, the UBS must inform the Ministry of Health of: a) the start of activities to implement the schedule approved in the project; b) progress, completion of actions, bimonthly output of activities; and c) other information and documents required by the UBS Requalification Program Monitoring System<br><br>Note: Origin: p2554, 2011 art 23, $4	MSPC6, 2017 title II, cap II, section VII, art 145, $4,*357.1*
129	citizenship and development sustainable	citizenship, sustainability and economy of knowledge	digital accessibility	feature	There are digital health services or products that promote digital accessibility. Digital accessibility: Eliminating barriers on the web. The concept presupposes that websites and portals are designed so that all people can perceive, understand, navigate and interact effectively with the pages.	BDHI, 2021 21,*471.1*
130	citizenship and development sustainable	citizenship, sustainability and economy of knowledge	citizenship	feature	There are digital health services or products that promote citizenship. a) Citizenship: It is based on collective rights and a vision of citizenship that is not restricted to the sum of the rights of individuals. It incorporates the promotion of participation and social control and the inseparability between the provision of services and its assertion as a right of individuals and society. It must seek to meet the needs and demands of citizens<br><br>individually, linked to the principles of universality, equality before the law and equity in the provision of services and information.	BDHI, 2021 21,*469.1*
131	citizenship and development sustainable	citizenship, sustainability and economy of knowledge	economy of knowledge	machines. This one	Digital health in the knowledge economy: There are digital health services or products focused on economic transformation or impact on society. Note: Knowledge economy is the use of knowledge (knowledge, know-how) to generate tangible and intangible value. Technology, and in particular knowledge technology, helps to incorporate part of featureof human knowledge in knowledge can be used by decision support systems in various fields to generate economic value. Knowledge economies are also possible without technology. Human capital can be treated as a commercial product (educational and innovative intellectual products and services) in search of a high-value return and a productive asset.	BDHI, 2021 23,*473.1*

#	theme	category	aspect	nature	element(s)	ref
132	citizenship and development sustainable	citizenship, sustainability and economy of knowledge	digital inclusion	feature	There are digital health services or products that promote digital inclusion. Digital inclusion: Acting through digital inclusion so that citizens exercise their effective political participation in the society of the knowledge. Initiatives in this area aim to ensure the dissemination and use of information and communication technologies aimed at social, economic, political, cultural, environmental and technological development, centered on people, especially on excluded communities and segments.	BDHI, 2021 21,*470.1*
133	citizenship and development sustainable	citizenship, sustainability and economy of knowledge	objectives of development sustainable 3 ODS3	feature	Digital health to achieve the sustainable development goals 3 SDG3 There are services or products in digital health that promote the objectives of SDG3. Note: Sustainable development goals 3 have their main focus on population health by 2030, with the 13 goals.	BDHI, 2021 22,*472.1*
134	citizenship and development sustainable	governance and management of information and technology of health information	digital inclusion and reduction of social inequality	feature	From the regulation process of IT and Telecom companies that wish to work in health, the definition of social responsibility requirements, such as, for example, companies that encourage digital inclusion and the reduction of social inequality.	ABRASCO, 2020 1st dimension, 4,*503.1*
135	citizenship and development sustainable	human Resources	equity at work	feature	By 2030, it is considered as a goal to achieve full and productive employment and decent work in all women and men, including for young people and people with disabilities, and equal pay for work of equal value.	UN2030, 2016 , *1823.1*
136	citizenship and development sustainable	human Resources	inclusion	indicator	Number of female professionals who collaborate with the telehealth center.	UN2030, 2016 , *1826.1*
137	citizenship and development sustainable	human Resources	inclusion	feature	The telehealth center has a quota policy for race and ethnicity in their hiring of collaborators.	UN2030, 2016 , *1827.1*
138	citizenship and development sustainable	human Resources	inclusion	indicator	Number of LGBTIQA people in the telehealth center team.	UN2030, 2016 , *1828.1*
139	citizenship and development sustainable	human Resources	inclusion	indicator	The telehealth center has people with PwD disabilities among its collaborators.	UN2030, 2016 , *1829.1*
140	structure and management	characteristics of quality	documentation	feature	The healthcare organization maintains a quality management system for telehealth services that includes: a) a description of quality characteristics suitable for each service offered; b) a description of the procedures that will be used to monitor the achievement and achievement of the quality characteristics	ISO13131, 2021 5.2.2,*103.1*
141	structure and management	knowledge skilled	change management	feature	There is a change management program.	ARGMNMM, 2020 V.111,*651.1*
142	structure and management	knowledge skilled	data governance	feature	There is data governance in public health.	ARGMNMM, 2020 V.114,*654.1*
143	structure and management	team consultations	service delivery	feature	Organization includes staff in consultations about changes in workflow, workloads, required skills, training, or other changes that a telehealth service may require	ISO13131, 2021 9.1.3,*90.1*
144	structure and management	schedule of execution	difficulties	feature	Describe difficulties and barriers to performing a specific activity.	DESD, 2021 , *1779.1*
145	structure and management	definition	telemedicine	feature	Telemedicine should contribute to favoring the doctor-patient relationship. Medicine, when exercised using safe technological and digital means, should aim at the benefit and the best results for the patient, the physician should assess whether telemedicine is the most appropriate method for the patient's needs in that situation. Telemedicine does not replace face-to-face care.	CFM2314, 2022 considerations, *1831.1*
146	structure and management	demographic	roof	indicator	Percentage of locations (municipalities, housing units, populations) that have telemedicine service, outside the locations originally proposed in the program. Measures the degree of progress in implementation. Formula: Number of locations served by telemedicine x 100/ Number of locations that make up the program's target population. Frequency: Half-yearly. Indicator Type. Demographic. Notes: It is necessary to identify the	PAHO, 2016 , *782.1*

#	theme	category	aspect	nature	element(s)	ref
					which is considered a telemedicine service. Additional information can be obtained, such as the population that would potentially benefit from the program.	
147	structure and management	description of services	documentation	feature	The health organization defines in a quality management system: a) the scope, context and purposes of the services it offers, how these purposes can be achieved and how to assess the extent to which the objectives are achieved, and b) the actors who will be responsible by any part of the services, including other health organizations, support organizations, manufacturers, suppliers and other health actors such as care recipients, caregivers and informal carers;	ISO13131, 2021 5.3.2,*104.1*
148	structure and management	Domains	telehealth service domains	health record,	a) domain of people (health workers, patients, managers, user communities); b) domain of machines (equipment, software, information and communication technology); c) domain featurematerials (data, electronic exams, systems); d) domain of methods (work protocols, guidelines, regulation, change management); e) domain of money (costs, payments, reimbursements, financial sustainability);	TMSMM, 2013 4, 5,*432.1*
149	structure and management	endogenous	roof	indicator		PAHO, 2016 , *196.1*
150	structure and management	strategy and investment	strategy plan investment	feature	National digital health strategy or framework: The country have a digital health strategy or framework with an investment plan.	BDHI, 2021 3,*441.1*
151	structure and management	structure	areas in process or technologies	feature	a) Patient care Call Center, Reception, Waiting, Concierge, Hospitality); b) Service and hospitalization units Emergency Room, Hospitalization Units, ICU, Surgical Center, Ambulatory); c) SADT Diagnostic imaging, Laboratory, Endoscopy, Hemodynamics, Hemodialysis); d) HR support services, Quality, CCIH, Continuing Education, Legal, Marketing, Research and Teaching, SAME, SESMT, Occupational Medicine, Nutrition and Dietetics Service); e) Commercial and relationship with Operators; f) Asset security, Maintenance, Works and Clinical Engineering; g) Supply chain Supplies, Purchasing Planning, Warehouse, Pharmacy); h) Information Technology; i) Multiprofessional Team Nursing, Physiotherapy, Speech Therapy, Psychology, Occupational Therapist and Clinical Nutrition); j) Clinical Staff Management of the Clinical Staff, Medical Registration, Medical Records Commission, Fees and medical transfer); k) Administrative management and Corporate Governance; l) Financial Sector Controllership, Costs, Surgical Scheduling, Authorization, Medical Bills, Billing Cycle,	DMIH, 2021 , *485.1*
152	structure and management	structure	core classification	feature	a) state; b) municipal; c) intercity; Note: Origin: p2554, 2011 art 8, $	MSPC5, 2017 title IV, cap I, section I, subsection V, art 471, $,*349.1*
153	structure and management	structure	competences of core	feature	a) organize, offer and regulate the second opinion training, teleconsulting and telediagnosis, in addition to other actions and services offered; b) establish flows, deadlines and protocols, based on the best scientific evidence available, and assignment of primary care teams in order to facilitate the use of services and requests from teams; c) have a team of teleconsultants that guarantees, at a minimum, the offer of teleconsultation and formative second opinion; d) articulate Telehealth with the regulation of the offer of services; e) promote the development, in a shared manner and agreed with the points of care in the network, of flowcharts and clinical protocols that include prior request for a second formative opinion and/or teleconsultation before forwarding or requesting to the regulation center; and f) monitor and evaluate the project, teleconsultations, the response time for users, the number and quality of referrals and requests for tests performed	MSPC5, 2017 title IV, cap I, section I, subsection V, art 471,*348.1*

#	theme	category	aspect	nature	element(s)	ref
					and the expansion of the resoluteness of primary care.<br>Note: Origin: p2554, 2011 art 8	
154	structure and management	structure	knowledge skilled	feature	Specialized knowledge: additional knowledge that can benefit the effective implementation of services of telehealth.	ARGMNMM, 2020 1.4.1, 538.1
155	structure and management	structure	constitution	feature	a) technical-scientific telehealth center; b) telehealth point;<br>Note: Origin: p2546, 2011 cap I, art 3	MSPC5, 2017 title IV, cap I, section I, subsection I, art 449, 336.1
156	structure and management	structure	constitution	feature	This Ordinance establishes, within the scope of the UBS Basic Health Units Requalification Program, the Brasil Redes Informatization and Telehealth Component in Primary Care, integrated to the Brasil Redes National Telehealth Program.<br>Note: Origin: p2554, 2011 art 1	MSPC5, 2017 title IV, cap I, section I, subsection V, art 464, 344.1
157	structure and management	structure	strategy and governance	feature	a) The institution has committees for IT governance and/or digital health, with discussion of priorities, impact analysis and feedback for employees. b) There is a formal digital transformation strategy for the institution. c) There is a structured program for monitoring the patient's health through digital tools. Example: Apps for chronic disease management, educational website, monitoring remote, wellness programs with wearables. d) there is a strategy for process innovation and the incorporation of new technologies. e) The institution has a presence strategy on social networks and digital marketing. f) There is in fact the support of the Board and/or Council for the digital transformation. g) Is there a structured and dedicated budget for the digital transformation with continued investments in innovation.	DMIH, 2021 , 490.1
158	structure and management	structure	networking	feature	Telehealth Brazil Networks in Primary Care will be structured in the form of a network of services that provides for the creation of intermunicipal projects or health regions, managed in a shared manner, with the possibility of participation of the Secretariats State Health, educational institutions and services health, which guarantee the use of technology as a tool for expanding primary care actions and to improve access and quality of health care for citizens<br>Note: Origin: p2554, 2011 art 4	MSPC5, 2017 title IV, cap I, section I, subsection V, art 467, 346.1
159	structure and management	structure	goal	feature	Telehealth Brazil Networks in Primary Care aims to develop actions to support health care and permanent education of primary care teams, aiming at education for work, from the perspective of improving the quality of care, the expansion of the scope of actions offered by these teams, changing care practices and organizing the work process, by offering teleconsulting, second formative opinion and telediagnosis<br>Note: Origin: p2554, 2011 art 3	MSPC5, 2017 title IV, cap I, section I, subsection V, art 466, 345.1
160	structure and management	structure	telehealth points in implantation	indicator	Description: number of health units with points in the implementation of telehealth in each municipality; Numerator: number of points in implantation; Unit: points; Source: municipal data; Period: data must be updated monthly, however, every 6 months (March and September) a general update of the registration database of all centers must be carried out for general cleaning of the database, in relation to the points of teams and registered people .<br>Note: Source: Telehealth Manual for Primary Care, MinSaúde, UFRGS 2012, adapted by the MinSaúde technical team.	MSNT5, 2014 annex I, frame 1, 372.1
161	structure and management	structure	telehealth points implanted	indicator	Description: number of health units with implanted telehealth points in each municipality; Numerator: number of points implanted; Unit: points; Source: municipal data; Period: data must be updated monthly, however, every 6 months (March and September) a general update of the registration database of all centers must be carried out for general cleaning of the database, in relation to the points of teams and registered people .<br>Note: Source: Telehealth Manual for Attention	MSNT5, 2014 annex I, frame 1, 373.1

#	theme	category	aspect	nature	element(s)	ref
					Basic, MinSaúde, UFRGS 2012, adapted by the MinSaúde technical team.	
162	structure and management	structure	preparation organizational	feature	Organizational preparation: fundamental bases that identify topics that need to be established to offer telehealth services.	ARGMNMM, 2020 1.4.1,533.1
163	structure and management	structure	professionals	indicator	Description: number of professionals registered in each municipality and in each professional category; Numerator: number of professionals; Unit: professionals; Source: municipal data; Period: data must be updated monthly, however, every 6 months (March and September) a general update of the registration database of all centers must be carried out for general cleaning of the database, in relation to the points of teams and registered people .<br><br>Note: Source: Telehealth Manual for Primary Care, MinSaúde, UFRGS 2012, adapted by the MinSaúde technical team.	MSNT5, 2014 annex I, frame 1,375.1
164	structure and management	execution	beginning of teleconsultations	indicator	Implementation of teleconsulting	DESD, 2021 , 1814.1
165	structure and management	training and training of human Resources	activities for establish the informatics in health as a profession and as an area of R&D	feature	a) attract public and private organizations to define strategies and approaches to regulate Digital Health as a research area; b) structure instruments of mobilization and persuasion to lead discussions about the relevance for the national public interest; c) promote the<br><br>recognition of health informatics as a profession at the CBO, which includes defining professional profiles and detailing their attributions, duties and ethical limits.	ESD28, 2020 5.2.1,724.1
166	structure and management	training and training of human Resources	activities for promote capacity building of IT professionals		a) bring together and articulate the diverse and relevant actors that develop professional training processes for Health Informatics; b) raise and describe competencies, experiences, knowledge and skills associated with each functional profile necessary for IT professionals to be active featureDigital Health Strategy participants; w)<br><br>identify the volume of professionals to be trained, by profile, in order to measure the demand for education and training; d) prepare and execute a Training Plan to achieve the proposed objectives, including undergraduate, specialization, master's and doctoral efforts.	ESD28, 2020 5.1.2,723.1
167	structure and management	training and training of human Resources	activities for promote capacity building of professionals and health managers		a) bring together and articulate the diverse and relevant actors that develop efforts to train professionals in Health Informatics; b) survey and describe competences, experiences, knowledge and skills associated with each functional profile necessary for health professionals and managers to featurebe active participants in the Health Strategy<br>Digital; c) identify the volume of professionals to be trained, by profile, in order to measure the demand for education and training; d) prepare and execute a Training Plan to achieve the proposed objectives, including undergraduate, specialization, master's and doctoral efforts.	ESD28, 2020 5.1.1,722.1
168	structure and management	training and training of human Resources	priorities	feature	5. Training and qualification of human resources 5.1 Training in health informatics 5.1.1 Promoting the training of health professionals and managers 5.1.2 Promoting training for IT professionals 5.2 Valuing human capital in digital health 5.2.1 Health informatics as a profession and R&D area	ESD28, 2020 5,721.1
169	structure and management	management	logic model	figure	Figure 1. Logical model of the National Telehealth Program Brasil Redes	HAOC2, 2019 5,286.1
170	structure and management	management	logic model	feature	a) inputs: physical and technological structure, political framework conceptual, human resources; b) strategies: distance support; support for permanent education, support for the management and efficiency of public spending; c) offers: telediagnosis, teleconsulting, formative second opinion, tele-education, compulsory flow to the Regulation Centers; d) primary results: improved and timely diagnoses, improved clinical conduct, evidence-based clinical practices, up-to-date professionals and teams, better articulation between AB and specialties; e) secondary results: qualified therapies, increased team resolution potential, reduced queues for exams and specialties;	HAOC3, 2019 6.1,310.1

#	theme	category	aspect	nature	element(s)	ref
					f) impact: increased quality of life for users, rationalized public spending;	
171	structure and management	process management	user regulation	feature	Regulation method for users of the telehealth center	DESD, 2021 , *1783.1*
172	structure and management	management and operation	competences of core		It is incumbent upon the nucleus: a) to be responsible for offering teleconsultation, telediagnosis and formative second opinion; b) compose and maintain a team of teleconsultants and clinical staff of reference specialists, compatible with the demand for the services described in the previous item; c) promote and support the training of teleconsultants under the TBR; e) update the information and insert data in the national TBR information system, with the Ministry of Health, and must present an annual activity report that proves the achievement of the goals set forth in the Work Plan; f) ensure compliance with the interoperability standards proposed by the TBR; g) support the development of protocols that include the featureprior request for teleconsultations on procedures, to assess the need for referral or request to the Emergency Medical Regulation Center; h) monitor and evaluate the TBR within its scope of action, including the analysis of the number of requests for teleconsultations, the response time for service users, the number and pertinence of referrals and requests for complementary exams, with a view to expanding access to services and the improvement of the resolvability of health care for SUS users; and i) develop tele-education actions, based on identified loco-regional needs and in consonance with the priorities of the national health policy. Note: Origin: p2546, 2011 cap II, art 10	MSPC5, 2017 title IV, cap I, section I, subsection II, art 456,*341.1*
173	structure and management	management and operation	national coordination and state	Federal District	a) National Coordination, exercised by the Ministry of Health through the Secretariat for Management of Work and Education in Health SGTES/MS and the Secretariat for Health Care SAS/MS; b) State Coordination, exercised by the Secretary of Health of the State or of the feature or by another body that is part of the state management committee, as approved by the CIB Bipartite Intermanagers Commission; c) State Management Committee; d) Technical-Scientific Center for Telehealth; and e) municipal health manager. Note: Origin: p2546, 2011 cap II, art 6	MSPC5, 2017 title IV, cap I, section I, subsection II, art 452,*339.1*
174	structure and management	management and operation	work plan	feature	§3° The State Management Committee will prepare the Project for the implementation of Telessaúde Brasil Redes in the respective State, sending it to the National Coordination of the Program. § 4 The Project referred to in the previous paragraph will contain a Work Plan agreed upon in the CIB and an Annual Operating Plan, the latter containing the following information: I -definition of the physical goals of the units, assistance and diagnostic and therapeutic support services, with the respective quantitative and flows; II definition of quality goals and evaluation parameters and indicators; and III description of the improvement activities and improvement of the management. § 5° The National Coordination will carry out the analysis and necessary technical readjustments of the Project, in accordance with the national guidelines of Telessaúde Brasil Redes and with budget availability. Note: Origin: p2546, 2011 cap II, art 9	MSPC5, 2017 title IV, cap I, section I, subsection II, art 455,*340.1*
175	structure and management	governance and management of information and technology of health information	multiplicity of instances coordinators	feature	Structuring a new model of information and technology management, of a democratic nature, with social participation and control, which incorporates the current trend of convergence and circulation of knowledge and practices, respecting epistemic, historical and cultural specificities, and which favors the creation of virtuous synergies of political management of the different interests present in this process.	ABRASCO, 2020 1st dimension, 7, *506.1*
176	structure and management	governance and quality management of information standards and technology of health information		feature	Standards, normalizations and certifications of SIS and digital health must be defined within the framework of a decision-making mechanism that integrates information governance and information technology in health, taking into account	ABRASCO, 2020 1st dimension, 13,*512.1*

#	theme	category	aspect	nature	element(s)	ref
					by reference the SUS governance framework management collegiate bodies in the three spheres of government and social control.	
177	structure and management	governance and management of information and technology of health information	quality of systems	feature	a) maturation to support decisions where time and space are variables that impact people's lives; b) critical mission of uninterrupted operation, integrated with other information systems that change continuously; c) the demands for wide access, in a continental country with a deficit of telecommunications, are combined with ease of use that guarantees the identification of the individual, the professional and the health establishment in time and space, with security; d) reliable transactional capability on any platform, from embedded to highly distributed systems; e) criteria for authenticity and maintenance of the chain of custody of records in and between health information systems.	ABRASCO, 2020 1st dimension, 10,*509.1*
178	structure and management	governance and leadership for ESD	activities of consolidation of formal instruments of the ESD	feature	a) review of existing public policies related to Digital Health in Brazil; b) analysis of public policies on Digital Health adopted in countries with health systems similar to Brazil's; c) identification of best practices for the Elaboration and publication of Digital Health policies; d) analysis of the international scenario of the relationship between Digital Health policies and strategies; e) proposition of the scope of the revision of the PNIIS; f) submission of national policy approval processes; g) publication.	ESD28, 2020 1.1.1,*698.1*
179	structure and management	governance and leadership for ESD	activities for establish and implement the ESD governance	feature	a) identification of governance needs for the expansion of Conecta SUS; b) identification of actors to build and validate the proposed model; c) identification of needs, expectations and requirements for collaboration; d) analysis of collaboration and innovation governance models in Digital Health; e) identification of the process of institutionalization of the proposed governance model; f) publication and implementation of the ESD governance model.	ESD28, 2020 1.2.1,*699.1*
180	structure and management	governance and leadership for ESD	action plan	feature	1. Ensure that the ESD28 is developed under the leadership of the Ministry of Health but that, at the same time, be able to incorporate the active contribution of external actors participating in collaboration platforms.	ESD28, 2020 1,*696.1*
181	structure and management	governance and leadership for ESD	priorities	feature	1. Governance and leadership for the ESD 1.1 Institutionalization of the ESD 1.1.1 Consolidation of the formal instruments of the ESD 1.2 Leadership and governance of the ESD 1.2.1 Establish and implement the governance model of the ESD 1.3 Legislation and SD regulation 1.3.1 Define and develop LGPD initiatives 1.3.2 Establish regulation of innovation and interconnectivity environments 1.4 ESD funding 1.4.1 Access public funding sources 1.4.2 Establish mechanisms for private funding	ESD28, 2020 1,*697.1*
182	structure and management	governance and resources organizational	digital strategy	feature	Digital Strategy: There is a formal digital transformation strategy for the institution.	IMDS, 2021 , *688.1*
183	structure and management	governance and resources organizational	health governance digital	feature	Governance: The institution has committees for IT governance and/or digital health, with discussion of priorities, impact analysis and feedback for employees.	IMDS, 2021 , *687.1*
184	structure and management	governance and resources organizational	health program digital	feature	Digital Health Program: There is a program structured to monitor the health of the patient through digital tools.	IMDS, 2021 , *689.1*
185	structure and management	identification of needs in health	target population definition	feature	The target population (health professionals and/ or patients) is defined. Answer: yes / no / not applicable / don't know	HAOC1, 2019 1,*216.1*
186	structure and management	identification of needs in health	definition of health needs	feature	The health needs met by the telehealth service are defined. It is essential that it is directed at responding to health needs that have magnitude, transcendence and/ or vulnerability. Answer: yes / no / not applicable / don't know	HAOC1, 2019 1,*215.1*
187	structure and management	implantation	stage of development of core	indicator	a) intervention not implemented: 033.3%; b) partially implemented intervention: 33.3%66.6%; c) intervention	HAOC3, 2019 frame 2,*295.1*

#	theme	category	aspect	nature	element(s)	ref
					with advanced implantation: 66.6%100%; Answer: 0100%	
188	structure and management	implantation	stage of development of core		Implementation analysis followed as reference the analytical matrix proposed by Najam 1995, called 5Cs. This proposal considers five variables featureinterconnected, seen as determinants of the process implementation of public policies, namely: 1 content; 2 context; 3 appointment; 4 capacity; and 5 clients (or beneficiaries) and coalitions.	HAOC3, 2019 section 2,*296.1*
189	structure and management	implantation	implantation period	indicator	start year and end year	HAOC3, 2019 table 1,*297.1*
190	structure and management	indicators	indicators of structure	indicator	a) number of devices available per capita; b) installed capacity and expected demand; c) clinical qualification of the telehealth team; d) opening hours;<br><br>Note: Not included in the questionnaire. Origin: Donabedian, 1988	HAOC1, 2019 6,*256.1*
191	structure and management	computerization of the 3 attention levels	activities for perform connectivity expansion (internet)		a) strategy alignment with states and municipalities; b) establishment of an operational flow featureand communication; c) identification of obstacles, alternatives and workarounds; d) attracting and evaluating the qualification of potential suppliers.	ESD28, 2020 2.1.1,*706.1*
192	structure and management	computerization of the 3 attention levels	activities for expand the computerization of primary attention	feature	Articulation with CONASS and CONASEMS to agree on the roles and responsibilities of the Ministry of Health and the State and Municipal Health Secretariats in the implementation, maintenance and continuous evolution of computerization and qualification of health data in the federated entities.	ESD28, 2020 2.1.2,*707.1*
193	structure and management	computerization of the 3 attention levels	activities for expand the computerization of other levels of attention		a) articulation with the Ministry of Education and University Hospitals, CONASS, CONASEMS and the local management of health establishments through computerization; b) availability of a Management System for health establishments that can meet the needs of Specialized and Hospital Care; c) establishment of a governance model featureof the adopted system and the way of financing the maintenance activities; d) agreement on roles and responsibilities in the implementation, maintenance and continuous evolution of computerization, as well as in the qualification of data collected and shared; e) establishment of minimum quality standards and permanent assessment of the level of digital maturity of public or private establishments.	ESD28, 2020 2.1.3,*708.1*
194	structure and management	leadership and governance	global coordination	feature	Digital health prioritized at the national level through dedicated bodies and mechanisms for its governance: The country has a specific department, agency or working group for digital health.	BDHI, 2021 1,*438.1*
195	structure and management	leadership and governance	gender balance	feature	Digital health prioritized at the national level through dedicated bodies and mechanisms for its governance: The governance structure and its working groups technician emphasize a balance of the genders of its members.	BDHI, 2021 1a,*439.1*
196	structure and management	leadership and governance	planning strategic	feature	Digital health prioritized nationally through strategic planning: Digital health is included and budgeted in the national health system or in a relevant national strategy or plan.	BDHI, 2021 two,*440.1*
197	structure and management	planning	contracting third-party services	indicator	Hiring of Third-Party Services Legal Entity	DESD, 2021 , *1813.1*
198	structure and management	planning	signed contract	indicator	Signature of the contract with the Intervening Party	DESD, 2021 , *1808.1*
199	structure and management	planning	pent-up demand	indicator	Repressed demand in the municipalities	DESD, 2021 , *1804.1*
200	structure and management	planning	disclosure	feature	How the project is publicized	DESD, 2021 , *1805.1*
201	structure and management	planning	action planning	feature	Develop a document detailing the actions to be carried out, such as team selection, hiring, implementation details, points for improvement, identification of service points	DESD, 2021 , *1778.1*
202	structure and management	planning	communication plan	feature	Existence of the Project Communication Plan	DESD, 2021 , *1802.1*
203	structure and management	planning	work plan	indicator	Elaboration and validation of the work plan with the Department of Digital Health of the Ministry of Health	DESD, 2021 , *1809.1*

#	theme	category	aspect	nature	element(s)	ref
204	structure and management	planning	transfer from knowledge	feature	how the strategy for transferring knowledge and its sustainability is being developed within the State and Municipalities at the end of the Project	DESD, 2021 , *1803.1*
205	structure and management	plan of monitoring and M&A valuation of digital health	activities for formalize and consolidate the instances of M&A governance Connect SUS	feature	a) identification of the actors of each instance; b) elaboration of attribution and competence documents; c) publication of the corresponding ordinances.	ESD28, 2020 M&A, 1.1.1,*746.1*
206	structure and management	preparation organizational	specialized support	feature	There is support from an institution specialized in telehealth services.	ARGMNMM, 2020 I.32,*574.1*
207	structure and management	preparation organizational	coordination	feature	A staff person is assigned to act as the person in charge of telehealth services.	ARGMNMM, 2020 I.22,*564.1*
208	structure and management	preparation organizational	institutional decision	feature	The institution is determined to offer telehealth services.	ARGMNMM, 2020 I.1,*539.1*
209	structure and management	preparation organizational	data governance	feature	The institution establishes governance mechanisms for health data and information.	ARGMNMM, 2020 I.41,*583.1*
210	structure and management	preparation organizational	telehealth program	feature	The institution has a telehealth program in operation.	ARGMNMM, 2020 I.9,*546.1*
211	structure and management	process	organizational chart of telehealth	feature	An organizational chart has been defined for the telehealth area.	ARGMNMM, 2020 I.45,*587.1*
212	structure and management	financial resources	TBR calculation basis at AB	indicator	a) eSF number; b) population coverage; c) number of basic health units in the UF;	MSPC6, 2017 title II, cap II, section VII, art 149,*359.1*
213	structure and management	financial resources	conditions for calculation of the variable component of cost to the federative entity	feature	a) definition and agreement on lines of care and/or priority specialties, involving managers, services and teams participating in the core; b) the definition and agreement of Protocols of Routing and Teleconsulting linked to regulation;	MSPC6, 2017 title II, cap II, section VII, art 157,*364.1*
214	structure and management	financial resources	fundable items on TBRnaAB	feature	a) purchase or lease of equipment and software; b) payment of personnel, pursuant to Consolidation Ordinance No. 6; c) production of materials; d) funding services; e) guarantee of connectivity; f) implementation of a telehealth center; g) other funding expenses related to the objectives of the Program and indicated in the Project.	MSPC6, 2017 title II, cap II, section VII, art 146,*358.1*
215	structure and management	human Resources	jobs and career plan	feature	Career Maturity of Digital Health Professionals in the Public Sector: There are roles and a career path for specific digital health professionals in the public sector.	BDHI, 2021 13,*453.1*
216	structure and management	human Resources	jobs and career path for women	feature	There are positions and a career plan for professionals digital health in the public sector with a focus explicit in (female) health workers.	BDHI, 2021 13th,*454.1*
217	structure and management	security and quality of technology	clinical evidence	feature	The relationship between the care recipient, the healthcare organization and any healthcare supporting organization should be defined in an SLA service level agreement that includes the supporting clinical evidence	ISO13131, 2021 13.1.2,*19.1*
218	structure and management	telehealth service	coverage of establishments	indicator	number of establishments with telehealth service	ICT-health, 2021 Online services line offered to the patient and telehealth, *829.1*
219	structure and management	telehealth service	coverage of remote monitoring	indicator	number of establishments with remote monitoring of patients	ICT-health, 2021 Online services line offered to the patient and telehealth, *830.1*
220	structure and management	telehealth service	coverage of telediagnosis	indicator	number of establishments with telediagnosis service	ICT-health, 2021 Online services line offered

#	theme	category	aspect	nature	element(s)	ref
						to the patient and telehealth, *831.1*
221	structure and management	telehealth service	education coverage and research	indicator	number of establishments operating in education and research	ICT-health, 2021 Online services line offered to the patient and telehealth, *832.1*
222	structure and management	telehealth service	means of access	indicator	Means of communication used in the telehealth service	ICT-health, 2021 Online services line offered to the patient and telehealth, *838.1*
223	structure and management	telehealth service	type of service	indicator	Type of service used	ICT-health, 2021 Online services line offered to the patient and telehealth, *834.1*
224	structure and management	support for the improvement of health care	activities for develop and expand telehealth and digital services in care flow in SUS	feature	a) identify the various SUS actors and managers, aiming to attract them to identify priorities, roles, responsibilities, expected results and goals for population health; b) identify data and Information and Communication Technologies in Health TICS essential for remote care to be integrated into continuous care, at different levels of complexity within the scope of the SUS, with a focus on tackling inequalities in access and use of services of health in the SUS.	ESD28, 2020 3.3.1,*714.1*
225	structure and management	support for the improvement of health care	activities to ensure support for health care networks (reference and counter-reference)		a) structuring of monitoring mechanisms and analysis of the potential use of the RNDS in the evaluation of Health Care Networks; b) integration of information to optimize multidisciplinary care as a fundamental component of comprehensive care; c) seek, together with the Regulation, the use featureadequate health resources, driven by data, both in reference actions and in counter-reference, with information on conduct and clinical history that enable continuity of care and greater resolution of care; d) identify data and technology support needs to achieve the priority.	ESD28, 2020 3.2.1,*712.1*
226	structure and management	support for the improvement of health care	activities to support population health management	expected results	a) identify the different actors and attract them to the identification of priorities, roles, responsibilities, feature and goals for public health; B) identify the data and technological support necessary for the success of the action.	ESD28, 2020 3.2.2,*713.1*
227	structure and management	support for the improvement of health care	activities to support lines of care	feature	a) articulation with municipal and state managers, as well as providers and payers of health services, industry and universities to align roles and responsibilities; b) detailing the scope of ambitions, limits, potentialities and challenges in the review of lines of care and in the construction of safe and guaranteed assistance flows to the citizen; c) identification of the need for data and technological support to support priority lines of care.	ESD28, 2020 3.1.1,*711.1*
228	structure and management	support for the improvement of health care	action plan	MS and also	3. Have the RNDS support best clinical practice through services such as featuretelehealth, and apps developed in other applications that are developed by the collaboration platform.	ESD28, 2020 3,*709.1*
229	structure and management	support for the improvement of health care	priorities	continuity of care at all levels	3. Support for improving health care 3.2 Health promotion and disease prevention 3.2.1 Ensure support for RAS (referral and counter-referral) 3.2.2 Provide support for population health management 3.1 featureSupport for 3.1.1 Support lines of care 3.3 Promotion of telehealth and digital services 3.3.1 Integration of telehealth and digital services into the care flow	ESD28, 2020 3,*710.1*

#	theme	category	aspect	nature	element(s)	ref
230	structure and management	technology service support	management of changes	of telehealth service using	The healthcare organization ensures that procedures are in place to maintain continuity and featurereliability change management to manage necessary changes to services	ISO13131, 2021 13.1.3,*25.1*
231	structure and management	technology service support	management of problems	of telehealth service using	The healthcare organization ensures that procedures are in place to maintain continuity and featurereliability problem management to identify and resolve issues whose cause is unknown	ISO13131, 2021 13.1.3,*24.1*
232	structure and management	technical support from technology	technical assistance	for featuretelehealth services that include provision of	The healthcare organization provides technical support specialized technical advice	ISO13131, 2021 13.1.8,*52.1*
233	structure and management	technical support from technology	documentation	for featuretelehealth services that include management of	The healthcare organization provides technical support documentation	ISO13131, 2021 13.1.8,*53.1*
234	management financial and budgetary	assessment	maintenance cost from the telehealth center	indicator	a) tickets and per diem (expenses with displacements); b) natural person (human resources); c) legal entity (services); d) consumables (maintenance, replacement, application and office hours); e) monthly cost of equipment (total cost of equipment divided by its useful life);	HAOC4, 2019 table 24,*317.1*
235	management financial and budgetary	assessment	maintenance cost from the telehealth center	indicator	a) permanent staff: wages and payments for work; b) physical installations and infrastructure (property rent, depreciation rate, public services: energy, telecommunications and internet, water and sewage; cleaning material and snacks, office material and office supplies); c) other items related to service maintenance (management system software licenses, accounting service, employee and analyst training, taxes and mandatory licenses, permits, authorizations);	HAOC5, 2020 4.1,*320.1*
236	management financial and budgetary	short review term	patient economy	indicator	Measure financial impact for the patient	PAHO, 2016 , *157.1*
237	management financial and budgetary	long evaluation term	economy	indicator	average savings compared to previous years	PAHO, 2016 , *170.1*
238	management financial and budgetary	budget data	actions and expenses per year	table	Table 11. Telediagnosis, teleconsulting, teleeducation actions and total expenditure in reais, by year and period, Telehealth Centers (maintenance), Ministry of Health, Brazil(2016 2018. Year, 2016, 2017, 2018, Period 20162018	HAOC2, 2019 4.1,*279.1*
239	management financial and budgetary	budget data	budget data	table	Table 4. Budget data, contractual term, forecasts and amount paid in Reais TED and Agreements via FNS by Telehealth Nucleus (maintenance), Brazil 2016 2018 Nucleus, Beginning of Term, End of Term, Months of Execution, Granting Value, Paid Amount , Counterpart, Object, Monthly Value (average)]	HAOC2, 2019 4.1,*272.1*
240	management financial and budgetary	budget data	average monthly spend	table	Table 5. Consolidated average monthly expenditure on Telehealth Centers (maintenance), Ministry of Health, Brazil 2016-2018. Date, Department, University, Total]	HAOC2, 2019 4.1,*273.1*
241	management financial and budgetary	budget data	average monthly spend	table	Table 7. Average monthly expenditure in Reais, by month and Telehealth Center (maintenance), Ministry of Health, Brazil 2016. Date, 117, Total]	HAOC2, 2019 4.1,*275.1*
242	management financial and budgetary	budget data	average monthly spend	table	Table 8. Average monthly expenditure in Reais, by month and Telehealth Center (maintenance), Ministry of Health, Brazil 2017. Date, 117, Total]	HAOC2, 2019 4.1,*276.1*
243	management financial and budgetary	budget data	average monthly spend	table	Table 9. Average monthly expenditure in reais, by month and Telehealth Center (maintenance), Ministry of Health, Brazil 2018. Date, 117, Total]	HAOC2, 2019 4.1,*277.1*
244	management financial and budgetary	budget data	average monthly spend dermato/electro	table	Table 6. Consolidated average monthly expenditure on Telehealth Centers (maintenance) including national offers of telediagnosis in dermatology and electrocardiology, Brazil 2016-2018. Date, Department, University, Total]	HAOC2, 2019 4.1,*274.1*
245	management financial and budgetary	budget data	spent per year	table	Table 10. Expenditure in reais, per year and Telehealth Center (maintenance), Ministry of Health, Brazil 2016 2018. Date, 117, Total]	HAOC2, 2019 4.1,*278.1*
246	management financial and	expenses of receivers of	detailing financial	feature	The health organization clearly informs the recipient of care, through procedures, on the	ISO13131, 2021 11.1.5,*65.1*

#	Om youbreedThemental	wu TheitdIt isThegdOOlaughsThe	aspect	nature	dleetion alpes tof(is n) funds for the provision of health care health to be provided	ref
247	management financial and budgetary	expenses of receivers of care	refund policy	that may be claimed by other	The health care organization clearly informs the care recipient using responsible procedures of the arrangements for reimbursing patient costs. featurecare organizations, such as insurance companies, that can reimburse the costs of providing health care	ISO13131, 2021 11.1.5,*66.1*
248	management financial and budgetary	expenses of receivers of care	refund policy	feature	Procedures for making reimbursement with coverage of additional telehealth services	ICT-health, 2021 Use of technologies of information and from the communication in the area of health: a telehealth in 2021,*843.1*
249	management financial and budgetary	service delivery of technology	management financial	feature	The healthcare organization ensures that procedures are in place to maintain financial arrangements that account for the costs and charges for users of telehealth services	ISO13131, 2021 13.1.4,*28.1*
250	management financial and budgetary	strategy and investment	public funding	indicator	Public funding for digital health: What is the estimated % of annual public health spending going to digital health.	BDHI, 2021 4,*442.1*
251	management financial and budgetary	structure	support in the implementation of the e-SUSAB strategy	feature	The financial incentive for funding is established for municipalities, states and the Federal District with TBR telehealth centers to support the implementation of the e-SUS AB strategy.	MSPC5, 2017 title IV, cap I, section I, subsection VII, art 493,*351.1*
252	management financial and budgetary	financial execution	physical percentage of execution of a activity in the period object total	indicator	Indication or calculation of the percentage of execution of the activity based on the total execution over the expected total execution in the total period of the object.	DESD, 2021 , *1782.1*
253	management financial and budgetary	financial execution	total value of executing an activity for the total period of the object	indicator	Description or total value of execution of the activity for the total period of the object. (examples: 20 hours, 2 events, 150 participants, 2 teleeducation activities, 160 teleconsultations/month indicating the total in the period, 40 telehealth points to be included in the period, 2 SOF/ month indicating the total in the period	DESD, 2021 , *1781.1*
254	management financial and budgetary	financial execution	total estimated value of execution of a activity for the total period of the object	indicator	Value in Reais of the expected total execution of the activity for the total period of the object.	DESD, 2021 , *1780.1*
255	management financial and budgetary	exogenous	cost	indicator	cost of care	PAHO, 2016 , *202.1*
256	management financial and budgetary	financial	cost effectiveness	indicator	Cost effectiveness of the project	DESD, 2021 , *1807.1*
257	management financial and budgetary	financial	monthly savings per County	indicator	monthly savings by municipality	DESD, 2021 , *1797.1*
258	management financial and budgetary	financial	cost reduction	indicator	The savings generated from avoiding the referral of patients in the network, in the qualification of test requests, in the reduction of the response time between the realization and issuance of the report, in the possibility of clinical discussion between the requesting physician and the specialist teleconsultant	DESD, 2021 , *1791.1*
259	management financial and budgetary	financial	return of investment	indicator	Return on investment made	DESD, 2021 , *1796.1*
260	management financial and budgetary	financial	payback time	indicator	payback time	DESD, 2021 , *1798.1*
261	management financial and budgetary	assistance funds of health	communication	feature	The healthcare organization regularly informs healthcare professionals about reimbursements, funding, and charges for telehealth services	ISO13131, 2021 7.1.3,*96.1*
262	management financial and budgetary	governance and management of information and	innovation promotion policies	feature	Innovation promotion policies must be clear and accessible to everyone. It is recommended to encourage monitoring and inspection mechanisms in order to	ABRASCO, 2020

#	theme	category	aspect	nature	element(s)	ref
		technology of health information			tracking possible transfers of funds from the underfunded SUS budget to market expansion initiatives by IT and Telecom companies.	1st dimension, two,501.1
263	management financial and budgetary	governance and management of information and technology of health information	promotion priorities	feature	SUS management instances, together with social control (health councils and conferences) must deepen inter-federal articulation and between other sectors of public administration to define promotion priorities that meet health demands.	ABRASCO, 2020 1st dimension, two,500.1
264	management financial and budgetary	governance and leadership for ESD	activities to access the sources of public funding	aspects; b) plan	a) identify possible sources of public financing, in the three spheres of government, by profile and lines of financing and ways of obtaining resources, among featureother the approach and prepare the documentation for obtaining these resources, mobilizing partners and sponsors, respecting current legislation.	ESD28, 2020 1.4.1,702.1
265	management financial and budgetary	governance and leadership for ESD	activities for to establish mechanisms for the private financing	feature	a) identify legal and adequate private funding mechanisms for the ESD28, in particular funding for the Collaboration Space; b) identify sources of private funding suited to the objectives of the ESD28, by profile and line of business<br><br>financing, as well as ways of obtaining these resources, respecting the specific legislation; c) plan the approach and prepare the legal documentation to obtain these resources, mobilizing partners and sponsors.	ESD28, 2020 1.4.2,703.1
266	management financial and budgetary	governance and resources organizational	financial resources	featurefor digital	Investment: There is a structured and dedicated budget transformation with investments continued in innovation.	IMDS, 2021 , 693.1
267	management financial and budgetary	indicators	indicators of economic result	indicator	a) cost per capita/procedure/diagnosis with equal effectiveness (cost-minimization); b) impact on access or quality at the same similar cost (cost-utility);<br><br>Note: Not included in the questionnaire. Origin: Donabedian, 1988	HAOC1, 2019 6,259.1
268	management financial and budgetary	preparation organizational	financing in telehealth	feature	The institution has specific funding for telehealth.	ARGMNMM, 2020 I.17,554.1
269	management financial and budgetary	financial resources	conditions for funding component monthly core fixed naAB	indicator	The fixed component will correspond to 50% (fifty percent) of the total value of the funding financial incentive to be passed on to the respective federative entity and will be defined according to the size of the telehealth center. To receive the fixed component of the incentive, the following requirements will be considered: a) have at least 80 (eighty) participating Primary Care teams registered on the telehealth platform; b) have teams linked to UBS with a telehealth point in SCNES; c) have teams with a history of requesting teleconsultations in the last 3 (three) months; d) send, on a monthly basis, the Nucleus' production report to the current Telehealth Monitoring System;	MSPC6, 2017 title II, cap II, section VII, art 153,360.1
270	management financial and budgetary	financial resources	conditions for funding component monthly core fixed naAB	indicator	The fixed component will correspond to 50% (fifty percent) of the total value of the funding financial incentive to be passed on to the respective federative entity and will be defined according to the size of the telehealth center. To receive the fixed component of the incentive, the following requirements will be considered: a) have at least 80 (eighty) participating Primary Care teams registered on the telehealth platform; b) have teams linked to UBS with a telehealth point in SCNES; c) have teams with a history of requesting teleconsultations in the last 3 (three) months; d) send, on a monthly basis, the Nucleus' production report to the current Telehealth Monitoring System;<br><br>Note: It appears in the Consolidation Ordinance no. 6 of 2017.	MSNTCUST, 2015 2.1,426.1
271	management financial and budgetary	financial resources	conditions for costing component monthly indicator core variable in AB		The variable component will correspond to the remaining 50% (fifty percent) of the total value of the funding financial incentive to be passed on to the respective federative entity. To receive this component, the following dimensions and indicators will be monitored: 1 1st Dimension: activity of active and participating teams, related to professionals who use telehealth services in the reference month: This dimension is equivalent to 40% of the total value	MSNTCUST, 2015 2.2,427.1

#	theme	category	aspect	nature	element(s)	ref
					of the variable incentive. It aims to monitor the scope of use of teams and professionals each month. 1a) number of active teams: list of active teams and the total number of teams participating in the nucleus; 1b) number of active physicians: list of active physicians and the total number of physicians participating in the nucleus; 2 2nd Dimension: definition and agreement on lines of care and/or priority specialties: this dimension of the costing component is equivalent to 20% of the total value of the variable incentive. It aims to encourage the analysis and qualification of referrals to Specialized Care in the SUS with a focus on Lines of Care and/or priority specialties. 2a) The definition and agreement of lines of care and/or priority specialties, in the collegiate instances of the SUS, involving managers, services and teams participating in the telehealth center; 3 The definition and agreement of Access Regulation Protocols or Routing Protocols articulated to regulation; 3rd Dimension: Total production of synchronous and asynchronous teleconsultations, per team each month. This dimension is equivalent to 40% of the total value of the variable funding incentive. Aims to monitor the production of<br><br>NTS teleconsultations. 3a) Teleconsultation on general topics, if any professional on the team performs at least 01 teleconsultation in the reference month, with the exception of the one related to teleconsultation by a physician in the priority CL. 3b) Teleconsultation related to the priority LC care line, if the team physician performs at least 01 teleconsultation related to the Care Line or specialty defined and agreed upon, in the reference month. Note: Production monitoring will take into account: - Synchronous teleconsultations: response via web conference or telephone 0800 644 6543, registered on the platform. - teleconsultations<br>asynchronous: reply in text, after a maximum of 72 hours.<br>Note: It appears in the Consolidation Ordinance no. 6 of 2017.	
272	management financial and budgetary	systems and services, patterns and interoperability	billing health additional	feature	Supplementary Health Billing Cycle): The entire billing cycle (order to cash) is digital with tools that facilitate scheduling and authorisation, as well as analyzing and sending bills.	IMDS, 2021 , *669.1*
273	management financial and budgetary	systems and services, patterns and interoperability	billingSUS	feature	SUS Billing Cycle The entire billing cycle (order to cash) is digital with tools that facilitate scheduling and authorization, as well as the analysis and sending of bills.	IMDS, 2021 , *667.1*
274	management financial and budgetary	sustainability financial	planning	feature	The organization has developed a business model and implemented a financial plan that considers the costs, benefits, accessibility and sustainability of telehealth services	ISO13131, 2021 7.1.2,*95.1*
275	infrastructure and technology	service level agreements SLA	documentation	feature	The healthcare organization has included in the service level agreements the following: a) the desired quality characteristics, quality objectives and quality procedures required to ensure the quality objectives of the telehealth service; b) the<br>service quality characteristics, including non-medical aspects such as privacy; c) responsibilities and obligations; d) documentation and audit processes; e) financial management arrangements.	ISO13131, 2021 8.1.5,*94.1*
276	infrastructure and technology	agents of data processing	interoperability	feature	Art. 40. The national authority may rule on interoperability standards for portability purposes, free access to data and security, as well as on the retention time of records, especially with a view to necessity and transparency.	LGPD, 2018 cap VI, art 40, *145.1*
277	infrastructure and technology	environment of interconnectivity	national data architecture	feature	Establishment of interoperability standards and protocols between different systems and health devices with the RNDS, preferably open, for the exchange of information that allows the unique identification of individuals and the evolution of their electronic health record.	PNIIS, 2021 chapter II, section VI, art 9, II, *804.1*
278	infrastructure and technology	environment of interconnectivity	activities for develop standards for health information	feature	a) establish the governance of standards of interoperability, in harmony with the policies and criteria established throughout the development of Priority 1; b) establish the set of<br>functionalities to be attended to each cycle of	ESD28, 2020 6.2.2,*734.1*

#	theme	category	aspect	nature	element(s)	ref
					development of the RNDS; c) establish, exercise, validate and disseminate requirements, norms, criteria for use, models and standards adopted to meet the expected functionalities.	
279	infrastructure and technology	environment of interconnectivity	activities for strengthen the terminology repository of RTS health	feature	a) establish terminology needs for each RNDS development cycle; b) establish the governance of the terminologies adopted for each RNDS development cycle; c) exercise, validate and publish the implemented terminology services, as well as the adopted governance model.	ESD28, 2020 6.2.1,*733.1*
280	infrastructure and technology	environment of interconnectivity	activities for deploy services outpatient regulation		a) develop mechanisms, practices and experiences of syntactic, semantic, operational and organizational interoperability so that information systems used by appointment scheduling services, exams and outpatient procedures interoperate with each other, not only within the public sector, but also with health private and supplementary; b) explore aspects of featureagreement and organizational interoperability and technology that make Regulation viable as the great strategic tool for optimizing public and private assistance resources and productivity, which is its vocation; c) experiment and evaluate models for sharing outpatient resources among all actors who so desire, public or private, and also between public and private.	ESD28, 2020 6.1.6,*732.1*
281	infrastructure and technology	environment of interconnectivity	activities for promote the interoperability with primary care		a) attract software and service companies, public health and supplementary health organizations, with experience and knowledge in Information Systems for Primary Care; b) establish, featureexercise and publish standards for describing, exchanging, storage and access to health information, with a priority focus on Primary Care; c) systematically evaluate the results obtained and disseminate them to produce knowledge and attract new actors.	ESD28, 2020 6.1.1,*727.1*
282	infrastructure and technology	environment of interconnectivity	activities for promote the interoperability with laboratories	feature	a) establish the set of essential laboratory tests for Primary Health Care, including Health Surveillance; b) establish, exercise, validate and publish norms, criteria for use, models and standards that will allow the interoperability between these systems for the purposes of laboratory tests; c) evaluate the results obtained at each cycle and establish the expansion plan.	ESD28, 2020 6.1.2,*728.1*
283	infrastructure and technology	environment of interconnectivity	activities for promote the interoperability with telehealth services	feature	a) attract public and private actors; b) define minimum information content for telehealth; c) identify specific patterns for telehealth; d) define quality criteria for telehealth services and systems; e) define criteria for the regulation of telehealth care; f) implement and test models of telehealth systems and services; g) widely disseminate lessons learned.	ESD28, 2020 6.1.5,*731.1*
284	infrastructure and technology	environment of interconnectivity	activities for promote the interoperability between levels of care		a) establish the essential data flow needs between the levels of care, according to the perspective of each of the relevant actors, from the featureuser of health services to paying sources; B) establish, exercise, validate and publish norms, criteria for use, models and standards for interoperability between levels of care.	ESD28, 2020 6.1.3,*729.1*
285	infrastructure and technology	environment of interconnectivity	activities for promote the interoperability between pharmacy services		a) identify and attract relevant actors; b) identify the essential functionalities and their requirements for featureeach stage of the project cycle; c) establish, exercise, validate and disseminate the models, standards, norms and results obtained in each project cycle.	ESD28, 2020 6.1.4,*730.1*
286	infrastructure and technology	environment of interconnectivity	action plan		6. Allow the National Health Data Network to enhance collaborative work at all levels featurehealth sectors so that technologies, concepts, standards, service models, policies and regulations are put in place.	ESD28, 2020 6,*725.1*
287	infrastructure and technology	environment of interconnectivity	priorities		feature6. Interconnectivity Environment 6.1 Interoperability with external systems 6.1.1 Promote interoperability with primary care 6.1.2 Promote interoperability with laboratories 6.1.3 Promote interoperability across levels of care 6.1.4 Promote interoperability with pharmacy services 6.1.5 Promote interoperability with healthcare services telehealth 6.1.6 Implement outpatient regulation services 6.2 Standards and terminologies 6.2.1	ESD28, 2020 6,*726.1*

#	theme	category	aspect	nature	element(s)	ref
					Strengthen RTS 6.2.2 Develop standards for health information	
288	infrastructure and technology	digital environment, applications administrative	systems administrative	feature	The administrative management platforms are prepared to accompany the implementation of telehealth services.	ARGMNMM, 2020 III.c.80,*622.1*
289	infrastructure and technology	digital environment, clinical applications	information management	feature	There are standard operating procedures for the management of data and processes related to the telehealth.	ARGMNMM, 2020 III.b.72,*614.1*
290	infrastructure and technology	digital environment, clinical applications	data management	feature	There are standard operating procedures for the management of data and processes related to the patients attention.	ARGMNMM, 2020 III.b.77,*620.1*
291	infrastructure and technology	digital environment, clinical applications	systems integration	feature	The institution considers its telehealth solution to be integrable with other existing systems and processes, such as medical records, patient portals, messaging, etc.	ARGMNMM, 2020 III.b.76,*619.1*
292	infrastructure and technology	digital environment, clinical applications	interoperability	feature	There is interoperability between different systems and databases of telehealth services.	ARGMNMM, 2020 III.b.74,*617.1*
293	infrastructure and technology	digital environment, clinical applications	national platform	feature	The institution has access to the telehealth and distance communication from the Ministry of Country health.	ARGMNMM, 2020 III.b.69,*611.1*
294	infrastructure and technology	digital environment, clinical applications	patient portal	feature	There is a patient portal (web, app, system, etc.).	ARGMNMM, 2020 III.b.70,*612.1*
295	infrastructure and technology	digital environment, clinical applications	traceability of communications	feature	Platforms for medical records used in telehealth services are capable of including copies of all electronic communications related to patients. Note: Item 78 does not appear on the questionnaire.	ARGMNMM, 2020 III.b.79,*621.1*
296	infrastructure and technology	digital environment, clinical applications	patient record	feature	The institution uses an electronic patient record system or electronic clinical history.	ARGMNMM, 2020 III.b.68,*610.1*
297	infrastructure and technology	digital environment, clinical applications	patient safety	feature	There are guidelines on patient safety.	ARGMNMM, 2020 III.b.73a,*615.1*
298	infrastructure and technology	digital environment, clinical applications	data security	feature	There are guidelines on data privacy and confidentiality.	ARGMNMM, 2020 III.b.73b,*616.1*
299	infrastructure and technology	digital environment, clinical applications	software for telehealth	feature	The institution knows the software and solutions for information technology necessary to offer the services of telehealth.	ARGMNMM, 2020 III.b.71,*613.1*
300	infrastructure and technology	digital environment, clinical applications	reference term	feature	The institution has terms of reference for the acquisition of IT solutions.	ARGMNMM, 2020 III.b.75,*618.1*
301	infrastructure and technology	digital environment, internet connection and connectivity	Internet access	feature	There is a fixed and stable internet connection service.	ARGMNMM, 2020 III.a.57,*599.1*
302	infrastructure and technology	digital environment, internet connection and connectivity	internet adequacy	feature	Bandwidth makes it possible to offer telehealth services without affecting other services.	ARGMNMM, 2020 III.a.58,*600.1*
303	infrastructure and technology	digital environment, internet connection and connectivity	technical support for connectivity	feature	The institution has its own technical support to solve problems related to connectivity.	ARGMNMM, 2020 III.a.63,*605.1*
304	infrastructure and technology	digital environment, internet connection and connectivity	internet calculation	feature	The bankroll needed to provide telehealth services has been calculated.	ARGMNMM, 2020 III.a.59,*601.1*
305	infrastructure and technology	digital environment, internet connection and connectivity	cybersecurity	feature	There is a cybersecurity plan.	ARGMNMM, 2020 III.a.64,*606.1*
306	infrastructure and technology	digital environment, internet connection and connectivity	minimum connection Internet	feature	The institution has a minimum connection of 1Mbps upload and 1Mbps download dedicated to providing services of telehealth.	ARGMNMM, 2020 III.a.60,*602.1*
307	infrastructure and technology	digital environment, internet connection and connectivity	contingency of infrastructure	feature	There is a contingency plan for equipment and connectivity failures.	ARGMNMM, 2020 III.a.66,*608.1*
308	infrastructure and technology	digital environment, internet connection and connectivity	minimum equipment	feature	Does the institution have a laptop or computer desktop, with camera, speakers and microphone, dedicated to providing teleconsultation services.	ARGMNMM, 2020 III.a.61,*603.1*
309	infrastructure and technology	digital environment, internet connection and	scalability of connectivity	feature	The impacts of the implementation of new services of telehealth on the current technological infrastructure are	ARGMNMM, 2020

#	theme	category	aspect	nature	element(s)	ref
		connectivity			considered.	III.a.67,609.1
310	infrastructure and technology	digital environment, internet connection and connectivity	budget for equipment	feature	There is a budget needed to purchase equipment for teleconsultation.	ARGMNMM, 2020 III.a.62,604.1
311	infrastructure and technology	digital environment, internet connection and connectivity	support of connectivity	feature	There is access to guides and support manuals for troubleshooting connectivity issues.	ARGMNMM, 2020 III.a.65,607.1
312	infrastructure and technology	digital environment, equipment	technical support	feature	Technical support is available from specialists in information technology.	ARGMNMM, 2020 III.d.85,627.1
313	infrastructure and technology	digital environment, equipment	management of equipment	feature	There is a secure place to store equipment when not in use.	ARGMNMM, 2020 III.d.82,624.1
314	infrastructure and technology	digital environment, equipment	management of absolescence	feature	There is a program for renewing and/or acquiring technical equipment for telehealth services.	ARGMNMM, 2020 III.d.86,628.1
315	infrastructure and technology	digital environment, equipment	infrastructure	feature	The technological capability of storage and security necessary for document and record virtual encounters.	ARGMNMM, 2020 III.d.84,626.1
316	infrastructure and technology	digital environment, equipment	inventory of computing	feature	There is an inventory of all equipment technicians, including make, model, time of operation and serial number.	ARGMNMM, 2020 III.d.81,623.1
317	infrastructure and technology	digital environment, equipment	maintenance	feature	There is a maintenance program for technical equipment.	ARGMNMM, 2020 III.d.83,625.1
318	infrastructure and technology	average rating term	technical problems	indicator	number of technical issues per unit	PAHO, 2016 , 166.1
319	infrastructure and technology	average rating term	time for troubleshooting	indicator	time it took to resolve a technical issue per unit	PAHO, 2016 , 167.1
320	infrastructure and technology	confidentiality	interoperability semantics of health records	feature	The organization implements processes that ensure the semantic interoperability of health records used in telehealth services with other health services.	ISO13131, 2021 14.1.4,9.1
321	infrastructure and technology	confidentiality	data protection	feature	The organization implements processes that confirm that information and communication technologies meet minimum standards for protecting health records in electronic storage or transmission	ISO13131, 2021 14.1.4,7.1
322	infrastructure and technology	knowledge skilled	architecture of information	feature	There is an information architecture in public health.	ARGMNMM, 2020 V.112,652.1
323	infrastructure and technology	knowledge skilled	standards	feature	There are health informatics standards and interoperability.	ARGMNMM, 2020 V.110,650.1
324	infrastructure and technology	knowledge skilled	public health	feature	There is knowledge of technological training in public health.	ARGMNMM, 2020 V.113,653.1
325	infrastructure and technology	guidelines for computerization of public and private institutions	federal articulation	feature	Promoting articulation with the Ministry of Science, Technology, Innovations, with the Ministry of Communications, and with federal regulatory agencies, with a view to implementing the infrastructure and procedures necessary for the area of digital health;	PNIIS, 2021 cap II, section II, art 5, III,788.1
326	infrastructure and technology	guidelines for computerization of public and private institutions	minimum set of data	feature	Encouraging the standardization of information models national minima, as well as vocabularies and health terminologies.	PNIIS, 2021 cap II, section II, art 5, Vi,791.1
327	infrastructure and technology	guidelines for computerization of public institutions and private	interoperability with RNDS	feature	Provision of adequate ICT for the RNDS to receive the clinical history throughout the user's life cycle, for continuity of care, through electronic medical records from public and private institutions, in accordance with the legal guidelines on document management, set out in Law No. 8159, of January 8, 1991, and Decree No. 4073 of January 3, 2002;	PNIIS, 2021 cap II, section II, art 5, IV,789.1
328	infrastructure and technology	guidelines for computerization of public and private institutions	mechanisms of security	feature	Strengthening security mechanisms for accessing health systems, data and information, which ensure their availability, authenticity and integrity, encouraging the use of electronic signatures and biometric systems.	PNIIS, 2021 cap II, section II, art 5, V,790.1
329	infrastructure and technology	guidelines for computerization of	minimum standard for infrastructure and ICT security	feature	Induction to computerization with a minimum standard for infrastructure and ICT security to be achieved in order to accelerate the adoption of medical records systems	PNIIS, 2021 cap II, section II, art 5, I,786.1

#	theme	category	aspect	nature	element(s)	ref
		public and private institutions			electronics, decision support and management as an integral part of health services and processes;	
330	infrastructure and technology	guidelines for computerization of public and private institutions	electronic medical record	health complexity, as well as	Encouraging the use of electronic medical record systems with security and functionalities compatible with health work processes and adequate to meet the reality of the different spheres of management and featurelevels of health establishments considering the needs of the public and private sectors, in order to comply with data exchange standards with the RNDS;	PNIIS, 2021 cap II, section II, art 5, II, *787.1*
331	infrastructure and technology	general provisions	codification of establishments CNES	feature	a) 75 telehealth b) 75.1 Technical-Scientific Center of the National Telehealth Program Brasil Redes c) 75.2 Telehealth Unit<br>Note: Origin: p2546, 2011 cap III, art 12	MSPC5, 2017 title IV, cap I, section I, subsection III, art 458, *342.1*
332	infrastructure and technology	general provisions	codification of CNES services	potentials evoked by	services with respective CBOs: a) 160 teleconsulting (asynchronous, synchronous, formative second opinion); b) 107 hearing health care service (diagnosis in audiology/ otology by telemedicine); c) 122 diagnostic services using dynamic graphic methods feature(evoked potentials, telemedicine); d) 131 ophthalmology service (diagnosis in ophthalmology by telemedicine); e) 133 pulmonology service (diagnosis in pulmonology, diagnosis in pulmonology by telemedicine) Note: Origin: p2546, 2011 cap III, art 13	MSPC5, 2017 clause transitory exhausted, no consolidable, *343.1*
333	infrastructure and technology	ecosystem of innovation	national software architecture	featureinclude	Encouraging the establishment and up-to-date maintenance of a national health software repository that publicly accessible components and applications, and unrestricted, compliant with standards and protocols for functionality, interoperability and security	PNIIS, 2021 chapter II, section VII, art 10, II, *809.1*
334	infrastructure and technology	ecosystem of innovation	health regulatory process	and regulation	Improvement of the health regulatory process to support innovation, through the implementation of actions feature mechanisms for the productive complex of digital health, in order to guarantee the security and adequacy of the devices in an agile process.	PNIIS, 2021 chapter II, section VII, art 10, VI, *813.1*
335	infrastructure and technology	endogenous	equipment	indicator	need for equipment and supplies	PAHO, 2016 , *198.1*
336	infrastructure and technology	service delivery of technology	continuity of service	service SLA	The healthcare organization ensures that procedures are in place to maintain patient level agreements. feature specifying service levels necessary to support the agreed continuity of care	ISO13131, 2021 13.1.4, *27.1*
337	infrastructure and technology	service delivery of technology	management of continuity	provide recovery	The healthcare organization ensures that procedures are in place to manage the continuity of featureservice to plans for telehealth services when there is a significant failure	ISO13131, 2021 13.1.4, *31.1*
338	infrastructure and technology	service delivery of technology	management of availability	feature	The healthcare organization ensures procedures are in place to manage availability to ensure systems are reliable and available for use in accordance with SLA service level agreements provided to users of telehealth services	ISO13131, 2021 13.1.4, *30.1*
339	infrastructure and technology	service delivery of technology	management of capacity	infrastructure	The healthcare organization ensures that procedures are in place to manage capacity featureensuring that IT resources are available to effectively meet the planned demand for telehealth services	ISO13131, 2021 13.1.4, *29.1*
340	infrastructure and technology	structure	digital environment	feature	Digital environment: necessary technological infrastructure, including hardware and software.	ARGMNMM, 2020 1.4.1, *535.1*
341	infrastructure and technology	structure	structure and culture	feature	a) The IT team is structured and qualified to support the digital transformation of the institution. b) There is a program for training on digital health for each employee individually. c) There is a culture in the institution that favors and encourages the adoption of digital technologies.	DMIH, 2021 , *489.1*
342	infrastructure and technology	structure	infrastructure	feature	a) Does the institution have an infrastructure that guarantees high availability and system performance. b) Does the institution have policies for information security with regular training and technologies that guarantee the privacy and confidentiality of information	DMIH, 2021 , *487.1*

#	theme	category	aspect	nature	element(s)	ref
					information. c) There is a well-defined technical architecture for the integration between systems and solutions.	
343	infrastructure and technology	structure	means of access by fixed device	indicator	Description: means of accessing the telehealth service through fixed devices; Numerator: number of accesses on the telehealth platform through fixed devices; Unit: registered hits; Source: platform data; Period: data must be updated monthly, however, every 6 months (March and September) a general update of the registration database of all centers must be carried out for general cleaning of the database, in relation to the points of teams and registered people . Note: this indicator can be implemented on existing platforms or taken from metric tools and website analysis such as Google Analytics.<br><br>Note: Source: Telehealth Manual for Primary Care, MinSaúde, UFRGS 2012, adapted by the MinSaúde technical team.	MSNT5, 2014 annex I, frame 1,*378.1*
344	infrastructure and technology	structure	means of access via mobile device	indicator	Description: means of accessing the telehealth service through mobile devices; Numerator: number of accesses to the telehealth platform via mobile devices; Unit: registered hits; Source: platform data; Period: data must be updated monthly, however, every 6 months (March and September) a general update of the registration database of all centers must be carried out for general cleaning of the database, in relation to the points of teams and registered people . Note: this indicator can be implemented on existing platforms or taken from metric tools and website analysis such as Google Analytics.<br><br>Note: Source: Telehealth Manual for Primary Care, MinSaúde, UFRGS 2012, adapted by the MinSaúde technical team.	MSNT5, 2014 annex I, frame 1,*377.1*
345	infrastructure and technology	structure	services and applications	feature	a) Is the clinical documentation available in digital through an electronic medical record system or similar. b) The care team uses systems and technologies in patient care and care. Example: Electronic Patient Record and Electronic Prescription). c) The institution uses a computerized system for prescribing medication, nursing care and requesting exams with features that increase prescription safety. Example: Drug interaction alerts, dose alerts, allergy, altered laboratory test alerts, etc. d) The institution uses a bedside technology that automates the 5 rights in the administration of medications and other substances. Example: uses a trolley or PDA to scan the bar code of the patient's wristband and the medication, allowing the automatic realization of the 5 rights: right patient, right medication, right dose, right time and right route. e) There are clinical decision support systems integrated into the medical record capable of proposing<br><br>diagnoses and procedures. Example: an intelligent form that calculates a score/scale and, right after filling it out, the system suggests a prescription according to the patient's clinical condition. f) Diagnostic modalities (laboratory and imaging tests) are available in digital format through computerized systems, integrated into the medical record and with an exam portal accessible to professionals and patients. g) The supply chain is computerized and automated, from the planning stages to dispensing. h) Medical prescriptions are reviewed by a clinical pharmacist with the support of a computerized system with alerts. Example: Drug interaction alerts, dose alerts, allergy, altered laboratory test alerts, etc. i) The entire billing cycle (order to cash) is digital with tools that facilitate scheduling and authorisation, as well as analyzing and sending bills. j) The monitoring equipment is integrated with the electronic medical record<br><br>advancing to the concept of IoT Internet of Things. Example: multi-parametric monitors, glucometers, anesthesia cart. k) The institution has mobile applications to promote patient engagement and/or facilitate access to information by professionals.	DMIH, 2021 , *486.1*

#	theme	category	aspect	nature	element(s)	ref
					Example: Apps for doctors or for patients. I) The institution has or uses telemedicine services.	
346	infrastructure and technology	management of equipment of technology	service level agreements SLA	feature	The organization ensures that devices used for telehealth services are accompanied by SLA service level agreements that can support the agreed continuity of care.	ISO13131, 2021 13.1.9,*57.1*
347	infrastructure and technology	management of equipment of technology	adequacy		The organization ensures that devices used for telehealth services are usable and suitable for featurethe purpose and are supported by relevant information, including clinical evidence for device effectiveness	ISO13131, 2021 13.1.9,*54.1*
348	infrastructure and technology	management of equipment of technology	communication		The organization ensures that the devices used for feature telehealth services reliably communicate using available telecommunications services	ISO13131, 2021 13.1.9,*56.1*
349	infrastructure and technology	management of equipment of technology	interoperability	feature	The organization ensures that devices used for telehealth services support interoperability using appropriate standards with other telehealth services	ISO13131, 2021 13.1.9,*55.1*
350	infrastructure and technology	management of implantation of technology	availability of technological resources	feature	The healthcare organization ensures that procedures are in place to confirm that equipment or software is available for telehealth services.	ISO13131, 2021 13.1.6,*38.1*
351	infrastructure and technology	management of implantation of technology	management of equipment	feature	The healthcare organization ensures that procedures are in place to install equipment and devices for telehealth services in accordance with the manufacturer's or supplier's guidelines	ISO13131, 2021 13.1.6,*40.1*
352	infrastructure and technology	management of implantation of technology	management of projects		The healthcare organization ensures that procedures are in place to design, build, test, and featuredeploy equipment and devices for telehealth using an appropriate project management methodology	ISO13131, 2021 13.1.6,*37.1*
353	infrastructure and technology	management of implantation of technology	maintenance of resources	feature	The healthcare organization ensures that procedures are in place to repair or replace defective equipment and devices and remove the defective equipment and devices	ISO13131, 2021 13.1.6,*41.1*
354	infrastructure and technology	management of implantation of technology	resource operation		The healthcare organization ensures that the procedures featureare in place to confirm that a piece of equipment or software is safe to operate	ISO13131, 2021 13.1.6,*39.1*
355	infrastructure and technology	management of infrastructure of technology	communication	feature	The healthcare organization ensures that the infrastructure used for telehealth services reliably communicates using telehealth services. telecommunications available	ISO13131, 2021 13.1.5,*35.1*
356	infrastructure and technology	management of infrastructure of technology	financial	feature	The healthcare organization ensures that the infrastructure used for telehealth services is financially efficient throughout the infrastructure lifecycle.	ISO13131, 2021 13.1.5,*36.1*
357	infrastructure and technology	management of infrastructure of technology	infrastructure		The health organization ensures that the infrastructure featureused for telehealth services is usable and fit for purpose	ISO13131, 2021 13.1.5,*34.1*
358	infrastructure and technology	management of infrastructure of technology	interoperability	feature	The healthcare organization ensures that the infrastructure used for telehealth services supports interoperability using appropriate standards with other telehealth services	ISO13131, 2021 13.1.5,*33.1*
359	infrastructure and technology	management of infrastructure of technology	processes of planning		The health organization ensures that the infrastructure used for telehealth services is based on featurea requirements analysis and planning process responsible for covering design, implementation, operation and technical support	ISO13131, 2021 13.1.5,*32.1*
360	infrastructure and technology	operation management of technology	backup and restore	feature	The healthcare organization ensures that ICT operations management can provide day-to-day technical oversight of the ICT infrastructure used for telehealth services, including backup and restoration services	ISO13131, 2021 13.1.7,*43.1*
361	infrastructure and technology	operation management of technology	technical diversity	feature	The healthcare organization ensures that ICT operations management can provide day-to-day technical oversight of the ICT infrastructure used for telehealth services, including technical diversity management	ISO13131, 2021 13.1.7,*48.1*
362	infrastructure and technology	operation management of technology	management of storage		The healthcare organization ensures that ICT operations management can provide technical oversight feature daily use of the ICT infrastructure used for telehealth, including monitoring and storage management	ISO13131, 2021 13.1.7,*46.1*

#	theme	category	aspect	nature	element(s)	ref
363	infrastructure and technology	operation management of technology	systems management	daily use of the	The healthcare organization ensures that ICT operations management can provide technical oversight feature ICT infrastructure used for telehealth, including system monitoring and management	ISO13131, 2021 13.1.7,*45.1*
364	infrastructure and technology	operation management of technology	IT infrastructure	daily use of the	The healthcare organization ensures that ICT operations management can provide technical oversight feature ICT infrastructure used for telehealth, including a secure and stable ICT infrastructure	ISO13131, 2021 13.1.7,*47.1*
365	infrastructure and technology	operation management of technology	network services	daily use of the	The healthcare organization ensures that ICT operations management can provide technical oversight feature ICT infrastructure used for telehealth, including network monitoring and management	ISO13131, 2021 13.1.7,*44.1*
366	infrastructure and technology	operation management of technology	user support	daily use of the	The healthcare organization ensures that ICT operations management can provide technical oversight feature ICT infrastructure used for telehealth, including timely support for all users	ISO13131, 2021 13.1.7,*42.1*
367	infrastructure and technology	governance and management of information and technology of health information	adequacy of standards	necessarily be preceded by consistent	The adoption of any SIS/e-health and digital health norms, standards or granting of certification must feature analysis of the context of its origin in relation to the specific context of its application in the country.	ABRASCO, 2020 1st dimension, 15,*513.1*
368	infrastructure and technology	governance and management of information and technology of health information	document management and evidence	permanent. The production of evidence about the usefulness	The management and governance of health documents and file systems must be subject to qualification feature of information as a management tool should be encouraged.	ABRASCO, 2020 1st dimension, 1, *499.1*
369	infrastructure and technology	governance and management of information and technology of health information	quality of infrastructure of communication	the respective state public companies	Map and coordinate the telecommunications services of the various operators that receive resources from the SUS, in partnership with the secretariats featureof health and and municipal, so that connectivity infrastructure overlaps are identified, stopping the waste of public resources.	ABRASCO, 2020 1st dimension, 12,*511.1*
370	infrastructure and technology	governance and management of information and technology of health information	reduction of fragmentation of information and technology	Interoperability, harmonization of	Actions to be taken to overcome ITIS fragmentation should be in an open format and featurepublic. terminologies and standardization of health information are essential initiatives.	ABRASCO, 2020 1st dimension, 5,*504.1*
371	infrastructure and technology	computerization of the 3 attention levels	action plan	2.1.1 Perform connectivity expansion (internet)	2. Computerization of the 3 levels of care 2.1 Computerization of health facilities in the country feature 2.1.2 Expand the computerization of primary care 2.1.3 Expand the computerization of other levels of care	ESD28, 2020 two,*705.1*
372	infrastructure and technology	computerization of the 3 attention levels	priorities	adoption of electronic medical records systems and	2. Induce the implementation of health systems computerization policies, accelerating the feature hospital management as an integrating part of health services and processes.	ESD28, 2020 two,*704.1*
373	infrastructure and technology	infrastructure	accessibility	feature	The telehealth center has accessible facilities adapted for people with disabilities.	UN2030, 2016 , *1825.1*
374	infrastructure and technology	infrastructure	connectivity	feature	Connection quality for teleconsultations	ICT-health, 2021 Use of technologies of information and from the communication in the area of health: a telehealth in 2021,*846.1*
375	infrastructure and technology	infrastructure	physical structure	indicator	Delivery of the physical structure for service	DESD, 2021 , *1810.1*
376	infrastructure and technology	infrastructure	high infrastructure availability	feature	has an infrastructure that guarantees high High Availability Infrastructure: The institution system availability and performance.	IMDS, 2021 , *680.1*
377	infrastructure and technology	infrastructure	interoperability	architecture feature	Systems and Solutions Architecture: There is an well-defined technique for the integration between different clinical information systems.	IMDS, 2021 , *682.1*

#	theme	category	aspect	nature	element(s)	ref
378	infrastructure and technology	infrastructure	planning and maintenance of infrastructure	feature	Planning and ongoing maintenance of digital health infrastructure: There is an articulated plan in place to support the digital health infrastructure (including equipment such as computers, tablets, phones, supplies, software, devices, etc.) with supply and maintenance.	BDHI, 2021 16,*460.1*
379	infrastructure and technology	infrastructure	planning and maintenance of infrastructure	feature	a) There is an articulated plan to support the communication infrastructure for digital health with delivery and maintenance. b) Is there an articulated plan to support the infrastructure of computing/informatics for digital health with supply and maintenance. c) There is an articulated plan to support the technical and operational support infrastructure for digital health with delivery and maintenance.	BDHI, 2021 16a, 16b, 16c, *461.1*
380	infrastructure and technology	infrastructure	quantity of counties involved/assisted	indicator	number of municipalities involved/served	DESD, 2021 , *1799.1*
381	infrastructure and technology	infrastructure	government rules for web accessibility	feature	The telehealth core website follows the rules of accessibility advocated by the Brazilian government and the W3C.	UN2030, 2016 , *1824.1*
382	infrastructure and technology	infrastructure	technologies used	feature	types of technologies used in care	ICT-health, 2021 Use of technologies of information and from the communication in the area of health: a telehealth in 2021,*849.1*
383	infrastructure and technology	facilities of organizations of health	adequacy of equipment	feature	The organization ensures that the equipment can be used effectively to transmit and receive information of an appropriate quality including audio or video information and can be used securely.	ISO13131, 2021 12.1.2 and 12.1.3, *59.1*
384	infrastructure and technology	facilities of organizations of health	physical space	feature	The organization ensures that there is adequate physical space for holding consultations and an appropriate level of comfort and privacy is available to the health professional, care recipient(s) and informal caregivers	ISO13131, 2021 12.1.2 and 12.1.3, *58.1*
385	infrastructure and technology	the user as protagonist	activities for deploy services personal record of health	feature	a) identify and analyze national and international experiences in the use of Personal Health Record systems, including their ability to interoperate with other systems, the use of accessible standards and technologies, costs and, above all, adherence to use; b) establish technical, functional and usability requirements for the integration of these systems to the RNDS; c) coordinate design, development, validation, implementation and dissemination of the use of Personal Health Record applications, using the best practices identified and documented as part of this action.	ESD28, 2020 4.2.1,*719.1*
386	infrastructure and technology	patterns and interoperability	national data architecture	feature	National digital health architecture and health information exchange: There is a defined national digital health architecture or health information exchange model.	BDHI, 2021 14,*455.1*
387	infrastructure and technology	patterns and interoperability	update of national architecture	feature	The national digital health architecture or model of exchange of health information is updated systematically or periodically.	BDHI, 2021 15th,*457.1*
388	infrastructure and technology	patterns and interoperability	identification	feature	For the crossing of patient identifiers from different information systems, the integration specification IHEPIX Patient Identifier Cross-Referencing will be used).	MSP2073, 2011 annex 2,*112.1*
389	infrastructure and technology	patterns and interoperability	medical images	feature	For the representation of information related to imaging exams, the DICOM standard will be used.	MSP2073, 2011 annex 2,*111.1*
390	infrastructure and technology	patterns and interoperability	interoperability of systems	feature	To establish interoperability between systems, with a view to integrating test results and requests, the HL7 Health Level 7 standard will be used.	MSP2073, 2011 annex 2,*109.1*

#	theme	category	aspect	nature	element(s)	ref
391	infrastructure and technology	patterns and interoperability	information standards in health	feature	a) For the coding of clinical terms and mapping of national and international terminologies in use in the country, aiming to support the semantic interoperability between the systems, the SNOMEDCT terminology will be used. b) For exam coding laboratory, the LOINC Logical standard will be used Observation Identifiers Names and Codes). c) For the encoding of identification data on product labels relating to human blood, cells, tissues and organ products, the ISBT 128 standard will be used.	MSP2073, 2011 annex 2, *110.1*
392	infrastructure and technology	patterns and interoperability	information standards in health	feature	Other classifications that will be used to support the interoperability of health systems: ICD, CIAP2 (Primary Health Care), TUSS and CBHPM (Hierarchical Brazilian Classification of medical procedures) and SUS procedures table.	MSP2073, 2011 annex 2, *113.1*
393	infrastructure and technology	patterns and interoperability	information standards in health	feature	Health information standards: Digital health standards exist for information architecture and processes, device and system interoperability, terminology, and security.	BDHI, 2021 15, *456.1*
394	infrastructure and technology	patterns and interoperability	safety and integrity	characteristic	To guarantee the safety and integrity of information, the WSSecurity standard will be adopted for encryption and digital signature of information	MSP2073, 2011 Annex 1, *108.1*
395	infrastructure and technology	payment of services	payments	feature	The healthcare organization is able to collect payments and refund allowances for payments when telehealth services are not provided free of charge to care recipient	ISO13131, 2021 7.1.4, *97.1*
396	infrastructure and technology	preparation organizational	Internet access	feature	The institution has stable internet access.	ARGMNMM, 2020 I.7, *544.1*
397	infrastructure and technology	preparation organizational	definition of services of telehealth	feature	It is clearly understood what services can be offered through telehealth.	ARGMNMM, 2020 I.2, *540.1*
398	infrastructure and technology	preparation organizational	electricity	feature	There is a steady supply of electricity.	ARGMNMM, 2020 I.21c, *561.1*
399	infrastructure and technology	preparation organizational	national tools	feature	To provide telehealth services, the tools available from the Coordination of Telehealth, from the Ministry of Health.	ARGMNMM, 2020 I.8, *545.1*
400	infrastructure and technology	preparation organizational	lighting	feature	There is acceptable lighting.	ARGMNMM, 2020 I.21d, *562.1*
401	infrastructure and technology	preparation organizational	infrastructure	feature	The institution has the necessary infrastructure to provide telehealth services.	ARGMNMM, 2020 I.21, *558.1*
402	infrastructure and technology	preparation organizational	place for asynchronous telequeries	feature	There is adequate space allocated for asynchronous queries.	ARGMNMM, 2020 I.21b, *560.1*
403	infrastructure and technology	preparation organizational	location for synchronous teleconsultations	feature	There is adequate space allocated for synchronous queries.	ARGMNMM, 2020 I.21a, *559.1*
404	infrastructure and technology	preparation organizational	service requirements of telehealth	feature	It has the necessary requirements to offer telehealth services.	ARGMNMM, 2020 I.4, *541.1*
405	infrastructure and technology	privacy	management of use consent of data	feature	The organization shall implement a system for obtaining and recording contracts provided by recipients of precautions regarding the use of data and information based on the principles of free and informed consent	ISO13131, 2021 14.1.2, *4.1*
406	infrastructure and technology	service quality	telemedicine system	indicator	Stability and reliability of the system and method used to transmit information	PAHO, 2016 , *191.1*
407	infrastructure and technology	data quality	reliability of data	feature	The organization ensures that processes are in place to monitor the reliability of the representation, generation, collection, transmission, exchange and use of data by telehealth services	ISO13131, 2021 14.1.7, *15.1*
408	infrastructure and technology	data quality	availability of Law Suit	feature	The organization ensures that processes are in place to support emergency response activities. effective and safe healthcare when the quality of telehealth service data exceeds control limits	ISO13131, 2021 14.1.7, *16.1*

#	theme	category	aspect	nature	element(s)	ref
409	infrastructure and technology	security of information	standards	of feature	Art. 51. The national authority will encourage the adoption technical standards that facilitate control by holders of your personal data.	LGPD, 2018 chap VII, section II, article 51,153.1
410	infrastructure and technology	security and quality of technology	communication of problems	feature	The relationship between the care recipient, the healthcare organization, and any healthcare supporting organization should be defined in an SLA service level agreement that enables the care recipient to raise issues with the provider about a service, equipment, or device.	ISO13131, 2021 13.1.2,22.1
411	infrastructure and technology	telehealth service	teleconsulting offer infrastructure	feature	Priority should be given to opting for the use of a Telehealth Platform approved by MinSaúde and integrated with SMART.	MSNT50, 2015 4,757.1
412	infrastructure and technology	services and applications	digital identity of establishments of health	feature	Digital identity management of healthcare facilities and facilities for digital health: There are records in healthcare systems with unique identification of healthcare facilities and public (and private if applicable) facilities available, accessible and up-to-date.	BDHI, 2021 19,464.1
413	infrastructure and technology	services and applications	digital identity unambiguous of patients	feature	Specifically, there is a secure digital record of unique patient identification Master Patient Index MPI available, accessible and up-to-date for healthcare purposes.	BDHI, 2021 20th,467.1
414	infrastructure and technology	services and applications	security of digital identity of patients	feature	Digital identity management of individuals for healthcare: Secure digital patient records are available, accessible, and up-to-date for healthcare purposes.	BDHI, 2021 20,466.1
415	infrastructure and technology	services and applications	data security	feature	a) Specifically, is there a secure digital birth record available, accessible and in use for health purposes. b) Specifically, there is a secure digital death record with unique identification of individuals available, accessible and in use for health purposes. c) Specifically, there is a secure digital vaccination record available, accessible and in use for health purposes.	BDHI, 2021 20b, 20c, 20d, 468.1
416	infrastructure and technology	systems and services, patterns and interoperability	clinical decision support	feature	Clinical decision support: There are decision support systems clinic integrated into the medical record capable of proposing diagnoses and procedures.	IMDS, 2021 , 662.1
417	infrastructure and technology	systems and services, patterns and interoperability	supply chain		Supply Chain: The supply chain is featurecomputerized and automated, from the stages of planning to dispensing.	IMDS, 2021 , 665.1
418	infrastructure and technology	systems and services, patterns and interoperability	bedside check	feature	Bedside Check: The facility uses bedside technology that automates the 5 rights in administering medications and other substances. It uses a trolley or PDA, for example, to scan the bar code of the patient's wristband and the medication, allowing the automatic realization of the 5 right: right patient, right drug, right dose, right time and right route.	IMDS, 2021 , 661.1
419	infrastructure and technology	systems and services, patterns and interoperability	information cycle	feature	SUS APS Information Cycle The entire information cycle is digital with tools that automate its processes, as well as the analysis and dispatch of remittances.	IMDS, 2021 , 668.1
420	infrastructure and technology	systems and services, patterns and interoperability	diagnostic imaging	feature	Imaging Diagnosis: Imaging diagnostic modalities are available in digital format through computerized systems and integrated into the electronic medical record.	IMDS, 2021 , 664.1
421	infrastructure and technology	systems and services, patterns and interoperability	clinical documentation	feature	Clinical Documentation: Clinical and administrative documentation is available in digital format through an information system (eg Electronic Patient Record).	IMDS, 2021 , 658.1
422	infrastructure and technology	systems and services, patterns and interoperability	integration of devices	feature	Integration of devices and IoT The monitoring equipment is integrated with the electronic medical record, advancing the concept of IoT Internet of Things.	IMDS, 2021 , 670.1
423	infrastructure and technology	systems and services, patterns and interoperability	interoperability	feature	Connection to Data Sharing Networks: The institution is connected to a network of sharing health data for continuity of care.	IMDS, 2021 , 677.1
424	infrastructure and technology	systems and services, patterns and interoperability	Clinical Laboratory		Clinical Analysis Laboratory: The laboratory of feature clinical analysis of the institution is computerized and without transcription.	IMDS, 2021 , 663.1
425	infrastructure and technology	systems and services, patterns and	mobility	feature	Mobility: The institution's professionals use mobile devices (laptops, tablets, smartphones	IMDS, 2021 , 671.1

#	theme	[illegible]	aspect	nature	[illegible] this facilitated to the information.	ref
426	infrastructure and technology	systems and services, patterns and interoperability	electronic prescription	feature	Electronic Prescription: The institution uses a system computerized for drug prescription, nursing care and ordering exams.	IMDS, 2021 , *660.1*
427	infrastructure and technology	systems and services, patterns and interoperability	registration system electronic health SRES	feature	Art. 3º § 1º Telemedicine care must be registered in physical medical records or in the use of information systems, in the patient's SRES Electronic Health Record System, meeting the standards of representation, terminology and interoperability. Art. 3 § 2 The SRES used must enable the capture, storage, presentation, transmission and printing of digital and identified health information and fully meet the requirements of Security Assurance Level 2 NGS2, in the Brazilian Public Key Infrastructure standard ICPBrasil) or another legally accepted standard. Art. 3º § 5º The SRES must provide interoperability/interchangeability, using flexible protocols, through which two or more systems can communicate effectively and with a guarantee of confidentiality, privacy and data integrity. Art. 3º § 8º When using institutional platforms, when necessary, the attending physician must be guaranteed the right of access to the patient's data, during the entire period of legal validity of its preservation.	CFM2314, 2022 art 3,*1840.1*
428	infrastructure and technology	support for the improvement of health care	ICT implementation	feature	Implementation of information and communication technology solutions that enable the improvement in the organization of the work process in health, the quality of care contact and the digital transformation of health facilities;	PNIIS, 2021 chapter II, section III, article 6, I,*792.1*
429	infrastructure and technology	technical support from technology	software testing	feature	The healthcare organization provides technical support for telehealth services that include proof of concept and pilot tests;	ISO13131, 2021 13.1.8,*51.1*
430	infrastructure and technology	technology	storage of data	feature	Form of storage of the data produced	DESD, 2021 , *1794.1*
431	infrastructure and technology	data processing by the government	interoperability	feature	Art. 25. The data must be kept in an interoperable and structured format for shared use, with a view to the execution of public policies, the provision of public services, the decentralization of public activity and the dissemination and access of information by the general public .	LGPD, 2018 chapter IV, art 25, *133.1*
432	monitoring and assessment	agents of data processing	risk management	feature	Art. 38. The national authority may determine the controller to prepare an impact report on the protection of personal data, including sensitive data, referring to its data processing operations, under the terms of the regulation, observing commercial and industrial secrets. Single paragraph. Observing the provisions of the caput of this article, the report must contain, at least, the description of the types of data collected, the methodology used to collect and guarantee the security of the information and the analysis of the controller with regard to measures, safeguards and adopted risk mitigation mechanisms.	LGPD, 2018 cap VI, art 38, *143.1*
433	monitoring and assessment	risk analysis	review processes	feature	The healthcare organization ensures that it regularly reviews the description, quality planning, service experience, outcomes, risk management of healthcare processes for telehealth services and reviews its quality plans for these services	ISO13131, 2021 6.3.2,*99.1*
434	monitoring and assessment	assessment	monitoring of activities in implementation of e-SUS AB	feature	a) hold web conferences on the e-SUS AB strategy during the term of the work plan; b) hold workshops on the e-SUS AB strategy during the period in which the work plan is in effect; w) carry out visits to Basic Health Units to implementation of the PEC, during the validity period of the work plan; d) monitoring the evolution of the e-SUS AB implementation stage and sending data in the municipalities linked to the work plan;	MSPC5, 2017 title IV, cap I, section I, subsection VII, art 495,*352.1*
435	monitoring and assessment	assessment	evaluation of usefulness from SOF	indicator	Was this SOF helpful to you? a) a lot; b) reasonable; c) little; d) nothing;	HAOC3, 2019 chart 2,*307.1*
436	monitoring and assessment	assessment	economic evaluation of telecardiology	indicator	average value of the variables: a) travel and stay time; b) cost of displacement; c) opportunity cost of the patient; d) opportunity cost of the companion; e) daily cost of the patient; f) daily cost of the companion;	HAOC4, 2019 table 16,*316.1*

#	theme	category	aspect	nature	element(s)	ref
437	monitoring and assessment	assessment	adequacy guidelines and criteria	feature	a) impact; b) feasibility; c) connectivity; d) sustainability; e) cost-effectiveness; f) scope; g) project coverage; h) financial availability; Note: Origin: p2554, 2011 art 19	MSPC5, 2017 title IV, cap I, section I, subsection V, art 482,*350.1*
438	monitoring and assessment	assessment	service effectiveness of telecardiology	indicator	a) avoided referrals: number of patients attended by the service, with normal or borderline classification on the ECG. These patients are referred to secondary care in the conventional model. b) impact on hospitalizations: number and duration of hospitalizations whose main diagnoses coincided with the following diagnoses: [specific ICD list]. c) number, average length of stay and average value of hospitalizations that occurred due to the most frequent condition in the period, that is, ischemic heart disease, in residents of the selected municipalities, whose main diagnosis in the AIH corresponded to the three most frequent groups of diagnoses related to it . The values obtained were compared with the previous period, without the intervention of telecardiology. Note:	HAOC4, 2019 , *314.1*
439	monitoring and assessment	assessment	evaluation period of teleconsultations	indicator	quarterly<br>Note: Origin: p2554, 2011 art 20, $2	MSPC6, 2017 title II, cap II, section VII, art 142, $2,*356.1*
440	monitoring and assessment	assessment	response quality of teleconsulting	indicator	In evaluating the quality of the answer, the structure of the answer was analyzed: objectivity in answering the main question; language used by the teleconsultant; necessary complementation following the principles and attributes of the PHC; promotion of permanent education in health; presentation of basic references to allow access by the applicant. Mandatory items evaluated: a) direct response; b) complementation; c) APS attributes; d) permanent education; e) references. In addition to these five mandatory items, they may also contain a summary of evidence and a description of the search strategy. For each mandatory item of the response protocol, a score ranging from 0 to 10 was assigned. The direct answer had weight 3 (three), the reference weight 1 (one) and the others weight 2 (two). The final grade was the result of the weighted average of the analyzed topics. According to the final grade, the responses were classified according to the Likert Scale as: excellent (score 9.1 to 10, very good (score 8.1 to 9, good 7.1 to 8, regular 6.1 to 7 and bad (less than or equal to 6.	NTSHU UFMA, 2019 4.7,*495.1*
441	monitoring and assessment	risk assessment	Law Suit	feature	The healthcare organization implements a responsible process for risk assessment that: a) follows a documented risk assessment process, as described in ISO 31000, to assess the priority risks to manage, and b) spells out which factors influence the identification of each risk	ISO13131, 2021 6.2.2,*98.1*
442	monitoring and assessment	evaluation and monitoring	documentation	indicators and metrics of	The healthcare organization develops a document that: a) describes how services will be evaluated; b) describes whether services are being evaluated for specific clinical outcomes and/or their contributions to the overall goals of health services; w) featureidentifies the quality assessment to be used for each service; d) describes how assessments from multiple telehealth services will be aggregated; e) describes how the data collection system will enable the evaluation of each service	ISO13131, 2021 5.5.2,*783.1*
443	monitoring and assessment	capabilities	capabilities of the telehealth service	feature	a) non-existent, non-evaluated; b) proposed / experimental (ad hoc / experimental); c) effective; d) reliable (reliable); e) available (available); f) repetitive; g) interoperable (interoperable); h) developed and operated according to standards and norms; i) adherence to the specifications of regulatory bodies; j) consistent (consistent); k) maintained (maintained); l) efficiency (efficiency); m) continuous improvement; n)	TMSMM, 2013 4.2,*433.1*

#	theme	category	aspect	nature	element(s)	ref
					continuously addressed; o) measured (measured); p) monitored;	
444	monitoring and assessment	knowledge skilled	indicators	feature	There are indicators for telehealth projects as a tool to reduce health inequities.	ARGMNMM, 2020 V.108, *648.1*
445	monitoring and assessment	ecosystem of innovation	evaluation of health technologies	feature	Standardization of the methodology for analyzing health technologies in order to accelerate adoption in health establishments, government agencies and private institutions.	PNIIS, 2021 chapter II, section VII, art 10, VIII, *815.1*
446	monitoring and assessment	ecosystem of innovation	periodic evaluation	feature	Encouraging the use of sample surveys and periodic surveys for cases where universal and continuous data collection is not justified, in order to optimize costs and routine work.	PNIIS, 2021 chapter II, section VII, art 10, V, *812.1*
447	monitoring and assessment	effectiveness	attendance of patients	indicator	Number of patients seen after the implementation of the telemedicine program, compared to the number of cases before implementation in a given period	PAHO, 2016 , *187.1*
448	monitoring and assessment	effectiveness	team of experts	indicator	Number of specialists accessible after the implementation of the telemedicine program, compared to the number before the implementation for a given period	PAHO, 2016 , *188.1*
449	monitoring and assessment	effectiveness	inclusion	indicator	Number of people with disabilities or physical, economic limitations, etc. who had access to specialized care through the program	PAHO, 2016 , *189.1*
450	monitoring and assessment	effectiveness	program stopped by technical problems	indicator	Number of hours or days that the program does not provide services due to a technological or operational problem. Interpretation: The indicator should be as low as possible and shows the result of the non-existence of contingency and prevention plans for unexpected events, mainly related to technology. Formula: Total hours that the program is not operating due to technical problems x 100/ total hours of assistance. Frequency: Monthly. Indicator Type. Efficiency. Notes: A service log is required. This indicator may be related to various technical or procedural issues, such as failures in the videoconferencing equipment or the internet network, or lack of a specialized physician due to logistical issues. It complements the list of performed/scheduled appointments.	PAHO, 2016 , *205.1*
451	monitoring and assessment	effectiveness	teleconsultations carried out	indicator	Successful consultations using telemedicine to see patients. Interpretation: Determines the effectiveness of program logistics. Formula: Number of teleconsultations performed x 100/Number of teleconsultations scheduled. Frequency: Monthly. Indicator Type. Efficiency. Notes: What is considered a successful telemedicine consultation must be determined. Indicator related to avoided transfers, as regular consultations would necessarily imply transferring the patient. This indicator can show the reasons for canceled consultations (human, technical, operational, administrative, logistical).	PAHO, 2016 , *206.1*
452	monitoring and assessment	efficiency	access	indicator		PAHO, 2016 , *194.1*
453	monitoring and assessment	efficiency	roof	indicator		PAHO, 2016 , *195.1*
454	monitoring and assessment	efficiency	costs	indicator	cost monitoring	PAHO, 2016 , *192.1*
455	monitoring and assessment	project stage	achievement of goals	indicator	Achieving the proposed, desired and expected objectives and achievements	PAHO, 2016 , *182.1*
456	monitoring and assessment	project stage	technical capacity of users	indicator		PAHO, 2016 , *174.1*
457	monitoring and assessment	project stage	cost benefit	indicator		PAHO, 2016 , *175.1*
458	monitoring and assessment	project stage	efficiency	indicator		PAHO, 2016 , *177.1*
459	monitoring and assessment	project stage	efficiency	indicator		PAHO, 2016 , *176.1*
460	monitoring and assessment	project stage	project feasibility	indicator		PAHO, 2016 , *171.1*

#	theme	category	aspect	nature	element(s)	ref
461	monitoring and assessment	structure	data and information	institution evaluates the impact of health interventions	a) The institution uses tools that allow the collection, analysis and presentation of data for process improvement and information-oriented decision-making (assistance, operation and management). b) The feature digital in its financial, operational and assistance indicators. c) The patient's clinical information is shared with other health institutions for the continuity of care.	DMIH, 2021 , *488.1*
462	monitoring and assessment	structure	team	indicator	Description: number of health teams assisted by telehealth in each municipality; Numerator: number of teams; Unit: teams; Source: municipal data; Period: data must be updated monthly, however, every 6 months (March and September) a general update of the registration database of all centers must be carried out for general cleaning of the database, in relation to the points of teams and registered people .<br><br>Note: Source: Telehealth Manual for Primary Care, MinSaúde, UFRGS 2012, adapted by the MinSaúde technical team.	MSNT5, 2014 annex I, frame 1,*374.1*
463	monitoring and assessment	study of evaluability	adhesion of municipalities	telehealth practices? What factors make this difficult?	What factors favor the adherence of municipalities to the TBR? What factors hinder this adhesion? a) What factors favor the adherence of health professionals featureto accession? b) What is the nature of teleconsulting demands and what do they reveal about PHC professionals?	HAOC2, 2019 6.2.4,*290.1*
464	monitoring and assessment	study of evaluability	articulation between cores	and advantages of TBR in its actions of	How is the articulation between telehealth centers and permanent education actions produced by the State and municipalities? a) What are the main featurecontributions tele-education? b) What is the nature of teleconsulting demands and what do they reveal about PHC professionals?	HAOC2, 2019 6.2.2,*288.1*
465	monitoring and assessment	study of evaluability	cost-effectiveness	feature	Is TBR cost-effective when compared to other ways of supporting the work of AB teams?	HAOC2, 2019 6.2.3,*289.1*
466	monitoring and assessment	study of evaluability	TBRna challenges expansion and qualification of AB		To what extent does TBR respond to the main challenges that characterize the expansion and qualification of AB in the SUS? a) Does TBR respond to the needs of AB teams? bDoes TBR favor the resolution of AB teams? c) Does TBR help to reduce the demand for specialties? d) How do the nuclei relate to state regulatory centers? What can help featureto strengthen this relationship? e) As the TBR cores<br><br>relate to the State Councils of Municipal Health Secretaries COSEMS)? f) Does the TBR contribute to municipalities organizing the dynamics between PC and other SUS care levels, local and state? g) How articulated, strategic and powerful are the relationships between state telehealth centers and state health departments?	HAOC2, 2019 6.2.1,*287.1*
467	monitoring and assessment	study of evaluability	stage of development of core	featurea) in the implementation phase; b) undergoing maintenance;		HAOC2, 2019 4 a),*292.1*
468	monitoring and assessment	study of evaluability	quality	teleconsulting, telediagnosis and teleeducation	What is the quality of the activities of feature offered by the cores?	HAOC2, 2019 6.2.5,*291.1*
469	monitoring and assessment	exogenous	medical effectiveness	indicator		PAHO, 2016 , *200.1*
470	quality monitoring and evaluation	management and of risk	management	feature	The healthcare organization implements and maintains a quality management system for telehealth services that includes: a) a description of organizational requirements, identified risks, prioritized risks and required quality, plans to address each risk (see Clause 6. b) a description of the objective evidence needed to verify that a quality plan has been implemented	ISO13131, 2021 5.1.2,*102.1*
471	monitoring and assessment	risk management	documentation	feature	Does the healthcare organization document a process for safety management in a quality and safety management system that ensures: a) risks to the safety of the care recipient have been considered for all telehealth service processes and are documented in a quality manual; b) mechanisms are in place and documented in quality plans to identify recipients of care in	ISO13131, 2021 6.4.2,*100.1*

#	theme	category	aspect	nature	element(s)	ref
					an increased risk of harm and taking early steps to reduce those risks; c) systems exist and are documented in quality plans to increase the level of care when there is an unexpected deterioration in health status or an expected increase in the risk of such deterioration	
472	monitoring and assessment	risk management	planning	feature	Quality plans are systematically derived by the healthcare organization in accordance with the processes described in Clause 5 and recorded in a quality management system	ISO13131, 2021 6.5.2,*101.1*
473	monitoring and assessment	governance and management	models of monitoring and assessment	feature	Strengthen the use of monitoring models, audit and evaluation for advances and needs of ICT infrastructure and digital health solutions.	PNIIS, 2021 cap II, section I, art 4, XIV,*785.1*
474	monitoring and assessment	impact	generated transfer by telemedicine	indicator	Refers to the number of hospital transfers, after a teleconsultation, due to medical complications. Interpretation: Determines the number of patients admitted to the hospital as a result of a teleconsultation; monitors the number of transfers generated with teleconsultations and confirms the reduction of these. Formula: Number of patient transfers assisted by teleconsultation x 100/Number of patients assisted by teleconsultation. Frequency: Monthly. Indicator Type. Impact. Notes: Indicator related to avoided transfers; the teleconsultation itself can be considered an avoided transfer; to measure the effectiveness of the program, this indicator must show a downward trend.	PAHO, 2016 ,*316.1*
475	monitoring and assessment	indicators	collection of indicators	feature	Baseline indicators are collected. Answer: yes / no / not applicable / don't know	HAOC1, 2019 6,*249.1*
476	monitoring and assessment	indicators	economy of scale	feature	The organization of the telehealth service is based on in achieving an economy of scale in the offer. Answer: yes / no / not applicable / don't know	HAOC1, 2019 6,*255.1*
477	monitoring and assessment	indicators	clinical impact	feature	Solution impacts morbidity outcomes or mortality. Answer: yes / no / not applicable / don't know	HAOC1, 2019 6,*250.1*
478	monitoring and assessment	indicators	clinical impact alternative	feature	Solution impacts surrogate outcomes clinically relevant to patients. Answer: yes / no / not applicable / don't know	HAOC1, 2019 6,*251.1*
479	monitoring and assessment	indicators	impact on macromanagement	indicator	a) queue and waiting time for consultations and specialized exams; It is recommended that these activities are not just a triage to define the patient's complexity. These services should guarantee better equity (serving the most serious patients first), but also incorporate resoluteness, providing that patients can be attended to in places closer to their residence and in less time by telehealth. Note: Not included in the questionnaire.	HAOC1, 2019 6,*263.1*
480	monitoring and assessment	indicators	impact on processes	feature	The solution has an impact on improving the processes of health services/systems included in it. Answer: yes / no / not applicable / don't know	HAOC1, 2019 6,*252.1*
481	monitoring and assessment	indicators	economic viability	feature	Cost studies (effectiveness, utility, minimization) are carried out to ensure the economic viability of the telehealth solution. Answer: yes / no / not applicable / don't know	HAOC1, 2019 6,*254.1*
482	monitoring and assessment	monitoring and audit	audit	feature	Systematic audit mechanisms are in place (by sampling, for example). Answer: yes / no / not applicable / don't know	HAOC1, 2019 5,*244.1*
483	monitoring and assessment	monitoring and audit	assessment of dissatisfaction	feature	Negative reviews given by users receive audit by some senior project professional. Answer: yes / no / not applicable / don't know	HAOC1, 2019 5,*246.1*
484	monitoring and evaluation	monitoring and audit	definition of indicators	feature	Monitoring indicators are defined. Answer: yes / no / not applicable / don't know	HAOC1, 2019 5,*242.1*
485	monitoring and evaluation	monitoring and audit	feedback from results	feature	There is feedback on the results and quality of care for health professionals who implement the telehealth solution. Answer: yes / no / not applicable / don't know	HAOC1, 2019 5,*247.1*
486	monitoring and assessment	monitoring and audit	digital marketing	feature	Monitoring results are published in web environment and widely available. Answer: yes / no / not applicable / don't know	HAOC1, 2019 5,*243.1*
487	monitoring and evaluation	standards and interoperability	impact of standards on health assessment	feature	Using standards helps monitor and evaluate the health system and helps with strategic planning.	BDHI, 2021 15b,*458.1*

#	theme	category	aspect	nature	element(s)	ref
488	monitoring and assessment	plan of monitoring and M&A valuation of digital health	activities for establish the instances of M&A governance for the collaboration	Processes feature	a) instances to be formalized can be defined as: Sponsorship of the Collaboration M&A Plan, Supervision and Strategic Management of the M&A of the Collaboration, Management of the Execution of M&A of the Collaboration Space; b) identification of actors relevant to each instance; c) elaboration of attribution and competence documents; d) formalization and publication of decisions through appropriate instruments.	ESD28, 2020 M&A, 2.1.1,*748.1*
489	monitoring and evaluation	plan of monitoring and M&A valuation of digital health	activities for establish the M&T processes every priority	feature	a) proposal of result indicators and goals aligned with the M&E; b) proposition of goals, phases and deadlines for collaboration, with goals for each priority; c) capture, compilation, analysis, interpretation and qualification of data for calculating the indicators and their reporting to the M&E management instance; d) preparation of a report with individual and consolidated analysis, with the analysis of indicators, according to defined processes, schedules, models and tools; e) monitoring the systematic collection of data and reporting difficulties encountered or opportunities for improvement to higher authorities.	ESD28, 2020 M&A, 2.1.1,*749.1*
490	monitoring and assessment	plan of monitoring and M&A valuation of digital health	activities for formalize and consolidate M&A processes doConnect SUS	preparation of a	a) proposal of result indicators and goals aligned with the M&E Model; b) proposition of goals, phases and deadlines; c) capture, compilation, analysis, interpretation and qualification of data for calculation of indicators and their reporting to the management instance of featureBAD; d) report with individual analysis and consolidated, with the analysis of indicators, according to defined processes, schedules, models and tools; e) monitoring the systematic collection of data and reporting difficulties encountered or opportunities for improvement to higher authorities.	ESD28, 2020 M&A, 1.2.1,*747.1*
491	monitoring and evaluation preparation	organizational	assessment	feature	The institution establishes continuous assessment mechanisms.	ARGMNMM, 2020 I.42,*584.1*
492	monitoring and assessment	Production	quantity of calls	indicator	number of calls made in a period	ICT-health, 2021 Use of technologies of information and from the communication in the area of health: a telehealth in 2021,*842.1*
493	monitoring and assessment	production	teleconsultations carried out	indicator	percentage of teleconsultations in relation to the total number of consultations	ICT-health, 2021 Use of technologies of information and from the communication in the area of health: a telehealth in 2021,*847.1*
494	monitoring and assessment	production	telehealth by specialty	indicator	telehealth services by specialty	ICT-health, 2021 Use of technologies of information and from the communication in the area of health: a telehealth in 2021,*848.1*
495	monitoring and assessment	security and quality of technology	health service surveillance	feature	The relationship between the care recipient, the healthcare organization, and any healthcare supporting organization should be defined in an SLA service level agreement that provides a post-deployment surveillance system to detect deficiencies that occur after the deployment of the healthcare service. telehealth, including equipment or devices used by the care recipient	ISO13131, 2021 13.1.2,*21.1*

#	theme	category	aspect	nature	element(s)	ref
496	monitoring and assessment	telehealth service	no calls emergencies	feature	Telehealth makes it possible to use technology in non-emergency care that does not require direct interaction between patient and health professional.	ICT-health, 2021 Online services line offered to the patient and telehealth, *824.1*
497	monitoring and assessment	telehealth service	social class of patient	indicator	Social class of patients who use telehealth services	ICT-health, 2021 Online services line offered to the patient and telehealth, *837.1*
498	monitoring and assessment	telehealth service	patient's age range	indicator	Age group using the service	ICT-health, 2021 Online services line offered to the patient and telehealth, *835.1*
499	monitoring and assessment	telehealth service	patient's level of education	indicator	Level of education of patients who used the telehealth service	ICT-health, 2021 Online services line offered to the patient and telehealth, *836.1*
500	monitoring and assessment	telehealth service	improvement of service	feature	The use of telehealth improves care.	ICT-health, 2021 Online services line offered to the patient and telehealth, *826.1*
501	monitoring and assessment	telehealth service	worries in service	feature	Concerns that remain under debate: a) technical quality; b) clinical quality; c) security; d) privacy; e) responsibility;	ICT-health, 2021 Online services line offered to the patient and telehealth, *828.1*
502	monitoring and assessment	telehealth service	reduced risk of contamination	feature	The use of telehealth reduces the risk of contamination.	ICT-health, 2021 Online services line offered to the patient and telehealth, *827.1*
503	monitoring and assessment	telehealth service	reduction in resource use	feature	The use of telehealth reduces the use of resources in health facilities.	ICT-health, 2021 Online services line offered to the patient and telehealth, *825.1*
504	monitoring and assessment	telehealth service	service	indicator	Number of establishments by type of service	ICT-health, 2021 Online services line offered to the patient and telehealth, *839.1*
505	monitoring and assessment	systems and services, patterns and interoperability	data analysis	feature	Analytics: The institution uses tools that allow the collection, analysis and presentation of data to improve processes and decision-making guided by information (assistance, operation and management).	IMDS, 2021 , *674.1*
506	monitoring and assessment	systems and services, patterns and interoperability	impact assessment	feature	Impact Assessment: Does the institution assess the impact of the digital health interventions in its indicators financial, operational and assistance.	IMDS, 2021 , *675.1*
507	monitoring and assessment	technology service support	management of incidents	feature	The healthcare organization ensures that procedures are in place to maintain continuity and reliability of telehealth service using incident management to manage changes to services	ISO13131, 2021 13.1.3,*23.1*

#	theme	category	aspect	nature	element(s)	ref
508	monitoring and assessment	technical support from technology	Market intelligence	feature	The healthcare organization provides technical support for telehealth services that include collection of Market intelligence	ISO13131, 2021 13.1.8,*50.1*
509	monitoring and assessment	technology	accuracy and reliability	feature	The solution has defined accuracy and reliability over time.<br>Answer: yes / no / not applicable / don't know	HAOC1, 2019 two,*222.1*
510	monitoring and assessment	technology	effectiveness	feature	The solution has effectiveness previously established by adequate clinical studies.<br>Answer: yes / no / not applicable / don't know	HAOC1, 2019 two,*220.1*
511	processes and activities	agents of data processing	in charge of treatment	feature	Art. 41. The controller must indicate the person responsible for the processing of personal data. § 1 The identity and contact information of the person in charge must be publicly disclosed, in a clear and objective manner, preferably on the controller's website. § 2 The activities of the person in charge consist of: I accepting complaints and communications from holders, providing clarifications and adopting measures; II receive communications from the national authority and adopt measures; III guide the entity's employees and contractors regarding the practices to be adopted in relation to the protection of personal data; and IV carry out other attributions determined by the controlling shareholder or established in complementary norms. § 3 The national authority may establish complementary norms on the definition and attributions of the person in charge,	LGPD, 2018 cap VI, art 41, *146.1*
512	processes and activities	agents of data processing	operations log	feature	Art. 37. The controller and the operator must keep a record of the personal data processing operations they carry out, especially when based on legitimate interest.	LGPD, 2018 cap VI, art 37, *142.1*
513	processes and activities	legal aspects and ethical	consent of patient	feature	The solution obtains and records the patient's consent to the purpose of using personal health information.<br><br>Answer: yes / no / not applicable / don't know<br>Note: Exclusive for data use, does not mention the use of the telehealth service.	HAOC1, 2019 3,*229.1*
514	processes and activities	legal aspects and ethical	telemedicine consultation record	feature	The complete record of the consultation, with audio, images and video is not mandatory in face-to-face consultations, the The same principle should be adopted in telemedicine.	CFM2314, 2022 considerations, *1835.1*
515	processes and activities	regulatory aspects	consent of patient	feature	Is there a procedure in place to obtain and document the patient's consent before attend a telehealth visit.	ARGMNMM, 2020 V.106,*646.1*
516	processes and activities	activities	activities of state coordination	feature	a) articulates TBR actions at the state level, in all regions of the state; b) promotes articulation between the three instances of SUS management and the other TBR members; c) creates necessary infrastructure and management conditions, aiming to guarantee the functioning of the TBR; d) promotes the articulation of the TBR with the regulation of the offer of services and the Center for Medical Regulation of Urgências, in a shared and articulated way with the network's points of care;	HAOC3, 2019 frame 6,*299.1*
517	processes and activities	activities	activities of national coordination	feature	a) monitors the implementation and functioning of the TBR; b) evaluates and ensures the achievement of TBR objectives and targets; c) maintains the Virtual Library Telessaúde Brasil BVS APS as a reference and identity of the TBR; d) provides operational guidelines and national benchmarks for evaluating the TBR; e) defines the technological standards of interoperability, content and security, allowing the exchange of information between the systems that enable the TBR and those from SUS, including SCNES; f) defines the set of data that will be part of the electronic health record, based on teleconsultations, aiming at the implementation of a national and longitudinal record; g) constitutes a Monitoring and Evaluation Committee with representation from the Secretariats of the MS, CONASS, CONASEMS, and other ministries and entities that develop actions related to the TBR;	HAOC3, 2019 frame 5,*298.1*
518	processes and activities	activities	committee activities state manager	feature	a) the state management committee is active and promotes the articulation between the state, municipal and teaching institution management instances; c) Join the network	HAOC3, 2019 frame 7,*301.1*

#	theme	category	aspect	nature	element(s)	ref
					collaboration between Technical-Scientific Telehealth State Points and Centers, at regional and national levels; c) develops and implements projects contemplating loco-regional needs;	
519	processes and activities	activities	core activities	feature	a) offers teleconsulting, both synchronous and asynchronous; b) offers telediagnosis for several regions of the state; c) produces SOF regularly (at least 2 per month in the last 12 months); d) develops tele-education actions, based on identified loco-regional needs and in line with the priorities of the national health policy; e) composes and maintains a team of teleconsultants and a clinical staff of reference specialists, compatible with the demand of AB teams; f) maintains the complete composition of the team proposed by the MS (general coordinator, administrative assistant, IT technician, field monitor, regulator, teleconsultants); g) regularly promotes and supports the training of teleconsultants within the scope of the TBR (monthly meetings with teleconsultants and offering training activities); h) monitors and evaluates the TBR within its scope of action, including the analysis of the number of requests for teleconsultations, the response time for service users, the number and pertinence of referrals and requests for exams<br><br>complementary; i) regularly updates information and enters data into the national TBR SMART information system; j) presents an annual activity report that proves the achievement of the goals set out in the Work Plan; k) ensures compliance with the interoperability standards proposed by the TBR; l) supports the development of protocols that include prior request for teleconsultations on<br>procedures, to assess the need for referral or request to the Emergency Medical Regulation Center; m) visits the telehealth points periodically to ensure the quality of the service offered; n) has a communication channel with the telehealth points to resolve doubts;	HAOC3, 2019 frame 8,*302.1*
520	processes and activities	activities	assignments in the construction process and use of SOF		a) teleconsultant: select, considering their monthly production, the teleconsultations with the potential to be transformed into SOF and indicate them to the teleregulator. b) teleregulator: select, among the teleconsultations indicated by the teleconsultants, which ones really have the potential to become SOF and revise it according to the format and data elements established in the standard Term of Reference found in Annex II. c) core: send<br>periodically to BIREME the SOF. It is also responsible for publishing the SOF in ARES/UNASUS after they have been published in BVSAPS. d) BIREME Receive the material sent by the nuclei on a monthly basis and coordinate the review and validation process based on pertinence and relevance criteria for PHC and on updated scientific evidence, with the participation of external consultants as required feature specified in item 5. At the end, publish<br><br>of the SOF in the BVSAPS and facilitate the metadata for indexing in ARES/UNASUS carried out by the nucleus. It is BIREME's responsibility to annually update the SOF on the BVSAPS portal, with the support of consultant reviewers. Questions not approved or indicated for review by external consultants will be returned to the responsible nucleus. e) final user of the SOF is the healthcare professional who will use the SOF for their individual qualification or institutions that produce educational activities in the healthcare area that will use them to produce courses, workshops, or any other OA learning object. This user will rate it according to its usefulness and satisfaction. f) Ministry of Health: monitor and manage the SOF production and availability process at the sites of each nucleus, at BVSAPS and at ARES/UNASUS.	MSNT63, 2014 8,*331.1*
521	processes and activities	activities	collaboration patterns enter core with health depts and schools	feature	a) nucleus that offers actions of continuing education and permanent in articulation with the state education body, establishing sporadic partnerships; b) nucleus that assumes the entire offer of activities that would be the responsibility of the permanent education management,	HAOC3, 2019 section 4.2,*300.1*

#	theme	category	aspect	nature	element(s)	ref
					situation that occurred both due to the weakening and lack of investment offered by the state to permanent education, as well as the strengthening of the core together with the state management; c) disjointed core of state management, which offers actions without knowing the offers of the health department, causing the overlapping of activities and the unintelligent use of resources;	
522	processes and activities	assessment	production of telecardiology exams	indicator	a) number of ECG exams performed in the period (classified, not classified according to technical quality); b) distribution of ECG exams according to the population size of the municipality where the exam was requested; c) distribution of ECG exams according to the classification of the result and the patient's sex, in the analyzed period; d) distribution of ECG exams according to the classification of the result and the patient's age group, in the analyzed period;	HAOC4, 2019 , *315.1*
523	processes and activities	assessment	request teleconsultations	indicator	a) number of requests for teleconsultations; b) response time for service users; c) number and pertinence of referrals and requests for complementary exams;	HAOC3, 2019 frame 8,*303.1*
524	processes and activities	short review term	quantity of teleconsultations	indicator	number of teleconsultations in a period of time	PAHO, 2016 , *161.1*
525	processes and activities	short review term	teleconsultations carried out	indicator	Measuring progress in the telehealth system	PAHO, 2016 , *156.1*
526	processes and activities	short review term	waiting time	indicator	Waiting time between scheduling and carrying out the teleconsultation. Measures the advantage of the telemedicine service in relation to the common consultation, and the time saved in patient care	PAHO, 2016 , *158.1*
527	processes and activities	short review term	query waiting time	indicator	Waiting time between scheduled time and consultation time	PAHO, 2016 , *160.1*
528	processes and activities	long evaluation term	monthly appointments	indicator	number or percentage of patients monitored by telemedicine	PAHO, 2016 , *169.1*
529	processes and activities	long evaluation term	monitored patients	indicator	number or percentage of patients monitored by telemedicine	PAHO, 2016 , *168.1*
530	processes and activities	average rating term	adoption of telemedicine	indicator	number of hospitals that have adopted telemedicine in relation to the total number of hospitals	PAHO, 2016 , *163.1*
531	processes and activities	average rating term	impact of teleconsultations	indicator	relationship between teleconsultations performed and relative decrease in regular consultations	PAHO, 2016 , *162.1*
532	processes and activities	average rating term	number of teleconsultations differentiated	indicator	number of differentiated teleconsultations	PAHO, 2016 , *164.1*
533	processes and activities	average rating term	idle time	indicator	number of hours the service was not used	PAHO, 2016 , *165.1*
534	processes and activities	knowledge skilled	protocols of telehealth	feature	There are telehealth care protocols.	ARGMNMM, 2020 V.109,*649.1*
535	processes and activities	consent informed	consent informed		The healthcare organization and healthcare professional obtain informed consent from the care recipient, which confirms: a) permission to carry out health care activities, voluntarily given by a care recipient having competence or a representative, after having been informed about feature the purpose and possible outcomes of the activities; B)<br><br>that the care recipient understood the relative advantages and disadvantages of receiving care through telehealth services; c) that the care recipient understood the information provided; d) that the recipient of care was given the option to read the information provided at a later date.	ISO13131, 2021 11.1.3,*63.1*
536	processes and activities	consent informed	free consent and enlightened	feature	Information about the identified patient can only be transmitted to another professional with prior permission from the patient, with their free and informed consent and with security protocols capable of guaranteeing the confidentiality and integrity of the information.	CFM2314, 2022 considerations, *1838.1*
537	processes and activities	queries, orders and prescriptions	documentation of Query	feature	The organization and healthcare professional determine: a) how and by which professional(s) a consultation using telehealth services is documented; b) the means by which the consultation is documented; c) the elements of the consultation that must be documented or linked to a professional health record	ISO13131, 2021 14.1.5,*10.1*

#	theme	category	aspect	nature	element(s)	ref
538	processes and activities	queries, orders and prescriptions	responsibility for assistance delivery	feature	The organization and healthcare professional determine who is responsible for delivering each type of health care, including responsibilities for health ordering exams, preparing prescriptions and monitoring.	ISO13131, 2021 14.1.5, *11.1*
539	processes and activities	continuity of Careful	care plan	feature	The healthcare organization has alternative and subsequent care plans to enable continuity of healthcare if information and communication technologies fail	ISO13131, 2021 10.1.4, *79.1*
540	processes and activities	coordination and scheduling	scheduling of places and equipment	feature	The health organization has a system for coordinating and scheduling equipment and facilities needed for consultations through telehealth services	ISO13131, 2021 14.1.6, *14.1*
541	processes and activities	coordination and scheduling	scheduling of people	feature	Does the healthcare organization have a system of coordination and scheduling of necessary people for consultations through telehealth services	ISO13131, 2021 14.1.6, *13.1*
542	processes and activities	schedule of execution	physical percentage of execution of a activity in the period object total	indicator	Subjective indication or calculation of the percentage of execution of the activity based on the total execution over the expected total execution in the total period of the object. Note: There is "indication" because in some reports the activity/goal is not quantifiable; in this case, the coordinator informs a percentage indicated according to his own subjective evaluation.	DESD, 2021 , *1773.1*
543	processes and activities	schedule of execution	physical percentage of execution of a activity in the last period	indicator	Subjective indication or calculation of the percentage of execution of the activity based on the total execution over the total foreseen execution in the last period (for example, 4 months) Note: There is "indication" because in some reports the activity/goal is not quantifiable; in this case, the coordinator informs a percentage indicated according to his own subjective evaluation.	DESD, 2021 , *1776.1*
544	processes and activities	schedule of execution	total execution of an activity for the total period of the object	indicator	Description or total number of execution of the activity for the total period of the object. (examples: 20 hours, 2 events, 150 participants, 2 teleeducation activities, 160 teleconsultations/month indicating the total in the period, 40 telehealth points to be included in the period, 2 SOF/ month indicating the total in the period	DESD, 2021 , *1772.1*
545	processes and activities	schedule of execution	running total of an activity for the last period	indicator	Description or total number of execution of the activity for the last period (for example, 4 months) (examples: 20 hours, 2 events, 150 participants, 2 tele-education activities, 160 teleconsultations/month indicating the total in the period, 40 points of telehealth to be included in the period, 2 SOF/month indicating the total in the period	DESD, 2021 , *1775.1*
546	processes and activities	schedule of execution	expected total of execution of a activity for the total period of the object	indicator	Description or expected total number of execution of the activity for the total period of the object. (examples: 120 hours, 4 events, 650 participants, 6 tele-education activities, 160 teleconsultations/month indicating the total in the period, 80 telehealth points to be included in the period, 2 SOF/month indicating the total in the period	DESD, 2021 , *1771.1*
547	processes and activities	schedule of execution	expected total of execution of a activity for the last period	indicator	Description or expected total number of execution of the activity for the last period (for example, 4 months) (examples: 120 hours, 4 events, 650 participants, 6 tele-education activities, 160 teleconsultations/month indicating the total in the period, 80 telehealth points to be included in the period, 2 SOF/month indicating the total in the period	DESD, 2021 , *1774.1*
548	processes and activities	schedule of execution	execution value of an activity in the last period	indicator	value in reais of execution of the activity based on the total execution over the total foreseen execution in the last period (for example, 4 months) Note: There is an "indication" because in some reports the activity/goal is not quantifiable; in this case, the coordinator informs a percentage indicated according to his own subjective evaluation.	DESD, 2021 , *1777.1*
549	processes and activities	definition	definitionSOF	feature	Systematized response, built on the basis of bibliographic review, the best scientific and clinical evidence and the ordering role of primary health care, questions arising from teleconsultations, and selected from criteria of relevance and pertinence in relation to SUS guidelines. Teleconsultations with the potential to become SOF can be defined as those created from subjects relevant to the SUS, based on its principles and guidelines, with	MSNT63, 2014 2, 3, *328.1*

#	theme	category	aspect	nature	element(s)	ref
					possibility of covering a region or specific health policies. It should clearly state the essential knowledge to solve the problem and contribute to the continuing education of health workers, with a view to expanding the resolution capacity of similar cases. The SOF's main objective is to help solve problems in the health system through its formative role, contributing to increase the system's problem-solving ability through the qualification and updating of professionals, acting as an Object of Learning OA for the Permanent Education in Health EPS of workers. Note: Definition from Ordinance 2546 of 10/27/2011	
550	processes and activities	definition	report, certificate or prescription in telemedicine	Patient identification and data (address and place	Art. 13. In the case of remote issuance of a report, certificate or medical prescription, the medical record must obligatorily include: a) Identification of the doctor, including name, CRM, professional address; B) feature informed of the service); c) Date and time stamp; d) Signature with digital certification of the physician in the ICPBrasil standard or another legally accepted standard; e) which was issued in the form of telemedicine.	CFM2314, 2022 art 13,*1852.1*
551	processes and activities	definition	telesurgery	and mediated by safe interactive technologies.	Art. 9 Telesurgery is the performance of a surgical procedure at a distance, using equipment featurerobotic Single paragraph. Robotic telesurgery is disciplined in specific CFM resolution.	CFM2314, 2022 art 9,*1848.1*
552	processes and activities	definition	conference call	feature	Art. 14. Medical procedure teleconferencing by synchronous video transmission may be carried out for the purposes of assistance, education, research and training, with authorization from the patient or his/her legal guardian, provided that the group receiving images, data and audios be composed exclusively of physicians and/or medical students, all duly identified and accompanied by their tutors. § 1 In the case of using telepresence technologies, the same premises must be followed. § 2 In multidisciplinary events, CFM Resolution No. 1,718/2004 or later must also be complied with in its entirety. § 3 In the teleconference, the training objectives must not compromise the quality of care nor generate an unnecessary increase in time, which could compromise the patient's recovery,	CFM2314, 2022 aet 14,*1853.1*
553	processes and activities	definition	teleconsultation		Art. 6 Teleconsultation is a non-face-to-face medical consultation, mediated by DICTs, with doctor and patient located in different spaces. § 1 The face-to-face consultation is the gold standard of reference for medical consultations, with telemedicine being a complementary act. § 2 In cases of chronic diseases or diseases that require long-term follow-up, a face-to-face consultation must be carried out with the patient's attending physician, at intervals not exceeding 180 days. § 3 The establishment of a doctor-patient relationship can be carried out virtually, in the first consultation, provided that it meets the featurephysical and technical conditions set forth in this resolution, complying with good medical practices, and follow-up with a face-to-face medical consultation. § 4 The doctor must inform the patient of the limitations inherent to the use of teleconsultation, due to the impossibility of carrying out a complete physical examination, and the doctor may request the patient's presence to complete it. § 5 It is the right of both the patient and the doctor to choose to interrupt the remote care, as well as to opt for the face-to-face consultation, with respect to the Term of Free and Informed Consent pre-established between the doctor and the patient.	CFM2314, 2022 art 6,*1845.1*
554	processes and activities	definition	teleconsulting	others featureprofessionals, with the aim of providing	Art. 12. Medical teleconsulting is a consulting act mediated by DICTs between physicians, managers and clarifications on administrative procedures and health actions.	CFM2314, 2022 art 12,*1851.1*

#	theme	category	aspect	nature	element(s)	ref
555	processes and activities	definition	telediagnosis	feature	Art. 8 Telediagnosis is a medical act at a distance, geographically and/or temporally, with the transmission of graphs, images and data for the issuance of a report or opinion by a physician with a qualification record of RQE specialist in the area related to the procedure, in accordance with the request of the attending physician. Single paragraph. The services where the exams are being carried out must have a responsible medical technician.	CFM2314, 2022 art 8, *1847.1*
556	processes and activities	definition	teleinterconsultation	feature	Art. 7 Teleinterconsultation is the exchange of information and opinions between physicians, with the aid of DICTs, with or without the presence of the patient, for diagnostic or therapeutic, clinical or surgical assistance. Single paragraph. O assistant physician responsible for teleinterconsultation must be, obligatorily, the physician responsible for the face-to-face monitoring. The other doctors involved can only be held responsible for their actions.	CFM2314, 2022 art 7, *1846.1*
557	processes and activities	definition	telemedicine	feature	Art. 1 Define telemedicine as the practice of medicine mediated by Digital, Information and Communication Technologies (DICTs), for the purposes of assistance, education, research, disease prevention and injuries, health management and promotion. Article 2 A telemedicine, in real time online (synchronous) or offline (asynchronous), by multimedia technology, is allowed within the national territory, under the terms of this resolution.	CFM2314, 2022 art 1, *1832.1*
558	processes and activities	definition	telemonitoring or medical telesurveillance	feature	Art. 10. Telemonitoring or medical telesurveillance is the act performed under coordination, indication, guidance and supervision by a doctor for remote monitoring or surveillance of health and/or disease parameters, through clinical evaluation and/or direct acquisition of images , signals and data from equipment and/or devices aggregated or implantable in patients at home, in a medical clinic specialized in chemical dependency, in a long-term care facility for the elderly, in a clinical or home hospitalization regime or when transferring a patient until their arrival at the health establishment. § 1 Telemonitoring includes the collection of clinical data, its transmission, processing and handling, without the patient having to travel to a health unit. § 2º It must be carried out by indication and justification of the patient's attending physician, with guarantee of security and confidentiality, both in transmitting and receiving data. § 3 The transmission of data must be carried out under the technical responsibility of the patient's institution. § 4 The interpretation of data and issuance of reports must be carried out by a physician regularly enrolled in the CRM of his/her jurisdiction and with a record of qualification as an RQE specialist in the area related to specialized examinations. § 5 The coordination of the medical service must promote the proper training of local human resources, including patients, who will be able to intermediate the care. § 6 All data resulting from telemonitoring, including test results, clinical evaluation and prescription and professionals involved must be properly recorded in the patient's medical record.	CFM2314, 2022 art 10, *1849.1*
559	processes and activities	definition	telehealth	feature	The term telehealth applies to the use of information and communication technologies to transfer information of data and clinical, administrative and health education, by health professionals, respecting their legal competences.	CFM2314, 2022 considerations, *1834.1*
560	processes and activities	definition	telehealth and telemedicine	feature	The term telehealth is broad and encompasses other healthcare professionals, while telemedicine is specific to medicine and refers to acts and procedures performed or under the responsibility of physicians.	CFM2314, 2022 considerations, *1833.1*
561	processes and activities	definition	telescreening	feature	Art. 11. Medical telescreening is the act performed by a doctor, with assessment of the patient's symptoms, at a distance, for outpatient or hospital regulation, with defining and directing the patient to the appropriate type of assistance he needs or to a specialist. § 1 The doctor must highlight and record that it is only a diagnostic impression and gravity, the doctor has autonomy of the decision of which	CFM2314, 2022 art 11, *1850.1*

#	theme	category	aspect	nature	element(s)	ref
					resource will be used for the benefit of the patient, not to be confused with medical consultation. § 2 In medical telescreening, the health establishment/system must offer and guarantee the entire regulatory system for referring patients under its responsibility.	
562	processes and activities	definition	reference term for submission to BIREME of teleconsultations with potential for SOF	bibliography; field 5	field 1 question; field 2 botton line - the evidence-based answer; field 3 category of featureevident; field 4 selected - requesting professional; field 6 descriptors; field 7 responsibility/author;	MSNT63, 2014 annex II,332.1
563	processes and activities	delegation of healthcare	commitment of healthcare		The health organization ensures that the health delegations of all care recipients using telehealth services are based on a health care commitment consisting of featurea promise from the care recipient that he/she will perform health care activities. It also means that the healthcare professional accepts and confirms the pending healthcare delegations contained in the proposed plan of care.	ISO13131, 2021 11.1.2,61.1
564	processes and activities	delegation of healthcare	consent		The healthcare organization ensures that the health delegations of all care recipients using telehealth services are based on informed consent from the care recipient giving permission to carry out care activities. featureto health, given voluntarily having competence or by its representative, after having been informed of the purpose and possible results of the activities or a legal authorization by express provision in the legislation which, in certain circumstances, may nullify the need for informed consent	ISO13131, 2021 11.1.2,60.1
565	processes and activities	delegation of healthcare	documentation of Query	a professional	The healthcare organization ensures that the health delegations of all care recipients using telehealth services are based on an assessment featureof needs during which health considers these health needs of the care recipient and determines the health activities to be recorded in the care plan.	ISO13131, 2021 11.1.2,62.1
566	processes and activities	performance	inquiries from specialty by telemedicine	indicator	Measures the number of consultations by specialty. Interpretation: Helps determine demand for queries. Formula: Sum of consultations by specialty in telemedicine x 100/Total of consultations in telemedicine. Frequency: Monthly. Indicator Type. Performance.	PAHO, 2016 , 210.1
567	processes and activities	performance	hours available with specialist	indicator	Establishes the offer of telemedicine consultations available in the program. Interpretation: Total number of possible hours physicians have for patient care via telemedicine. Formula: Sum of the total hours available for teleconsultation by specialists x 100/total hours available by specialists. Frequency: Monthly. Indicator Type. Performance. Notes: This indicator can be incorporated into other measures: Increase in hours available to specialized physicians for telemedicine consultations. Comparison with total hours for face-to-face consultation. Comparison with real consultation hours.	PAHO, 2016 , 204.1
568	processes and activities	availability of service	documentation	to the service; b) the times and days on which the	The healthcare organization has included a telehealth service design document considering: a) what times of the day the care recipient can expect featurehave access service will be intentionally unavailable; c) the maximum expected duration of unforeseen service interruptions.	ISO13131, 2021 8.1.3,92.1
569	processes and activities	duration of care	documentation	that are necessary to provide	The specific health organization in a telehealth service design document the durations of featurehealth activities adequate and effective health care for the care recipient	ISO13131, 2021 8.1.4,93.1
570	processes and activities	ecosystem of innovation	care evaluation system	chronic health conditions in clinical practice and that	Encouraging the development of applications that use technologies that allow the routine detection of feature monitor the quality and effectiveness and efficacy of health care.	PNIIS, 2021 chapter II, section VII, art 10, VII, 814.1
571	processes and activities	structure	core actions	featurea) teleconsulting; b) permanent education; c) support	matrix; d) preparation of personnel and teams for	MSPC5, 2017 title IV, chapter I,

#	theme	category	aspect	nature	element(s)	ref
					the use of the service; e) resolution of problems related to the use of the service; f) monitoring and evaluation of the use and quality of the service; and g) monitoring and evaluation of the impact of Telessaúde Brasil Redes in Primary Care and its results in the health care of the population. Note: Origin: p2554, 2011 art 7	section I, subsection V, art 470, *347.1*
572	processes and activities	structure	response time for teleconsultation asynchronous	feature	The regular deadline for sending a response to the asynchronous teleconsultation will be set in protocols established by each technical-scientific telehealth center, in in agreement with the State Management Committee, no within a maximum period of 72 (seventy-two) hours from receipt of the consultation. Note: Origin: p2546, 2011 cap I, art 5	MSPC5, 2017 title IV, cap I, section I, subsection I, art 451, *338.1*
573	processes and activities	structure	Law Suit	feature	Processes: set of operations and functions that must be considered.	ARGMNMM, 2020 1.4.1, *534.1*
574	processes and activities	structure	services provided	feature	a) teleconsulting (synchronous, asynchronous); b) telediagnosis; c) second formative opinion; d) tele-education; Note: Origin: p2546, 2011 cap I, art 2	MSPC5, 2017 title IV, cap I, section I, subsection I, art 448, *335.1*
575	processes and activities	structure	use of evidence scientific	feature	Teleconsultations, telediagnoses and the second formative opinion are answered by the technical-scientific telehealth centers based on the best and most up-to-date clinical and scientific evidence available, adequate and relevant to the context in which the request originated. Single paragraph. The answer should emphasize the knowledge inherent in solving the problem and that will contribute to the ongoing education of the professionals involved, with a view to expanding their capacity and autonomy in solving similar cases. Note: Origin: p2546, 2011 cap I, art 4	MSPC5, 2017 title IV, cap I, section I, subsection I, art 450, *337.1*
576	processes and activities	execution	specialty of teleconsultation	indicator	Teleconsultation specialties available	DESD, 2021 , *1790.1*
577	processes and activities	execution	specialties answered	indicator	Number of specialties served	DESD, 2021 , *1816.1*
578	processes and activities	execution	number of exams performed	indicator	Number of exams performed	DESD, 2021 , *1795.1*
579	processes and activities	execution	number of reports performed	indicator	Number of reports performed	DESD, 2021 , *1818.1*
580	processes and activities	execution	number of SOFs submitted	indicator	SOF submitted/month/nucleus	DESD, 2021 , *1820.1*
581	processes and activities	execution	number of teleconsultations for ICU teams	indicator	Number of teleconsultations for ICU teams	DESD, 2021 , *1817.1*
582	processes and activities	execution	points of active teleconsulting	indicator	Active teleconsulting points per month	DESD, 2021 , *1822.1*
583	processes and activities	execution	total production	indicator	total production	DESD, 2021 , *1800.1*
584	processes and activities	execution	number of sessions of video collaboration	indicator	video collaboration sessions (webconferencing, videoconferencing, streaming and telepresence), demands from professionals and students	DESD, 2021 , *1784.1*
585	processes and activities	execution	quantity of teleconsultations outpatient video	indicator	Video outpatient teleconsultations	DESD, 2021 , *1789.1*
586	processes and activities	execution	quantity of video teleconsultations	indicator	video teleconsultations	DESD, 2021 , *1788.1*
587	processes and activities	execution	quantity of telediagnostics of ECG	indicator	ECG telediagnosis	DESD, 2021 , *1786.1*
588	processes and activities	execution	quantity of teleguidance by chat	indicator	teleguidance via chat	DESD, 2021 , *1787.1*
589	processes and activities	execution	quantity of video televisions	indicator	video televisions to patients	DESD, 2021 , *1785.1*
590	processes and activities	execution	waiting time for exam	indicator	waiting time for exam	DESD, 2021 , *1792.1*
591	processes and activities	execution of plans of care	identification of actors	characteristic	The organization guarantees that: a) prior to the execution of the plan of care the health professional informs the	ISO13131, 2021 11.1.9, *71.1*

#	theme	category	aspect	nature	element(s)	ref
					care receiver about the actors who will participate in the provision of health care and the various functions they perform; b) before carrying out the care plan, the health professional informs the recipient of care who is responsible for each part of the care to be provided	
592	processes and activities	execution of plans of care	information to receivers	feature	The organization ensures that the health professional, at regular intervals during the execution of the care plan, asks the care recipients and possible informal caregivers involved if they understood and followed the advice and instructions given	ISO13131, 2021 11.1.9,*73.1*
593	processes and activities	execution of plans of care	services of follow-up	feature	Appropriate follow-up services for care recipients are coordinated in collaboration with the care recipient and healthcare professionals.	ISO13131, 2021 11.1.9,*74.1*
594	processes and activities	execution of plans of care	care receiver support	feature	The organization ensures that care recipients have been informed about how complaints about a telehealth service can be made and how they will be handled	ISO13131, 2021 11.1.9,*72.1*
595	processes and activities	training and training of human Resources	qualification of work processes	feature	Encouraging the qualification of health work processes, including new digital solutions, considering them management and audit activities of the health system and care management.	PNIIS, 2021 cap iI, section V, art 8, I,*798.1*
596	processes and activities	event management adverse	care plan	feature	Does the healthcare organization clearly record in the plan of care any possible limitations and risks to the health care activities due to the use of telehealth services, and takes steps to reduce such limitations or risks	ISO13131, 2021 10.1.7,*83.1*
597	processes and activities	registry management healthcare professional	care plan	feature	The healthcare organization develops care plans that include descriptions of information arising from a healthcare activity and ensure that this information is stored in the registry. healthcare professional. The information that will be recorded during health care activity should be based on clinical guidelines. The format in which it is recorded and transmitted must be based on adequate technical standards	ISO13131, 2021 10.1.8,*84.1*
598	processes and activities	governance and management of information and technology of health information	models and processes practical	feature	Develop convincing models and processes for practical application of clinical, epidemiological, demographic, environmental and other information in everyday life areas of technical-scientific knowledge that are useful for understanding the complex health situations in their respective territories, according to the competences of the three spheres of government.	ABRASCO, 2020 1st dimension, 1, *498.1*
599	processes and activities	governance and resources organizational	digital marketing	feature	Social Networks and Digital Marketing: The institution has a presence strategy on social networks and digital marketing.	IMDS, 2021 , *691.1*
600	processes and activities	indicators	indicators of process	indicator	a) proportion of active professionals in a given period (over the total number of professionals); B) service availability (uptime/total contracted time); c) frequency of use by professional (in a given period of time); d) population average utilization rate (example: number of telediagnoses per capita per city); e) response time;<br><br>Note: Not included in the questionnaire. Origin: Donabedian, 1988	HAOC1, 2019 6,*257.1*
601	processes and activities	indicators	indicators of clinical result assistance	indicator	a) number of patients on the waiting list for specialized care; b) average waiting time for specialized care; c) rate of readmissions; d) mean length of stay; e) reduction of mortality;<br>Note: Not included in the questionnaire. Origin: Donabedian, 1988	HAOC1, 2019 6,*258.1*
602	processes and activities	indicators	justification for solution	feature	The solution has a volume or frequency of use that justify its incorporation or maintenance.<br>Answer: yes / no / not applicable / don't know	HAOC1, 2019 6,*253.1*
603	processes and activities	indicators	typical measurements of productivity	indicator	a) number of reports; b) number of teleconsultations; c) number of participants; d) number of courses and students; The adequate choice of indicators is undoubtedly one of the main tasks of telehealth services because most of the information from these services is focused on productivity measures, which does not necessarily mean improved access,	HAOC1, 2019 6,*261.1*

#	theme	category	aspect	nature	element(s)	ref
					quality and/or cost reduction. And this information is even more controversial when the provision of the service is not associated with a specific patient (and preferably with a unique identifier, such as the SUS card, such as unidentified teleconsultations and teleeducation activities. Note: Not included in the questionnaire.	
604	processes and activities	methodology and strategies operational	core activities	feature	offer teleconsulting; offer telediagnosis (electrocardiogram, retinography, radiology, dermatology, ophthalmology, etc.); offer classes, courses and short courses; prepare and publish second formative opinion; install telehealth points; contact AB coordinators in the municipalities and state; offer teleconsulting; train the work team; train requesters about the services; monitor the quality of services offered (completeness, non-response, replicas, etc.); plan classes, courses and short courses; contact teleeducators; manage and disseminate agenda; elaboration of didactic or dissemination material (recording, transmission, publishing, posting); formal course and mini-course registration; register platform users; solve general doubts; issue certificates; agree goals for the use of services; preparing usage and production reports; hire work staff; monitor connection, internet and infrastructure; manage financial resources; offer<br><br>telediagnostics on the National Telediagnosis Platform; evaluate the operation, activities and quality of services provided by the nucleus; plan and manage core actions; deploy core services; research and implement innovations for services; prepare physical and logical structure for core services	DESD, 2021 , *1770.1*
605	processes and activities	methodology and strategies operational	percentage of execution of the object	indicator	Indication of the execution percentage in the object's total period.	DESD, 2021 , *1769.1*
606	processes and activities	monitoring and audit	guidelines	feature	For any of the activities in telehealth, standardization must be pursued by the performing team. Guidelines and standardized responses should guide the execution of tasks, especially when these involve decision-making support activities (more subject to interpretation biases or even the bibliography used). In this case, not only should the search for answers encompass the best level of evidence, but also the uniformity of conduct should be an agenda for the team. For this problem, it<br><br>Interesting is the development of automation tools (for issuing reports and searching for answers).<br><br>Note: Not included in the questionnaire.	HAOC1, 2019 5,*245.1*
607	processes and activities	the planning of the Careful	efficacy and safety	protocols: a) that demonstrate	The healthcare organization bases its care plans and healthcare activities on featureclinical guidelines and evidence of their efficacy and safety, b) and/or that they are recognized by an appropriate professional society.	ISO13131, 2021 10.1.3,*78.1*
608	processes and activities	preparation organizational	work load	feature	A workload is defined to launch telehealth program in the current environment.	ARGMNMM, 2020 I.31,*573.1*
609	processes and activities	preparation organizational	impact on the agenda traditional	feature	There is a change in patient care agendas for the need for non-face-to-face consultations.	ARGMNMM, 2020 I.36,*578.1*
610	processes and activities	preparation organizational	offer of teleconsultations	feature	The institution has experience in providing services through virtual consultations.	ARGMNMM, 2020 I.11,*548.1*
611	processes and activities	preparation organizational	offer of asynchronous teleconsultations	feature	The institution has experience in providing services through asynchronous virtual consultations.	ARGMNMM, 2020 I.14,*551.1*
612	processes and activities	preparation organizational	offer of synchronous teleconsultations	feature	The institution has experience in providing services through synchronous virtual consultations.	ARGMNMM, 2020 I.13,*550.1*
613	processes and activities	preparation organizational	Offer of telemonitoring	feature	The institution has experience in remotely monitoring patients.	ARGMNMM, 2020 I.12,*549.1*
614	processes and activities	preparation organizational	first opinion	feature	The institution has experience in providing services of first opinion teleconsultations.	ARGMNMM, 2020

#	theme	category	aspect	nature	element(s)	ref
						I.16,553.1
615	processes and activities	preparation organizational	second opinion	feature	The institution has experience in providing second opinion teleconsultation services.	ARGMNMM, 2020 I.15,552.1
616	processes and activities	preparation organizational	telecommuting	feature	Telehealth services are implemented in the form of telework.	ARGMNMM, 2020 I.34,576.1
617	processes and activities	preparation organizational	I use messages instant	feature	The institution has an initiative to use instant and text messages for health promotion.	ARGMNMM, 2020 I.10,547.1
618	processes and activities	procedures of emergency	protocols of emergency	feature	The healthcare organization has comprehensive emergency protocols and escalated procedures to manage acute clinical situations that arise during the delivery of telehealth services	ISO13131, 2021 10.1.5,80.1
619	processes and activities	process	definition of roles	the feature	The roles, functions and responsibilities associated with telehealth services are defined for all employees involved.	ARGMNMM, 2020 I.43,585.1
620	processes and activities	process	definition of roles	the feature	The roles, functions and responsibilities associated with telehealth services are defined for all administrative employees.	ARGMNMM, 2020 I.44,586.1
621	processes and activities	process	technical failures	feature	There are standardized procedures for communicating and document possible technical failures that may affect the assistance activity during a consultation.	ARGMNMM, 2020 I.50,592.1
622	processes and activities	process	notification of incidents	feature	There are procedures for reporting incidents and adverse situations that occurred during consultations with telehealth.	ARGMNMM, 2020 I.49,591.1
623	processes and activities	process	service processes of telehealth	feature	a) telehealth micro-level processes; a1 patient data is captured; a2 patient data are transmitted to where they will be analyzed; a3 data are analyzed and converted into useful information (diagnosis); a4) this useful information is then transmitted back; a5 from the received information an action is performed; a6 technical data transmission processes; b) medium-level telehealth processes; c) telehealth macro-level processes (generally non-specific);	TMSMM, 2013 3.2,431.1
624	processes and activities	process	request teleconsultations based on regulation protocols	indicator	Description: % of teleconsulting requests based on regulatory protocols approved by the CIB; Numerator: number of requests from teleconsultants based on regulation protocols; Denominator: total number of requests from teleconsultants; Unit: %; Source: request data;	MSNT5, 2014 annex I, frame 3,397.1
625	processes and activities	process	request teleconsultations by professional category	indicator	Description: number of teleconsultation requests answered by professional category; Numerator: total requests per professional category (use CBO; Unit: requests/month; Source: data from requests; Note: 1 usage fee will be calculated from the data informed of the requests answered from active points and points registered as implanted; 2 Questions received and answered during tele-education activities should not be counted as teleconsulting. Note: Source: Telehealth Manual for Primary Care, MinSaúde, UFRGS 2012.	MSNT5, 2014 annex I, frame 2,385.1
626	processes and activities	process	request teleconsultations by health team	indicator	Description: number of teleconsultation requests answered by the health team; Numerator: total requests per team; Unit: requests/month; Source: request data; Note: 1 usage fee will be calculated from the data informed of the requests answered from active points and points registered as implanted; 2 the doubts received table 2,381.1 and answered during tele-education activities should not be counted as teleconsulting. Note: Source: Telehealth Manual for Primary Care, MinSaúde, UFRGS 2012.	MSNT5, 2014 annex I,
627	processes and activities	process	request teleconsultations by state	indicator	Description: number of answered requests for teleconsultations in the state; Numerator: total requests in the state; Unit: requests/month; Source: request data; Note: 1 usage fee will be calculated from the data informed of the requests answered from active points and points registered as implanted; 2 Questions received and answered during tele-education activities should not be counted as teleconsulting.	MSNT5, 2014 annex I, frame 2,379.1

#	theme	category	aspect	nature	element(s)	ref
					Note: Source: Telehealth Manual for Primary Care, MinSaúde, UFRGS 2012.	
628	processes and activities	process	request teleconsultations by member of management	indicator	Description: number of teleconsultation requests answered by management member; Numerator: total requests per management member; Unit: requests/month; Source: request data; Note: 1 usage fee will be calculated from the data informed of the requests answered from active points and points registered as implanted; 2 Questions received and answered during tele-education activities should not be counted as teleconsulting.<br><br>Note: Source: Telehealth Manual for Primary Care, MinSaúde, UFRGS 2012.	MSNT5, 2014 annex I, frame 2,*382.1*
629	processes and activities	process	request teleconsultations by County	indicator	Description: number of teleconsultation requests answered by municipality; Numerator: total requests per municipality; Unit: requests/month; Source: request data; Note: 1 usage fee will be calculated from the data reported from the answered requests for active points and points<br><br>registered as implanted; 2 the doubts received table 2,*380.1* and answered during tele-education activities should not be counted as teleconsulting. Note: Source: Telehealth Manual for Primary Care, MinSaúde, UFRGS 2012.	MSNT5, 2014 annex I,
630	processes and activities	process	request teleconsultations by telehealth point	indicator	Description: number of teleconsultation requests answered per point; Numerator: total requests per telehealth point; Unit: requests/month; Source: request data; Note: 1 usage fee will be calculated from the data reported from the answered requests for active points and points<br><br>registered as implanted; 2 the doubts received table 2,*383.1* and answered during tele-education activities should not be counted as teleconsulting. Note: Source: Telehealth Manual for Primary Care, MinSaúde, UFRGS 2012.	MSNT5, 2014 annex I,
631	processes and activities	process	request teleconsultations by professional	indicator	Description: number of teleconsultation requests answered by professional; Numerator: total requests per professional; Unit: requests/month; Source: request data; Note: 1 usage fee will be calculated from the data reported from the answered requests for active points and points<br><br>registered as implanted; 2 the doubts received table 2,*384.1* and answered during tele-education activities should not be counted as teleconsulting. Note: Source: Telehealth Manual for Primary Care, MinSaúde, UFRGS 2012.	MSNT5, 2014 annex I,
632	processes and activities	process	request teleconsultations by asynchronous type	indicator	Description: number of asynchronous teleconsultation requests answered; Numerator: total number of asynchronous teleconsultation requests; Unit: requests/month; Source: request data; Note: 1 usage fee will be calculated from the data informed of the requests answered from active points and points registered as implanted; 2 Questions received and answered during tele-education activities should not be counted as teleconsulting.<br><br>Note: Source: Telehealth Manual for Primary Care, MinSaúde, UFRGS 2012.	MSNT5, 2014 annex I, frame 2,*387.1*
633	processes and activities	process	request teleconsultations by synchronous type	indicator	Description: number of synchronous teleconsultation requests answered; Numerator: total number of synchronous teleconsultation requests; Unit: requests/month; Source: request data; Note: 1 usage fee will be calculated from the data informed of the requests answered from active points and points registered as implanted; 2 Questions received and answered during tele-education activities should not be counted as teleconsulting.<br><br>Note: Source: Telehealth Manual for Primary Care, MinSaúde, UFRGS 2012.	MSNT5, 2014 annex I, frame 2,*386.1*
634	processes and activities	process	request telediagnosis by team	indicator	Description: number of requests with examination performed and report sent to the requester per team; Numerator: total number of reports issued by the team; Unit: requests/month; Source: request data;	MSNT5, 2014 annex I, frame 3,*393.1*
635	processes and activities	process	request telediagnosis by	indicator	Description: number of requests with examination performed and report sent to the applicant by state; Numerator:	MSNT5, 2014 annex I,

#	theme	category	aspect	nature	element(s)	ref
			state		total number of reports issued by state; Unit: requests/month; Source: request data;	frame 3, *391.1*
636	processes and activities	process	request telediagnosis by County	indicator	Description: number of requests with examination carried out and report sent to the applicant by municipality; Numerator: total number of reports issued by municipality; Unit: requests/month; Source: request data;	MSNT5, 2014 annex I, frame 3, *392.1*
637	processes and activities	process	request telediagnosis by point	indicator	Description: number of requests with examination performed and report sent to the applicant per point; Numerator: total number of reports issued per point; Unit: requests/month; Source: request data;	MSNT5, 2014 annex I, frame 3, *394.1*
638	processes and activities	process	request telediagnosis by type	indicator	Description: number of requests with examination performed and report sent to the applicant by type (teleECG, telespirometry, teleradiology, teleophthalmology, etc.) - use the SAI table; Numerator: total number of reports issued by type of exam; Unit: requests/month; Source: request data;	MSNT5, 2014 annex I, frame 3, *395.1*
639	processes and activities	process	request telediagnostics based on regulation protocols	indicator	Description: % of telediagnosis requests based on regulatory protocols approved by the CIB; Numerator: number of telediagnosis requests based on regulation protocols; Denominator: total number of telediagnosis requests; Unit: %; Source: request data;	MSNT5, 2014 annex I, frame 3, *396.1*
640	processes and activities	process	utilization of teleconsultations	indicator	Description: number of active points (at least one monthly service use) in teleconsultations; Numerator: total points that had teleconsultations answered; Unit: points/month; Source: request data; Note: 1 usage fee will be calculated from the data informed of the requests<br><br>answered from active points and points registered as implanted; 2 Questions received and answered during tele-education activities should not be counted as teleconsulting. Note: Source: Telehealth Manual for Primary Care, MinSaúde, UFRGS 2012. It has an error in the definition: the original contains an error<br><br>"requests/month".	MSNT5, 2014 annex I, frame 2, *388.1*
641	processes and activities	process	utilization of telediagnostics	indicator	Description: number of active points (at least one monthly service use) in telediagnostics; Numerator: total points scored<br>telediagnostics; Unit: points/month; Source: request data; Note: 1 usage fee will be calculated from the data informed of the requests answered from active points and points registered as implanted; 2 Questions received and answered during tele-education activities should not be counted as teleconsulting. Note: Source: Telehealth Manual for Primary Care, MinSaúde, UFRGS 2012. It has an error in the definition: the original contains an error<br><br>"requests/month".	MSNT5, 2014 annex I, frame 2, *389.1*
642	processes and activities	processes of healthcare	medical autonomy in telemedicine	feature	Art. 4 The doctor is assured the autonomy to decide whether to use or refuse telemedicine, indicating face-to-face care whenever he deems it necessary. § 1 Medical autonomy is limited to the benefit and non-maleficence of the patient, in line with ethical and legal precepts. § 2 Medical autonomy is directly related to responsibility for the medical act. § 3 The physician, when assisting via telemedicine, must provide a line of care to the patient, aiming at their safety and the quality of care, indicating face-to-face care when there is evidence of risks.	CFM2314, 2022 art 4, *1843.1*
643	processes and activities	processes of healthcare	collaboration	feature	The healthcare organization defines clinical guidelines and protocols that support collaboration between health organizations and health professionals and that describe which elements of the health professional record are needed to support collaboration	ISO13131, 2021 10.1.2, *77.1*
644	processes and activities	processes of healthcare	provision of healthcare	feature	The healthcare organization defines clinical guidelines and protocols that support collaboration between health organizations and health professionals and that describe the processes that integrate the provision of health care	ISO13131, 2021 10.1.2, *75.1*
645	processes and activities	processes of healthcare	responsibility	feature	The healthcare organization defines guidelines and protocols clinicians who support collaboration between	ISO13131, 2021 10.1.2, *76.1*

#	theme	category	aspect	nature	element(s)	ref
					health organizations and health professionals and that describe the roles and responsibilities of the actors in each process	
646	processes and activities	health processes	documentation	feature	The health organization develops a document that: a) describes the health and telehealth service processes; b) defines the responsibilities and delegations (agreements) of the health actors involved; c) defines responsibilities for maintaining processes, protocols and guidelines; d) defines the necessary SLA service level agreements with the supporting organizations (see Clause 8; e) defines the processes for mapping the information exchanged between the health actors. This must be a complete description of the process, including all the necessary parts to establish the telehealth service, as well as its relationships, both in terms of care activity and formally, with its legal aspects.	ISO13131, 2021 5.4.2,*105.1*
647	processes and activities	production	shares of teleconsultations	graphic	Graph 7. Teleconsultations performed by Telehealth Nucleus (maintenance), Brazil 20162018. Graph 8. Total Teleconsultations carried out by Telehealth Nucleus (maintenance), Brazil 20162018	HAOC2, 2019 4.2,*281.1*
648	processes and activities	production	shares of telediagnosis	table	Table 12. Telediagnosis actions, by Telehealth Centers (maintenance), Ministry of Health, Brazil 2016 2018. Type of Exams, 9, 7, 6, 12, 3, 4, 11, 8	HAOC2, 2019 4.2,*280.1*
649	processes and activities	production	roof	indicator	number of specialties in the remote unit / number of specialties in the reference unit	PAHO, 2016 , *185.1*
650	processes and activities	production	intention of forwarding of teleconsultations	graphic	Graph 9. Intention to refer teleconsultations, Brazil 20162018.	HAOC2, 2019 4.2,*282.1*
651	processes and activities	production	average of teleconsultations	indicator	The averages of teleconsultations defined in the items of the caput of this article are parameters for the initial phase of the Project's operation and will be periodically adjusted, in a specific act of the Ministry of Health, depending on the programming of the phases, the evolution and the general performance of the group of projects.<br>Note: Origin: p2554, 2011 art 20, $1	MSPC6, 2017 title II, cap II, section VII, art 142, $1,*355.1*
652	processes and activities	production	target for sending SOF to BIREME	indicator	Description: monthly average of teleconsultations candidates for SOF forwarded to BIREME by Nucleus; Unit: number of teleconsultations candidates for SOF forwarded to BIREME /month/nucleus; Goal: 2 TC sent to BIREME/ month/nucleus;	MSNT63, 2014 4,*327.1*
653	processes and activities	production	transferred patients	indicator	number of patients who needed to be transferred / number of patients transferred	PAHO, 2016 , *183.1*
654	processes and activities	production	BIREME response deadline	feature	BIREME has a period of 30 days, counting from the date of receipt of the SOF, to give the first feedback to the NT, about the review process.	MSNT63, 2014 5, II,*329.1*
655	processes and activities	production	remote professionals	indicator	number of professionals in the remote unit / number of professionals trained in the last year	PAHO, 2016 , *184.1*
656	processes and activities	production	transfer time	indicator	Transfer time to remote unit / Transfer time to reference unit	PAHO, 2016 , *186.1*
657	processes and activities	service design	documentation	feature	The healthcare organization included a telehealth service design document considering: a) a care recipient's ability to travel and their family, work, and cultural background when determining the telehealth services to be offered; b) the appropriate clinical goals and model(s) of care or shared care; c) the availability of specialists, local clinical staff and facilities necessary to provide continuity of telehealth services; d) the availability of specialists, local clinical staff and facilities necessary to provide continuity of telehealth services when face-to-face services are subsequently required as a result of the telehealth-based assessment; e) potential barriers (such as access to computers or telecommunications) to the inclusion of care recipients; f) that care recipients can choose which health services they consider appropriate to access, whether or not they are delivered using telehealth services.	ISO13131, 2021 8.1.2,*91.1*
658	processes and activities	clinical protocols unavailable	communication	feature	In cases where health care is provided using telehealth services in situations for which there are no guidelines or protocols, the healthcare organization ensures that the healthcare organization is informed	ISO13131, 2021 10.1.6,*82.1*

#	theme	category	aspect	nature	element(s)	ref
					by the healthcare professional who lacks a clinical guideline or protocol for this situation and the care recipient is informed that no clinical guideline or protocol exists for the current situation	
659	processes and activities	clinical protocols unavailable	alternative plan of Careful	feature	In cases where healthcare is provided using telehealth services in situations for which there are no defined guidelines or protocols, the healthcare organization establishes an alternative plan of care and healthcare activities are carried out under that plan.	ISO13131, 2021 10.1.6,*81.1*
660	processes and activities	quality	scheduling of telemedicine subsequent	indicator	Determines the average number of subsequent appointments generated based on the first appointment, per patient. Interpretation: Indicates that the patient's issues are being addressed. Formula: Number of subsequent telemedicine consultations per patient x 100/ Number of patients seen for the first time with telemedicine. Frequency: Monthly. Indicator Type. Quality. Observations: Must be related to the type of specialty provided.	PAHO, 2016 , *208.1*
661	processes and activities	quality	waiting time for teleconsultation	indicator	Refers to the time elapsed between scheduling an appointment and its completion. Interpretation: Determines the average patient waiting time. Evaluates the time the patient saves through teleconsultations in relation to normal care. An ex post comparison is required. Formula: Sum of the total number of hours elapsed between scheduling and carrying out the consultation (Duration of the consultation in hours)/Number of teleconsultations performed. Frequency: Weekly. Indicator Type. Quality	PAHO, 2016 , *209.1*
662	processes and activities	data quality	documentation of Law Suit	feature	The organization ensures decisions made when designing these processes are documented	ISO13131, 2021 14.1.7,*17.1*
663	processes and activities	data quality	qualification of information on telemedicine	feature	The doctor who uses telemedicine, aware of his legal responsibility, must assess whether the information received are qualified, within strict protocols of digital security and sufficient for the proposed purpose.	CFM2314, 2022 considerations, *1836.1*
664	processes and activities	financial resources	conditions for calculation of the variable component of cost to the federative entity	indicator	a) activity of active and participating teams, related to professionals who use telehealth services in the reference month; b) definition and agreement on lines of care and/or priority specialties; c) size of the Telehealth Center; d) total production of teleconsultations, per team and per team doctor, each month, which can be classified as: synchronous, asynchronous.	MSPC6, 2017 title II, cap II, section VII, art 155,*361.1*
665	processes and activities	financial resources	conditions for calculation of the variable component cost of teleconsultations	indicator	a) the performance of at least 1 (one) teleconsultation per month per team, except for the production described in item II; b) at least 1 (one) teleconsultation per month by the team physician related to the defined and agreed line of care or specialty;	MSPC6, 2017 title II, cap II, section VII, art 158,*365.1*
666	processes and activities	financial resources	conditions for financial incentive of the variable component regarding teams and active physicians	indicator	a) the ratio of the number of active participating teams to the total number of participating teams in the respective Telehealth Center; b) the ratio of the number of active participating physicians to the total number of participating physicians in the respective Telehealth Center;	MSPC6, 2017 title II, cap II, section VII, art 156,*362.1*
667	processes and activities	financial resources	conditions for receipt of parts of the variable component	indicator	a) has at least 20% (twenty percent) of active participating teams in the month; b) has at least 20% (twenty percent) of participating physicians active in the month; c) perform at least 1 (one) teleconsultation per month per team and perform at least 1 (one) teleconsultation per month per team physician related to the defined and agreed line of care or specialty;	MSPC6, 2017 title II, cap II, section VII, art 167,*367.1*
668	processes and activities	financial resources	conditions for receipt of variable component depending on the size of the nucleus	indicator	a) production of teleconsulting by participating team: - from 1 (one) to 1.9 (one point nine) teleconsultation per participating team per month: 60% (sixty percent) of "X"; - from 2 (two) to 2.9 (two point nine) teleconsultations per participating team per month: 80% (eighty percent) of "X"; - more than 3 (three) teleconsultations per participating team per month: 100% (one hundred percent) of "X"; b) production of teleconsultation by the physician of the team related to the line of care or specialty agreed in the month: - from 1 (one) to 1.9 (one point nine) teleconsultations per participating physician per month: 60% (sixty percent) of "X"; - from 2 (two) to	MSPC6, 2017 title II, cap II, section VII, art 169,*368.1*

#	theme	category	aspect	nature	element(s)	ref
					2.9 (two point nine) teleconsultations per participating physician per month: 80% (eighty percent) of "X"; - more than 3 (three) teleconsultations per participating physician per month: 100% (one hundred percent) of "X". $ The variable "X" is equivalent to 20% (twenty percent) of the value transferred to the Nucleus according to its size.	
669	processes and activities	financial resources	definition of team and active doctor	indicator	a) active participating team or active participating physician: team or professional who requested teleconsultation in the reference month for payment; b) participating team or participating physician: the team or professional with a history of requesting teleconsultation in the last 3 (three) months;	MSPC6, 2017 title II, cap II, section VII, art 156,$,*363.1*
670	processes and activities	financial resources	division of the incentive financial of variable component	indicator	a) for the activity of active and participating Primary Care teams: up to 40% (forty percent) of the total value of the variable component to be received; b) for defining and agreeing on lines of care and/or priority specialties: 20% (twenty percent) of the total value of the variable component to be received; c) for the total production of teleconsultations: up to 40% (forty percent) of the total value of the variable component to be received;	MSPC6, 2017 title II, cap II, section VII, art 166,*366.1*
671	processes and activities	human Resources	team quantity	indicator	Number of people involved in the project	DESD, 2021 , *1801.1*
672	processes and activities	human Resources, health staff	time management	feature	There is provision for provision of telehealth services outside the hours of face-to-face care.	ARGMNMM, 2020 IV.a.92,*634.1*
673	processes and activities	human Resources, health staff	telecommuting	feature	There are plans to provide telehealth services in the teleworking, remote modality.	ARGMNMM, 2020 IV.a.93,*635.1*
674	processes and activities	responsibility of receivers of care	assessment	recipient feature	The healthcare organization ensures that the healthcare professional regularly assesses whether the care and/or informal caregiver are still capable and motivated to carry out their tasks using telehealth services.	ISO13131, 2021 11.1.7,*69.1*
675	processes and activities	responsibility of receivers of care	commitment	feature	The healthcare organization ensures that the care recipient and/or informal caregiver commits to carrying out the tasks required in the care plan using telehealth services	ISO13131, 2021 11.1.7,*68.1*
676	processes and activities	results and assessment	avoidance of referrals from teleconsultations	indicator	Description: % teleconsultations answered in which there was an intention to refer a patient in which referrals were avoided; Numerator: number of requests per professional category with the intention of forwarding where there was a change in conduct; Denominator: total number of requests by professional category where there was an intention to be forwarded before the request; Unit: %; Source: request data;	MSNT5, 2014 annex I, frame 5,*412.1*
677	processes and activities	results and assessment	doubt resolution in teleconsultations	indicator	Description: % teleconsultations answered in which the doubt was resolved (yes, partially, no, I don't know); Source: request data;	MSNT5, 2014 annex I, frame 5,*413.1*
678	processes and activities	results and assessment	second opinion formative SOF produced and shipped to BIREME	indicator	Description: number of SOF produced, sent and approved by BIREME; Numerator: number of requests chosen by the teleconsultants as SOF candidates; Source: request data;	MSNT5, 2014 annex I, frame 5,*414.1*
679	processes and activities	results and assessment	teleconsultations asynchronous answered in less 72 hours	indicator	Description: % of asynchronous requests answered in less than 72 hours; Source: request data;	MSNT5, 2014 annex I, frame 5,*418.1*
680	processes and activities	results and assessment	frequent topics in teleconsultations	indicator	Description: list of the 10 most frequent topics in teleconsultation requests answered (using the CID and CIAP classification; Source: request data;	MSNT5, 2014 annex I, frame 5,*415.1*
681	processes and activities	results and assessment	average time of answer from teleconsultations	indicator	Description: Average response time for synchronous and asynchronous requests; Source: request data;	MSNT5, 2014 annex I, frame 5,*417.1*
682	processes and activities	security and quality of technology	understanding of information	feature	The relationship between the care recipient, the healthcare organization, and any health support organization must be defined in an SLA service level agreement that ensures that information provided to the care recipient is understandable by the care recipient and informal caregivers	ISO13131, 2021 13.1.2,*20.1*
683	processes and activities	security and quality of technology	use of service and devices	feature	The relationship between the care receiver, the organization and any health support organization shall be defined in an SLA service level agreement	ISO13131, 2021 13.1.2,*18.1*

#	theme	category	aspect	nature	qlueemiecnltuoi(isn)provider usage instructions and information ref	
					about the intended use of the device and telehealth services	
684	processes and activities	telehealth service	monitoring of patients	feature	Follow-up of patients after hospitalization.	ICT-health, 2021 Online services line offered to the patient and telehealth, *823.1*
685	processes and activities	telehealth service	counseling genetic	indicator	The telehealth center performs some type of remote genetic counseling.	UN2030, 2016 , *1830.1*
686	processes and activities	telehealth service	guidelines for offering teleconsulting	feature	I. Offer integrated teleconsulting with regulation in priority specialties, observing the specialties/specialized procedures with repressed demand (identified by prolonged waiting time), whose reasons for referral are sensitive to clinical actions in PC and their capacity to respond. a) management of the queue in the priority specialties, defined based on the local regional reality; b) support for the implementation of regulation protocols and clinical guidelines, in priority specialties, defined based on the local regional reality; c) specialties defined and agreed in CIB, according to Ordinance GM/MS no. 2859/2014.	MSNT50, 2015 8, *760.1*
687	processes and activities	telehealth service	guidelines for telediagnosis	feature	a) meet the needs of municipalities in areas of care gaps for diagnostic support; b) offer telediagnosis based on clinical and epidemiological criteria using protocols for ordering tests; c) monitor the activities carried out by the core and report monthly through SMART.	MSNT50, 2015 32, *766.1*
688	processes and activities	telehealth service	forwarding by chat bots	feature	Use chat bots to help identify symptoms and appropriately refer patients, according to the symptom.	ICT-health, 2021 Online services line offered to the patient and telehealth, *820.1*
689	processes and activities	telehealth service	phone interaction or video call	feature	Conduct interactions between patients and health professionals through telephone or video calls.	ICT-health, 2021 Online services line offered to the patient and telehealth, *819.1*
690	processes and activities	telehealth service	monitoring of patients	feature	Perform remote monitoring of patients.	ICT-health, 2021 Online services line offered to the patient and telehealth, *822.1*
691	processes and activities	telehealth service	teleconsultation response time asynchronous	indicator	It must be answered within 72 hours by the core consultants.	MSNT50, 2015 3.I, *753.1*
692	processes and activities	telehealth service	telediagnosis response time	indicator	The response time for reports should be a maximum of 72 hours for elective exams.	MSNT50, 2015 31, *765.1*
693	processes and activities	telehealth service	access qualification to services specialized	feature	Centers can qualify user access to specialized services through integration with Regulation Centers. The work carried out in conjunction with regulatory processes consists of assessing the queue of the most demanded specialties in each region and presenting a specific approach for the queue management. This approach consists of using regulation protocols and referrals, development of guidelines, shared definition of flows, teleconsultation to discuss queue cases in PC, in addition to offering tele-education activities aimed at the main difficulties identified in queue management.	MSNT50, 2015 7, *759.1*
694	processes and activities	telehealth service	tracking of patients	feature	Use people tracking tools potentially infected.	ICT-health, 2021 Online services line offered

#	theme	category	aspect	nature	element(s)	ref
						to the patient and telehealth, *821.1*
695	processes and activities	telehealth service	second opinion formative SOF	feature	Systematized response to questions arising from teleconsultations, and selected based on criteria of relevance and pertinence in relation to SUS guidelines, such responses are constructed based on a bibliographic review, on the best scientific and clinical evidence.	MSNT50, 2015 3.II, *754.1*
696	processes and activities	telehealth service	teleconsulting	feature	Teleconsultation: consultation/question and recorded answer to clarify doubts about management, conduct and clinical procedures, health actions and issues related to the work process, based on scientific evidence, but adapted to local and regional characteristics. It works in 2 ways: a) synchronous - teleconsultation performed in real time: i. teleconsulting carried out through chat, and synchronous tools such as web conferencing or video conference; ii. teleconsultation carried out by a toll-free 0800 telephone service. It works through a call center, which refers the requesting PC professional to core professionals with experience in PC or in other specialties and with experience in clinical performance. b) asynchronous - teleconsultation carried out through offline messages, which must be answered within 72 hours by the core consultants.	MSNT50, 2015 3.I, *752.1*
697	processes and activities	telehealth service	telediagnosis	feature	Diagnostic support service, where tests are performed in a specific location and sent for issuing a report through technologies of the information and communication. The report will be issued by a core expert.	MSNT50, 2015 3.III, *755.1*
698	processes and activities	services and applications	digital applications for priority areas of services	indicator	Digital health services and applications: Public health sector priorities are supported by digital health services and applications. Identify the percentage of priority areas out of all those indicated that are served by digital health. For example, you answered referring to 8 priority areas (adding the list and the free text field), of these only 4 have digital services and applications available 50%. Answer: 0100%	BDHI, 2021 18, *463.1*
699	processes and activities	services and applications	priority areas of services	feature	List of priorities published in the document of the National Health Plan PNS20162019 a) Outpatient care in the SUS basic and specialized production; b) Primary care; c) Attention to emergencies; d) Pharmaceutical assistance; e) Attention to specific populations; f) Hospital care in the SUS; g) Women's health; h) Health care for people with chronic diseases; i) Transplants; j) Immunizations; k) Mental health; l) Attention to the health of the indigenous population; m) Production complex and science, technology and innovation in health; n) Care for people with disabilities; o) Child health;	BDHI, 2021 17, *462.1*
700	processes and activities	services and applications	geolocated data	feature	GIS geographic information systems Data are geolocated to enable GIS mapping.	BDHI, 2021 19th, *465.1*
701	processes and activities	services and applications	modalities of medical telecare	feature	Art. 5 Telemedicine can be exercised in the following forms of medical telecare: I Teleconsultation; II Teleinterconsultation; III Telediagnosis; IV Telesurgery; V Telemonitoring or telesurveillance; VI Telescreening; VII Teleconsulting.	CFM2314, 2022 art 5, *1844.1*
702	processes and activities	synchronization and modality	type of communication	indicator	Type of monitoring performed: synchronous, asynchronous and remote monitoring of patients	ICT-health, 2021 Use of technologies of information and from the communication in the area of health: a telehealth in 2021, *840.1*
703	processes and activities	systems and services, patterns and interoperability	continuity of Careful	feature	Continuity of care: Clinical information from the patient are shared with other institutions of health for continuity of care.	IMDS, 2021 , *676.1*
704	processes and activities	systems and services, patterns and interoperability	clinical pharmacy	feature	Clinical Pharmacy: Medical prescriptions are reviewed by a clinical pharmacist with the support of a system computerized with alerts.	IMDS, 2021 , *666.1*

#	theme	category	aspect	nature	element(s)	ref
705	processes and activities	systems and services, patterns and interoperability	data governance	feature	Governance for the Use of Sharing Networks Data: Does the institution have formal policies on the use of data sharing networks.	IMDS, 2021 , 678.1
706	processes and activities	systems and services, patterns and interoperability	interoperability	feature	Significant Use of Sharing Networks Data: The institution significantly uses the data from a data sharing network.	IMDS, 2021 , 679.1
707	processes and activities	systems and services, patterns and interoperability	data record	feature	Level of Use of the Care Team: The care team uses information systems and technologies (electronic medical records, for example) in patient care and care.	IMDS, 2021 , 659.1
708	processes and activities	systems and services, patterns and interoperability	telehealth services	feature	Telemedicine: The institution has or uses telemedicine services.	IMDS, 2021 , 673.1
709	processes and activities	support for the improvement of health care	standardization of protocols	feature	Standardization of specialty protocols, in an integrated and digital way in health facilities, allowing the proper management of health care demands and regulatory mechanisms;	PNIIS, 2021 chapter II, section III, article 6, III, 793.1
710	processes and activities	technology service support	new management services	feature	The healthcare organization ensures that procedures are in place to maintain the continuity and reliability of the telehealth service through the introduction of new services.	ISO13131, 2021 13.1.3,26.1
711	processes and activities	technology	technological resources	feature	Technological resources used: audio, video, images, streaming media, online portals, sending messaging, mobile apps	ICT-health, 2021 Use of technologies of information and from the communication in the area of health: a telehealth in 2021,841.1
712	processes and activities	transfer international of data	transfer international data	feature	Art. 33. The international transfer of personal data is only permitted in the following cases: I to countries or international organizations that provide a degree of protection of personal data adequate to that provided for in this Law; II when the controller offers and proves guarantees of compliance with the principles, rights of the holder and the data protection regime provided for in this Law, in the form of: a) clauses specific contractual arrangements for a given transfer; b) standard contractual clauses; c) global corporate standards; d) regularly issued seals, certificates and codes of conduct; III when the transfer is necessary for international legal cooperation between public intelligence, investigation and prosecution bodies, in accordance with the instruments of international right; IV when the transfer is necessary for the protection of the life or physical safety of the holder or a third party; V when the national authority authorizes the transfer; VI when the transfer results in a commitment assumed in an international cooperation agreement; VII when the transfer is necessary for the execution of public policy or legal attribution of the public service, publicity being given pursuant to item I of the caput of art. 23 of this Law; VIII when the holder has provided his specific and highlighted consent for the transfer, with prior information on the international character of the operation, clearly distinguishing it from other purposes; or IX when necessary to meet the hypotheses provided for in items II, V and VI of art. 7 of this Law.	LGPD, 2018 cap V, art 33, 138.1
713	processes and activities	data processing by the government	sharing of data	feature	Art. 26. The shared use of personal data by Public Power must meet the specific purposes of implementing public policies and legal attribution by bodies and public entities, respecting the principles of protection of personal data listed in art. 6 of this Law. § 1 The Public Power is prohibited from transferring personal data contained in databases to which it has access to private entities, except: I in cases of decentralized execution of public activity that requires the transfer, exclusively for this specific and determined purpose, subject to the provisions of Law No. 12,527, of November 18, 2011 (Access to Information Law); III in cases where the data are accessible	LGPD, 2018 chapter IV, art 26, 134.1

#	theme	category	aspect	nature	element(s)	ref
					publicly, observing the provisions of this Law. IV when there is a legal provision or the transfer is supported by contracts, agreements or similar instruments; or Included by Law No. 13,853, of 2019 V - in the event that the transfer of data aims exclusively at preventing fraud and irregularities, or protect and safeguard the security and integrity of the data subject, provided that processing for other purposes is prohibited. § 2 The contracts and agreements mentioned in § 1 of this article must be communicated to the national authority.	
714	processes and activities	data processing by the government	consent of patient	feature	Art. 27. The communication or shared use of personal data from a legal entity governed by public law to a person governed by private law will be informed to the national authority and will depend on the consent of the holder, except: I in the cases of waiver of consent provided for in this Law; II in cases of shared use of data, in which publicity will be given under the terms of item I of the caput of art. 23 of this Law; or III in the exceptions contained in § 1 of art. 26 of this Law. Sole Paragraph. The information to the national authority referred to in the caput of this article will be subject to regulation.	LGPD, 2018 chapter IV, art 27, *135.1*
715	processes and activities	data processing access to information			Art. 9 The data subject has the right to facilitated access to information on the processing of his data, which must be made available in a clear, adequate and ostensive way about, among other characteristics provided for in regulations for complying with the principle of free access: I purpose treatment specific; II form and duration of treatment, observing commercial and industrial secrets; III identification of the controller; IV controller contact information; V information about the shared use of data by the controller and the purpose; VI Responsibilities of the agents who will carry out the processing; and VII holder's rights, with explicit mention of the rights contained in art. 18 of this Law. § 1 In the event that consent is required, this featurewill be considered void if the information provided to the holder have misleading or abusive content or have not been previously presented with transparency, in a clear and unequivocal way. § 2 In the event that consent is required, if there are changes in the purpose for processing personal data that are not compatible with the original consent, the controller must previously inform the holder about the changes in purpose, and the holder may revoke the consent, if disagree with the changes. § 3 When the processing of personal data is a condition for the provision of a product or service or for the exercise of a right, the holder will be informed with emphasis on this fact and on the means by which he will be able to exercise the rights of the holder listed in art. 18 of this Law.	LGPD, 2018 cap II, section I, art 9,*117.1*
716	processes and activities	data processing consent			Art. 8 The consent provided for in item I of art. 7 of this Law must be provided in writing or by another means that demonstrates the holder's expression of will. § 1 If consent is provided in writing, it must be included in a separate clause from the other contractual clauses. § 2 It is up to the controller to bear the burden of proof that consent was obtained in accordance with the provisions of this Law. § 3 It is forbidden to process personal data through a defect in consent. § 4 The consent must refer to certain purposes, and generic authorizations for the processing of personal data will be null and void. § 5 featureConsent may be revoked at any time. moment by express manifestation of the holder, by free and facilitated procedure, ratifying the treatments carried out under the protection of the previously expressed consent while there is no request for elimination, under the terms of item VI of the caput of art. 18 of this Law. § 6 In case of change of information referred to in items I, II, III or V of art. 9 of this Law, the controller must inform the holder, specifically highlighting the content of the amendments, and the holder may, in cases where his consent is required, revoke it if you disagree with the change.	LGPD, 2018 cap II, section I, art 8,*116.1*

#	theme	category	aspect	nature	element(s)	ref
717	processes and activities	data processing	data from children and adolescents	feature	Art. 14. The processing of personal data of children and adolescents must be carried out in their best interest, under the terms of this article and the relevant legislation. § 1 The processing of personal data of children must be carried out with the specific and prominent consent given by at least one of the parents or legal guardian. § 2 In the data processing referred to in § 1 of this article, controllers must keep public information about the types of data collected, the way in which they are used and the procedures for exercising the rights referred to in art. 18 of this Law. § 3 Personal data of children may be collected without the consent referred to in § 1 of this article when the collection is necessary to contact the parents or legal guardian, used only once and without storage, or for their protection, and in no case may they be passed on to a third party without the consent referred to in § 1 of this article. § 4 The controllers shall not condition the participation of the holders referred to in § 1 of this article in games, internet applications or other activities to the provision of personal information beyond what is strictly necessary for the activity. § 5 The controller must make all reasonable efforts to verify that the consent referred to in § 1 of this article was given by the person responsible for the child, considering the technologies available. § 6 The information on the processing of data referred to in this article must be provided in a simple, clear and accessible manner, considering the physical-motor, perceptive, sensory, intellectual and mental characteristics of the user, using audiovisual resources when appropriate, in a manner to provide the necessary information to the parents or legal guardian and adequate to the child's understanding.	LGPD, 2018 chapter II, section III, article 14, *122.1*
718	processes and activities	data processing sensitive data		feature	Art. 11. Processing of sensitive personal data it can only occur in the following hypotheses: I when the holder or his/her legal guardian consents, in a specific and highlighted way, for specific purposes; II without providing the holder's consent, in cases where it is indispensable for: a) compliance with a legal or regulatory obligation by the controller; b) shared treatment of data necessary for the execution, by the public administration, of public policies provided for in laws or regulations; c) conducting studies by a research body, ensuring, whenever possible, the anonymization of sensitive personal data; d) regular exercise of rights, including in contracts and in legal proceedings, administrative and arbitration, the latter under the terms of Law No. 9,307 of September 23, 1996 Arbitration Law); e) protection of life or physical safety of the holder or third party; f) health protection, exclusively, in procedures carried out by health professionals, health services or health authorities; or g) guarantee of prevention of fraud and security of the data subject, in the processes of identification and authentication of registration in electronic systems, safeguarding the rights mentioned in art. 9 of this Law and except in the event that the holder's fundamental rights and freedoms that require the protection of personal data prevail. § 1 The provisions of this article apply to any processing of personal data that reveals sensitive personal data and that may cause damage to the holder, except for the provisions of specific legislation. § 2 In cases of application of the provisions of items “a” and “b” of item II of the caput of this article by public bodies and entities, the said waiver of consent will be publicized, under the terms of item I of the caput of art. 23 of this Law. § 3 The communication or shared use of sensitive personal data between controllers with the aim of obtaining economic advantage may be subject to prohibition or regulation by the national authority, after hearing the sectorial bodies of the Public Power, within the scope of their Skills. § 4 Communication or shared use between § 3 The communication or shared use of sensitive personal data between controllers with the aim of obtaining economic advantage may be subject to prohibition or regulation by the national authority, after hearing the sectorial bodies of the Public Power, within the scope of their competences. § 4 Communication or shared use between § 3 The communication or shared use of sensitive personal data between controllers with the aim of obtaining economic advantage may be subject to prohibition or regulation by the national authority, after hearing the sectorial bodies of the Public Power, within the scope of their competences. § 4 Communication or shared use between controllers of sensitive personal data relating to health in order to obtain economic advantage,	LGPD, 2018 cap II, section II, art 11, *119.1*

#	theme	category	aspect	nature	element(s)	ref
					except in the hypotheses related to the provision of health services, pharmaceutical assistance and health assistance, provided that § 5 of this article is observed, including auxiliary diagnosis and therapy services, for the benefit of the interests of the data subjects, and to allow : I data portability when requested by the data subject; or II the financial and administrative transactions resulting from the use and provision of the services referred to in this paragraph. § 5 Private health care plan operators are prohibited from processing health data for the practice of risk selection in the contracting of any modality, as well as in the contracting and exclusion of beneficiaries.	
719	processes and activities	data processing data processing		feature	Art. 7 The processing of personal data can only be carried out in the following cases: I through the provision of consent by the data subject; II for compliance with a legal or regulatory obligation by the controller; III by the public administration, for the treatment and shared use of data necessary for the execution of public policies provided for in laws and regulations or supported by contracts, agreements or similar instruments, subject to the provisions of Chapter IV of this Law; IV for carrying out studies by research body, ensuring, whenever possible, the anonymization of personal data; V when necessary for the execution of a contract or preliminary procedures related to a contract to which the data subject is a party, at the request of the data subject; VI for the regular exercise of rights in legal proceedings, administrative or arbitration, the latter under the terms of Law No. 9,307 of September 23, 1996 Arbitration Law); VII for the protection of life or physical safety of the holder or third party; VIII for the protection of health,<br><br>exclusively, in a procedure performed by health professionals, health services or health authorities; Wording given by Law No. 13,853, of 2019 Effectiveness IX when necessary to meet the legitimate interests of the controller or a third party, except in the case of prevailing fundamental rights and freedoms of the holder that require the protection of personal data; or X for credit protection, including the provisions of the relevant legislation. § 3 The processing of personal data whose access is public must consider the purpose, good faith and public interest that justified its availability. § 4 The requirement of consent provided for in the caput of this article is waived for data made manifestly public by the holder, safeguarding the rights of the holder and the principles provided for in this Law. § 5 The controller who obtained the consent referred to in item I of the caput of this article who needs to communicate or share personal data with other controllers must obtain specific consent from the holder for this purpose, except for the cases of waiver of consent provided for in this Law. Any waiver of the consent requirement does not release the treatment agents from the other obligations set forth in this Law, especially the observance of the general principles and the guarantee of the data subject's rights. § 7 The subsequent processing of personal data referred to in §§ 3 and 4 of this article may be carried out for new purposes, provided that the legitimate and specific purposes for the new treatment and the preservation of the holder's rights are observed, as well as the foundations and principles set forth in this Law.	LGPD, 2018 cap II, section I, art 7, *115.1*
720	protection and security	legal aspects and ethical	digital signature qualified	feature	To act by telemedicine, the physician must have qualified digital signature, ICPBrasil standard, in terms of the laws in force in the country.	CFM2314, 2022 considerations, *1837.1*
721	protection and security	legal aspects and ethical	security and reliability	feature	The solution guarantees the security and confidentiality of patients' health information.<br>Answer: yes / no / not applicable / don't know<br>Note: Does not mention data from health professionals.	HAOC1, 2019 3, *228.1*
722	protection and security	confidentiality	confidentiality of health records	feature	Does the organization implement processes to ensure that the health professional applies the guidelines of the	ISO13131, 2021 14.1.4, *8.1*

#	theme	category	aspect	nature	element(s)	ref
					health organization to protect the confidentiality of health records	
723	protection and security	ethics, privacy and confidentiality	rules for recording in the medical record	feature	Art. 3rd In the services provided by telemedicine, the data and images of the patients, contained in the medical records must be preserved, obeying the legal norms and those of the CFM pertinent to the custody, handling, integrity, veracity, confidentiality, privacy, irrefutability and guarantee of professional secrecy of information. Art. 3º § 7º The personal and clinical data of medical telecare must follow the definitions of the LGPD and other legal provisions, regarding the primary purposes of the data.	CFM2314, 2022 art 3,*1839.1*
724	protection and security	ethics, privacy and confidentiality	data protection	feature	Develop mechanisms and structures that ensure the protection of data or information defined as those that cannot be made available or disclosed without the express authorization of the individual to persons, entities or processes.	ABRASCO, 2020 4th dimension, *526.1*
725	protection and security	ethics, privacy and confidentiality	data security	feature	Develop mechanisms and structures through computing criteria and procedures to ensure the security of records in the SIS (eg signature digital; bus; blockchain; audit trails, among others).	ABRASCO, 2020 4th dimension, *527.1*
726	protection and security	governance and management	data access control	feature	Establish means for the effective use of authorized access control mechanisms to personal data by the different user profiles: user, professionals of health, health care and surveillance managers, research bodies and legally authorized public agents, in accordance with Law No. 13,709, of August 14, 2018.	PNIIS, 2021 cap II, section I, art 4, VIII,*784.1*
727	protection and security	governance and management of information and technology of health information	quality warranty of information and technology	feature	Implement actions that guarantee the quality of health information and the technologies adopted. A guarantee of this quality – authenticity and chain of uninterrupted custody – constitutes one of the non-delegable functions of the public manager within the scope of the SUS.	ABRASCO, 2020 1st dimension, 6,*505.1*
728	protection and security	governance and management of information and technology of health information	quality of information	feature	Health information is a citizen's right and information management must guarantee the quality of health information for decision-making by managers, fulfilling institutional responsibilities assigned since the generation of the health record, covering the entire chain of custody of the document/record.	ABRASCO, 2020 1st dimension, 9,*508.1*
729	protection and security	governance and leadership for ESD	activities to define and develop initiatives aligned with the LGPD	feature	a) identification of the critical points of alignment with the LGPD for the expansion of the RNDS; b) identification of health data sharing models aligned with the LGPD; c) proposition of robust models of informed and informed consent of easy understanding, implementation and adherence by the patient, aligned with the precepts of the LGPD; d) proposition of authentication, security, secrecy and privacy models in line with the LGPD.	ESD28, 2020 1.3.1,*700.1*
730	protection and security	infrastructure	data security	feature	Information Security: The institution has policies for information security with regular training and technologies that guarantee the privacy and confidentiality of information.	IMDS, 2021 , *681.1*
731	protection and security	legislation, policy and norms/regulations	regulation of services	feature	Protocol for regulating or certifying digital health devices or services: There are accepted protocols, policies, frameworks, or processes that govern clinical patient care and use of medical devices and digital health services (e.g., telemedicine), particularly in relation to data security and integrity, as well as the quality of care services.	BDHI, 2021 7,*445.1*
732	protection and security	patterns and interoperability	impact of standards on the quality of health care	feature	Standards have a positive impact on the security of the patient, care, completeness and quality of health care.	BDHI, 2021 15c,*459.1*
733	protection and security	preparation organizational	privacy and security	feature	Medical staff are aware of privacy practices and security based on ethical and legal principles current.	ARGMNMM, 2020 I.29,*571.1*
734	protection and security	preparation organizational	privacy and security	feature	The non-medical team knows the practices of privacy and security based on ethical principles and applicable laws.	ARGMNMM, 2020 I.30,*572.1*
735	protection and security	privacy	audit of conformity of privacy	feature	The organization defines processes to audit the compliance of healthcare professionals and supporting organizations with health care regulations	ISO13131, 2021 14.1.2,*5.1*

#	theme	category	aspect	nature	[illegible]	ref
					e) the health organization and with any national regulation or legislation that may apply to telehealth services	
736	protection and security	privacy	consent on the regulations of privacy	feature	The organization shall notify the care recipient and obtain agreement to the care regulations. privacy of the organization providing the services of telehealth, including possible updates and/or changes to regulations	ISO13131, 2021 14.1.2,*3.1*
737	protection and security	process	consent of patient	feature	There are formal procedures for obtaining patients' informed consent remote.	ARGMNMM, 2020 I.51,*593.1*
738	protection and security	process	patient safety	feature	There are defined processes to address the patient safety considerations and institutional legal responsibility.	ARGMNMM, 2020 I.46,*588.1*
739	protection and security	process	data security	feature	There are defined processes to ensure the security, confidentiality and copying of data and information generated during telehealth services.	ARGMNMM, 2020 I.47,*589.1*
740	protection and security	protection from identity	confirmation of identity	feature	The organization protects the safety of individual health care workers and the confidentiality of health records by implementing processes that confirm the identity of the care recipient to the health professional and that confirm the identity of the health care professional to the care recipient	ISO13131, 2021 14.1.3,*6.1*
741	protection and security	security of information	risk management	feature	Art. 48. The controller must notify the national authority and the holder of the occurrence of a security incident that may pose a risk or relevant damage to the holders. § 1 The communication will be made within a reasonable time, as defined by the national authority, and must mention, at least: I the description of the nature of the personal data affected; II information on the holders involved; III the indication of the technical and security measures used to protect the data, observing commercial and industrial secrets; IV the risks related to the incident; V the reasons for the delay, in case the communication was not immediate; and VI the measures that have been or will be adopted to reverse or mitigate the effects of the loss. § 2 The national authority will verify the seriousness of the incident and may, if necessary to safeguard the rights of holders, order the controller to adopt measures, such as: I wide dissemination of the fact in the media; and II measures to reverse or mitigate the effects of the incident. § 3 In the judgment of the seriousness of the incident, any evidence that adequate technical measures have been adopted that make the affected personal data unintelligible, within the scope and technical limits of its services, for third parties not authorized to access them, will be evaluated.	LGPD, 2018 chap VII, section I, article 48,*150.1*
742	protection and security	security of information	data protection	feature	Art. 46. Processing agents must adopt security, technical and administrative measures capable of protecting personal data from unauthorized access and accidental or unlawful situations of destruction, loss, alteration, communication or any form of inappropriate or unlawful treatment. § 1 The national authority may provide for minimum technical standards to make applicable the provisions of caput of this article, considering the nature of the information processed, the specific characteristics of the treatment and the current state of technology, especially in the case of sensitive personal data, as well as the principles set out in the caput of art. 6 of this Law. § 2 The measures referred to in the caput of this article must be observed from the conception phase of the product or service until its execution.	LGPD, 2018 chap VII, section I, article 46,*148.1*
743	protection and security	security of information	data processing	feature	Art. 47. Treatment agents or any other person who intervenes in one of the treatment phases undertakes to guarantee the security of the information provided for in this Law in relation to personal data, even after its termination.	LGPD, 2018 chap VII, section I, article 47,*149.1*
744	protection and security	technology	security	feature	The solution is safe (harm risks less than benefits). Answer: yes / no / not applicable / don't know	HAOC1, 2019 two,*223.1*
745	protection and security	transfer international of	data protection	feature	Art. 34. The country's level of data protection abroad or the international organization mentioned	LGPD, 2018 cap V, art 34,

#	theme	category	aspect	nature	element(s)	ref
		data			in item I of the heading of art. 33 of this Law will be evaluated by the national authority, which will take into account: I - the general and sectorial norms of the legislation in force in the country of destination or in the international organization; II the nature of the data; III the observance of the general principles of protection of personal data and rights of holders provided for in this Law; IV the adoption of security measures provided for in regulations; V the existence of judicial and institutional guarantees for the respect of personal data protection rights; and VI other specific circumstances relating to the transfer.	139.1
746	protection and security	data processing by the government	risk management		Art. 32. The national authority may request Public Power agents to publish impact reports featureto the protection of personal data and suggest the adoption of standards and good practices for the processing of personal data by the Government.	LGPD, 2018 chapter IV, art 32, 137.1
747	protection and security	user like protagonist	data protection culture		Promoting a culture of data protection and security feature of information between professionals, managers and users of the health system.	PNIIS, 2021 chapter II, section IV, art 7, VII, 797.1
748	resources humans	education and training permanent of teams of information and technology	continuing education	feature	Review the profiles of the professionals that make up the ITIS teams, incorporating updating modalities in view of the accelerated innovation process in this field.	ABRASCO, 2020 3rd dimension, 3,521.1
749	resources humans	education and training permanent of teams of information and technology	career path specific to technology	feature	Establish the incorporation of ITIS professionals with specific training at all levels of the organization of health services, and that recognizes the specificities of their knowledge, skills and skills, as well as the urgent need to incorporate technicians with high professional qualifications through a public tender.	ABRASCO, 2020 3rd dimension, 5,523.1
750	resources humans	team	formation of medical teleconsultant		a) generalist; b) specialization in Family and Community Medicine MFC; c) specialization in clinical featuredoctor; d) specialization in dermatology; It is) specialization in pediatrics; f) specialization in gynecology; g) other specializations;	HAOC3, 2019 section 4.4,305.1
751	resources humans	team	binding of teleconsultant	feature	a) to the nucleus; b) to the municipality; c) to the state; d) a partner (health department or school, etc.);	HAOC3, 2019 section 4.4,304.1
752	resources humans	structure	training	indicator	Description: number of professionals who were qualified to use telehealth tools; Numerator: number of professionals; Unit: professionals; Source: municipal data; Period: data must be updated monthly, however, every 6 months (March and September) a general update of the registration database of all centers must be carried out for general cleaning of the database, in relation to the points of teams and registered people .<br><br>Note: Source: Telehealth Manual for Primary Care, MinSaúde, UFRGS 2012, adapted by the MinSaúde technical team.	MSNT5, 2014 annex I, frame 1,376.1
753	resources humans	structure	human Resources	feature	Human resources: institutional capacity in the areas of health services and ICTs.	ARGMNMM, 2020 1.4.1,536.1
754	resources humans	governance and management of information and technology of health information	training of teams		The decision-making cycle for the adoption of a standard and/or norm requires analysis of the viability and feasibility of training processes for health teams feature users of SIS and digital health services, who will use the new standards/norms. In this case, partnership with the network of health education institutions becomes strategic and necessary.	ABRASCO, 2020 1st dimension, 16,514.1
755	resources humans	skills and training of team	ICT skills	feature	The healthcare organization ensures that it specifies the key skills and competencies required by the personnel involved in the management of information and communication ICT in the health organization or support organization	ISO13131, 2021 9.1.2,89.1
756	resources humans	skills and training of team	infrastructure	feature	The healthcare organization ensures that there is sufficient knowledge of the local environment, health threats and clinical infrastructure at the point of care	ISO13131, 2021 9.1.2,87.1
757	resources humans	skills and training of team	provision of healthcare	feature	The healthcare organization ensures that the healthcare professional health and/or informal caregivers have the necessary skills to provide health care through telehealth services, including	ISO13131, 2021 9.1.2,85.1

#	theme	category	aspect	nature	element(s)	ref
					specific clinical, cultural, communication or language skills	
758	resources humans	skills and training of team	provision of healthcare	feature	The healthcare organization ensures that appropriate minimum professional standards in accordance with the jurisdiction of all stakeholders are applied in assessing the competencies needed to deliver healthcare through telehealth services.	ISO13131, 2021 9.1.2,*86.1*
759	resources humans	preparation organizational	team training computer science	feature	If you have a computer team, the professionals are qualified to provide support services to the telehealth.	ARGMNMM, 2020 I.5,*542.1*
760	resources humans	preparation organizational	training in telehealth	feature	Options for training and capacity building in telehealth are established.	ARGMNMM, 2020 I.20,*557.1*
761	resources humans	preparation organizational	medical team capable	feature	The medical team is qualified to provide telehealth services.	ARGMNMM, 2020 I.18,*555.1*
762	resources humans	preparation organizational	non-medical staff capable	feature	The non-medical team is qualified to provide telehealth services.	ARGMNMM, 2020 I.19,*556.1*
763	resources humans	preparation organizational	support staff	feature	There are support staff.	ARGMNMM, 2020 I.21e,*563.1*
764	resources humans	service quality	diversity of experts	indicator	Number and diversity of specialists since the creation of the program	PAHO, 2016 , *190.1*
765	resources humans	human Resources	recruitment of teleconsultants	indicator	team assembly	DESD, 2021 , *1815.1*
766	resources humans	human Resources	hired team	indicator	team hiring	DESD, 2021 , *1811.1*
767	resources humans	human Resources, health staff	health team	feature	There are health personnel available to participate in a telehealth program.	ARGMNMM, 2020 IV.a.87,*629.1*
768	resources humans	human Resources, health staff	health team	feature	There is a clear determination of the profile of the personnel working in telehealth and specialty services.	ARGMNMM, 2020 IV.a.88,*630.1*
769	resources humans	human Resources, health staff	telehealth team	feature	There is a determination of the minimum capacities of the people who work in the different services of telehealth.	ARGMNMM, 2020 IV.a.89,*631.1*
770	resources humans	human Resources, you guys	telehealth team	feature	There is clarity about the profile of people who attend telehealth services.	ARGMNMM, 2020 IV.b.96,*637.1*
771	resources humans	human Resources, you guys	technical team	feature	The institution has its own staff with IT expertise that supports IT services telehealth.	ARGMNMM, 2020 IV.b.95,*636.1*
772	resources humans	human Resources, you guys	technical team	feature	There are trained personnel to carry out an analysis criticism of any equipment and technologies of the information that must be acquired.	ARGMNMM, 2020 IV.b.97,*638.1*
773	resources humans	human Resources, you guys	technical team	feature	Are there personnel with the necessary experience to negotiate with suppliers the purchase and contracting of information technologies and support services.	ARGMNMM, 2020 IV.b.98,*639.1*
774	resources humans	results and assessment	professions and specialtiesmore frequent in teleconsultations	indicator	Description: professions and specialties of the most frequent teleconsultants among the teleconsultation requests answered; Source: request data;	MSNT5, 2014 annex I, frame 5,*416.1*
775	resources humans	telehealth service	telephone team education	feature	I. contentist; II. learning facilitator; III. tutor;	MSNT50, 2015 15,*761.1*
776	resources humans	telehealth service	team of teleconsulting	feature	I. teleregulator; II. teleconsultant; III. field coordinator; IV. field monitor;	MSNT50, 2015 5,*758.1*
777	networking, innovation and search	environment of interconnectivity	activities for develop data analysis initiatives	feature	Provision of data stored in the RNDS in anonymized form for analysis and research, observing and safeguarding the confidentiality of personal health information, through the rights to data protection and privacy, in line with the Open Data Plan of the Ministry of Health, with Law No. 13,709, of August 14, 2018, and with Law No. 12,527, of November 18, 2011.	PNIIS, 2021 chapter II, section VI, art 9, VI, *807.1*
778	networking, innovation and search	environment of interconnectivity	activities for develop initiatives	feature	Using big data in healthcare to provide evidence for policy, research and planning so that	PNIIS, 2021 chapter II, section

#	theme	category	aspect	nature	element(s)	ref
			embigdata		discoveries in digital health translate into action.	VI, art 9, III, *805.1*
779	networking, innovation and search	environment of interconnectivity	activities for develop initiatives in artificial intelligence	managers, service providers and	Promoting the dissemination of health data and information and the use of artificial intelligence in order to meet both the needs of users, featureprofessionals, of social control, regarding the exchange needs with training, teaching and research institutions, among others.	PNIIS, 2021 chapter II, section VI, art 9, IV, *806.1*
780	networking, innovation and search	environment of interconnectivity	collaboration network	so that technologies, concepts, standards,	Promoting collaborative and innovative work leveraged by the RNDS in all sectors of featurehealth service models, policies and regulations are put in place.	PNIIS, 2021 chapter II, section VI, article 9, I,*803.1*
781	networking, innovation and search	ecosystem of innovation	intersectoral articulation for the production of software	of featurequalify the software production capacity, in the	Promotion of intersectoral articulation with the objective health care interest	PNIIS, 2021 chapter II, section VII, art 10, III, *810.1*
782	networking, innovation and search	ecosystem of innovation	activities for develop health surveillance initiatives	and the creation of	a) receipt and integration of notifications and results of laboratory tests related to Covid-19; b) sending the test result to the citizen himself and to the health professionals authorized by him through the Connect SUS Portal; c) the action also includes the definition of clinical information models, the featuredefinition of common terminologies messages that allow interoperability between different health information systems; d) the integrated services of the RNDS must be expanded to include actions of immunization programs, registration of cases of notifiable diseases and health surveillance.	ESD28, 2020 7.1.2,*738.1*
783	networking, innovation and search	ecosystem of innovation	activities for develop initiatives in Iot, bigdata and secondary use of data	feature	a) attract public and private health organizations to, together with all actors, identify priority scenarios for the use of these advanced technologies; b) attract actors already involved in Big Data and IoT efforts in Health, to share knowledge, propose and execute the essential set of initiatives, using the RNDS as a structural element, as a testing ground for the initiatives and for their use in production , when possible; c) analyze the results obtained at each cycle, disseminating the knowledge obtained and defining new initiatives.	ESD28, 2020 7.2.1,*741.1*
784	networking, innovation and search	ecosystem of innovation	activities to explore health value models	featureRNDS framework for testing and evaluating models of	a) identify and attract actors who have experience and interest in the topic, to propose the initial range of initiatives; b) use the Collaboration Space and the value analysis, focusing on the various players in the health system; c) disseminate evaluation results and expand models as needed.	ESD28, 2020 7.3.1,*743.1*
785	networking, innovation and search	ecosystem of innovation	activities for deploy health data lake	line with other ESD actions, especially those of	a) develop the governance of the generation and use of public and private data; b) establish legal and ethical criteria for the use of data, respecting the LGPD, featurein Priority 1; c) plan and execute the essential set of initiatives that enable the coordinated use of the Data Lake.	ESD28, 2020 7.2.2,*742.1*
786	networking, innovation and search	ecosystem of innovation	activities for implement services electronic prescription	feature	a) articulation with the Federal Councils of Pharmacy, Dentistry and Medicine, in addition to health service providers to expand sponsorship and engagement of the professionals involved; b) definition of clinical information models and creation of FHIR messages that allow interoperability between different information systems; c) definition of common terminologies that promote the development of a National Medicines Base.	ESD28, 2020 7.1.3,*739.1*
787	networking, innovation and search	ecosystem of innovation	activities for implement services of regulation	featurea) articulation with municipal and state managers,	as well as providers and payers of health system services; b) alignment of roles and responsibilities; c) definition of clinical information models and common terminologies; d) creation of data exchange messages that allow interoperability and integrated management of care networks; e) detailing the scope of ambitions, limits, potentialities and challenges arising from the implementation of an intelligent model	ESD28, 2020 7.1.4,*740.1*

#	theme	category	aspect	nature	element(s)	ref
					regulation support; f) potential volume of data for the RNDS with an inventory of beds related to Covid-19 and other infectious diseases, which has been done since April 2020.	
788	networking, innovation and search	ecosystem of innovation	activities to support the incorporation of innovations	feature	a) attract and engage relevant players in health and social development; b) propose and implement integrated models of cooperation for financing innovations in Digital Health; c) induce industries, technology sectors, the productive system as a whole to strengthen a productive and innovative complex, in Digital Health, aimed at the strategic needs of health and social development.	ESD28, 2020 7.4.1,*744.1*
789	networking, innovation and search	ecosystem of innovation	activities for promote support for assistance contact	feature	a) The RNDS will allow public and private establishments to send information collected in care contacts, such as consultations, hospitalizations and vaccination, so that it is stored and made available in accordance with confidentiality and security standards proportionate to their sensitivity. b) The challenge of sharing care contact data includes the definition of clinical models, common terminologies and the creation of messages via Fast Healthcare Interoperability Resources FHIR, a widely accepted international standard, which allow interoperability between different health information systems . "Care Contact" has already been defined as the health care attendance record information standard as part of the CMD Minimum Data Set.	ESD28, 2020 7.1.1,*737.1*
790	networking, innovation and search	ecosystem of innovation	activities to use search features translational	feature	a) develop, implement and exercise organizational models and the necessary resources to enable Translational Research in Digital Health; B) systematically assess the adequacy of models proposals and act to correct, update or change them to capture new values or adapt them to new needs.	ESD28, 2020 7.4.2,*745.1*
791	networking, innovation and search	ecosystem of innovation	training for the use of health information	feature	Encouraging the development of methodologies and scientific and technological tools for the management, qualification and use of health information.	PNIIS, 2021 chapter II, section VII, art 10, IV, *811.1*
792	networking, innovation and search	ecosystem of innovation	connectivity	feature	Encouraging the maximum use of the RNDS as a connectivity environment in health, as an open innovation laboratory, in order to create a safe environment for testing and scaling new solutions, taking advantage of technologies created by startups and private health institutions, whose mastery becomes if public.	PNIIS, 2021 chapter II, section VII, art 10, I, *808.1*
793	networking, innovation and search	ecosystem of innovation	stimulate ecosystem of digital health	feature	Encouraging partnerships between the public and private sectors, universities, scientific societies, regulatory agencies and other ministries, to promote sustainable financing models, leverage the development and use of technologies and innovation in digital health solutions.	PNIIS, 2021 chapter II, section VII, art 10, IX, *816.1*
794	networking, innovation and search	ecosystem of innovation	stimulate ecosystem international health digital	feature	Encouraging the exchange of international experiences and cooperation to develop strategies and digital health solutions across countries.	PNIIS, 2021 chapter II, section VII, art 10, X, *817.1*
795	networking, innovation and search	ecosystem of innovation	action plan	feature	7. Ensure that there is an Innovation Ecosystem that takes full advantage of the Health Interconnectivity Environment, establishing itself as a large open innovation laboratory, subject to the guidelines, norms and policies established through priority 1.	ESD28, 2020 7,*735.1*
796	networking, innovation and search	ecosystem of innovation	priorities	feature	7. Innovation ecosystem 7.1 Expansion of RNDS integrated services 7.1.1 Promote support to care contact 7.1.2 Develop health surveillance initiatives 7.1.3 Implement electronic prescription services 7.1.4 Implement regulatory services 7.2 Distributed innovation ecosystem 7.2.1 Develop initiatives in IoT, big data and secondary use of data 7.2.2 Deploy the health information data lake 7.3 Value-based healthcare 7.3.1 Explore value models in healthcare 7.4 Evaluate and incorporate new technologies 7.4.1 Support the incorporation of innovations 7.4.2 Utilize translational research capabilities	ESD28, 2020 7,*736.1*
797	networking, innovation and search	ecosystem of innovation	digital solutions aligned with policies	feature	Guidance so that the production of software and other digital health solutions seek to: a) develop	PNIIS, 2021 chapter II, section

#	theme	category	aspect	nature	element(s)	ref
			national		technology in line with PNIIS, ESD and RNDS; b) include in the products the connection with the interfaces made available by the system managers without burdening the health service provider; c) train its employees in the technologies and standards defined by SUS managers; and d) adopt technologies and standards determined by SUS managers;	VII, art 10, XI, *818.1*
798	networking, innovation and search	governance and management of information and technology of health information	collaboration and mastery public in technologies	feature	This accountability of ITIS management presupposes the production of strategic innovations for health praxis, using universal public and open domain technologies throughout its development cycle, in partnership with national public teaching and research institutions in a collaborative work , constituting a Network of Collaborating Centres.	ABRASCO, 2020 1st dimension, 11,*510.1*
799	networking, innovation and search	governance and leadership for ESD	activities for establish the regulation of environments of innovation and interconnectivity	feature	a) identify and attract relevant actors; b) establish the legal and organizational framework for regulating collaboration; c) implement, evaluate and continually improve regulatory processes.	ESD28, 2020 1.3.2,*701.1*
800	networking, innovation and search	governance and resources organizational	tecnologic innovation	feature	Innovation and startups: There is a strategy for process innovation and incorporation of new technologies.	IMDS, 2021 , *690.1*
801	networking, innovation and search	Research and development	formalization of the scientific and innovation project	indicator	Formalization of the scientific and innovation project	DESD, 2021 , *1812.1*
802	networking, innovation and search	search, development, innovation in information and technology	specific support for priorities	feature	Establishment of research programs induced in the field of ITIS by expanding consultations with research centers on its formulation and the necessary resources to its sustained and sufficient financing, through of specific notices that address, as a priority, problems/ gaps of greater complexity and national relevance.	ABRASCO, 2020 2nd dimension, two,*517.1*
803	networking, innovation and search	search, development, innovation in information and technology	promotion for studies of evaluation	feature	Foster periodic evaluation studies of the national production of S&T in ITIS, also through calls publications from research funding agencies, such as CAPES, CNPq and Finep, among others.	ABRASCO, 2020 2nd dimension, 3,*518.1*
804	networking, innovation and search	search, development, innovation in information and technology	collaboration network	feature	It is recommended to set up a Network of Collaborating Centers for Research, Teaching and Innovation in Information and Information Technology in Health aimed at an intensive process of interlocutions and exchange of experiences that leverage investments to promote R&D and the financing of initiatives, constituting a critical, creative, knowledge-producing mass.	ABRASCO, 2020 2nd dimension, 1,*516.1*
805	networking, innovation and search	support for the improvement of health care	monitoring by wearable devices	Indicator	Carrying out a cost-benefit analysis for the adoption of remote monitoring of the health conditions of chronic patients through smart wearable devices.	PNIIS, 2021 chapter II, section III, art 6, V,*794.1*
806	networking, innovation and search	technical support	research and evaluation	feature	The healthcare organization provides technical support for telehealth services that include research and assessment	ISO13131, 2021 13.1.8,*49.1*
807	networking, innovation and search	technology	pilot project	feature	A pilot project is carried out for implementation. Answer: yes / no / not applicable / don't know	HAOC1, 2019 two,*227.1*
808	networking, innovation and search	data processing	use of sensitive data for research	feature	Art. 13. When carrying out studies in public health, the research bodies may have access to personal databases, which will be treated exclusively within the body and strictly for the purpose of carrying out studies and research and kept in a controlled and safe environment, in accordance with security practices provided for in specific regulation and which include, whenever possible, the anonymization or pseudonymization of data, as well as considering due ethical standards related to studies and research. § 1 The disclosure of the results or any excerpt from the study or research referred to in the caput of this article may under no circumstances reveal personal data. § 2 The research body will be responsible for the security of the information provided for in the caput of this article, not allowing, under any circumstances, the transfer of data to a third party. § 3 Access to the data referred to in this article will be subject to regulation by the national authority and the health and sanitary authorities, within the scope of their competences. § 4 For the purposes	LGPD, 2018 cap II, section II, art 13,*121.1*

#	theme	category	aspect	nature	element(s)	ref
					of this article, pseudonymization is the treatment whereby data loses the possibility of association, directly or indirectly, with an individual, if not through the use of additional information kept separately by the controller in a controlled and safe environment.	
809	networking, innovation and search	user like protagonist	partnerships for patient education	feature	Encouraging partnerships with undergraduate and graduate courses in the health area with a view to education permanent group of young people and adults on digital health and technologies for access to monitoring in individual and collective health	PNIIS, 2021 chapter II, section IV, art 7, VI, 796.1
810	training and disclosure	regulatory aspects	continuing education	feature	Is there a process to keep staff up to date that provides telehealth services under the norms applicable laws.	ARGMNMM, 2020 V.103, 643.1
811	training and disclosure	disclosure	event of dissemination/training	indicator	Outreach/training event	DESD, 2021 , 1821.1
812	training and disclosure	education	workload of education	indicator	hours in educational modules/courses	DESD, 2021 , 1819.1
813	training and disclosure	education	formation of professional	indicator	Number of trained professionals	DESD, 2021 , 1793.1
814	training and disclosure	education and training permanent of teams of information and technology	collective construction of curricular contents	feature	The collective construction of curricular contents that provide new skills and competences to professionals from the ITIS teams and establishment of new ways of exercising professional practices and new instruments of health management.	ABRASCO, 2020 3rd dimension, 4, 522.1
815	training and disclosure	education and training permanent of teams of information and technology	mapping of courses	feature	Carry out a diagnosis of ITIS courses in Brazil in order to support strategies to increase the number of courses offered at the technical level, lato and stricto sensu postgraduate courses and to value ITIS praxis both within public health teaching institutions and in health services, in the three spheres of government, promoting the interdisciplinarity inherent in the field.	ABRASCO, 2020 3rd dimension, 1, 518.1
816	training and disclosure	education and training permanent of teams of information and technology	teaching networks	feature	Promote the use of teaching networks for the formation of a group of ITIS Teaching Centers with national coverage, which act cooperatively in order to expand collective intelligence in the country, shaping multi-institution training programs aimed at the health area.	ABRASCO, 2020 3rd dimension, two, 520.1
817	training and disclosure	training and training of human Resources	joint to promote training	feature	Promoting articulation with the Ministries of Science, Technology, Innovations and Communications and Education, with a view to including content related to the area of digital health in undergraduate and graduate courses in the health area.	PNIIS, 2021 cap iI, section V, art 8, III, 800.1
818	training and disclosure	training and training of human Resources	training in informatics in health	feature	Promotion of training, qualification, evaluation and permanent education of workers and health managers in the areas of information and health informatics, focusing on the person and their diversities, in particular, with regard to the collection and analysis of the item race, color and respect for the social name and gender identity	PNIIS, 2021 cap iI, section V, art 8, II, 799.1
819	training and disclosure	training and training of human Resources	health training digital	feature	Encouraging the development of specific programs for training in permanent education in the area of digital health.	PNIIS, 2021 chapter II, section V, art 8, IV, 801.1
820	training and disclosure	training and training of human Resources	health training digital	feature	Encouraging the inclusion of basic internship hours in undergraduate and graduate courses of continuing education activities developed in the public and private spheres on digital health and technologies for access to individual and collective health monitoring.	PNIIS, 2021 chapter II, section V, art 8, VII, 802.1
821	training and disclosure	training and training of human Resources	action plan	feature	5. Train health professionals in Health Informatics and ensure recognition of Health Informatics as a research area and Health Informatics as a profession.	ESD28, 2020 5, 720.1
822	training and disclosure	skills and training of team	training	feature	The healthcare organization ensures that opportunities to undertake appropriate training courses in providing telehealth services are made available	ISO13131, 2021 9.1.2, 88.1
823	training and disclosure	process	access to learning objects County	indicator	Description: overall number of accesses to learning objects by municipality; Numerator: global total of accesses to objects by municipality; Unit: hits/month; Source: data from tele-education offers; Note: Collection of Educational Resources at	MSNT5, 2014 annex I, frame 4, 408.1

#	theme	category	aspect	nature	element(s)	ref
					Saúde ARES is the UNASUS digital repository in which all content produced by the nuclei must be deposited, according to Technical Note 93/2013. Learning objects: text or audiovisual document available for access by professionals in a virtual environment Ex. Virtual Library, Cletâne Telessaúde at ARES/UNASUS.	
824	training and disclosure	process	global access to objects of learning	indicator	Description: global number of accesses to learning objects by state, municipality, team, points/month; Numerator: global total of accesses to objects; Unit: hits/month; Source: data from tele-education offers;<br><br>Note: Collection of Educational Resources in Health ARES is the UNASUS digital repository in which all content produced by the centers must be deposited, according to Technical Note 93/2013. Learning objects: text or audiovisual document available for access by professionals in a virtual environment Ex. Virtual Library, Cletâne Telessaúde at ARES/UNASUS.	MSNT5, 2014 annex I, frame 4,*407.1*
825	training and disclosure	process	global access to objects of learning by CBO	indicator	Description: global number of accesses to learning objects by CBO professional category; Numerator: global total of accesses to objects by CBO; Unit: hits/month; Source: data from tele-education offers;<br><br>Note: Collection of Educational Resources in Health ARES is the UNASUS digital repository in which all content produced by the centers must be deposited, according to Technical Note 93/2013. Learning objects: text or audiovisual document available for access by professionals in a virtual environment Ex. Virtual Library, Cletâne Telessaúde at ARES/UNASUS.	MSNT5, 2014 annex I, frame 4,*411.1*
826	training and disclosure	process	global access to objects of learningby team	indicator	Description: global number of accesses to learning objects per team; Numerator: global total of accesses to objects per team; Unit: hits/month; Source: data from tele-education offers;<br>Note: Collection of Educational Resources in Health ARES is the UNASUS digital repository in which all content produced by the centers must be deposited, according to Technical Note 93/2013. Learning objects: text or audiovisual document available for access by professionals in a virtual environment Ex. Virtual Library, Cletâne Telessaúde at ARES/UNASUS.	MSNT5, 2014 annex I, frame 4,*409.1*
827	training and disclosure	process	global access to objects of learningby point	indicator	Description: global number of accesses to learning objects by points; Numerator: global total of accesses to objects per point; Unit: hits/month; Source: data from tele-education offers;<br>Note: Collection of Educational Resources in Health ARES is the UNASUS digital repository in which all content produced by the centers must be deposited, according to Technical Note 93/2013. Learning objects: text or audiovisual document available for access by professionals in a virtual environment Ex. Virtual Library, Cletâne Telessaúde at ARES/UNASUS.	MSNT5, 2014 annex I, frame 4,*410.1*
828	training and disclosure	process	participation of telecast participants education by CBOpor team	indicator	Description: number of participants per professional category CBO per team; Numerator: total tele-education participants per CBO per team; Unit: participation/month; Source: data from tele-education offers;<br><br>Note: Tele-education activities: clinical meetings, courses, seminars, virtual library, etc. For recording purposes, synchronous and asynchronous activities must be monitored separately.	MSNT5, 2014 annex I, frame 4,*404.1*
829	training and disclosure	process	participation of telecast participants education by CBOpor state	indicator	Description: number of participants by CBO professional category by state; Numerator: total tele-education participants by CBO by state; Unit: participation/month; Source: data from tele-education offers;<br><br>Note: Tele-education activities: clinical meetings, courses, seminars, virtual library, etc. For recording purposes, synchronous and asynchronous activities must be monitored separately.	MSNT5, 2014 annex I, frame 4,*402.1*
830	training and disclosure	process	participation of telecast participants	indicator	Description: number of participants per CBO professional category per municipality; Numerator: total number of tele-education participants by CBO by municipality;	MSNT5, 2014 annex I, frame 4,*403.1*

#	theme	category	[illegible]	nature	[illegible] (s)participation/month; Source: ref offers data	
			[illegible]not by CBOpor County		tele-education;<br>Note: Tele-education activities: clinical meetings, courses, seminars, virtual library, etc. For recording purposes, synchronous and asynchronous activities must be monitored separately.	
831	training and disclosure	process	participation of telecast participants education by CBOpor point	indicator	Description: number of participants per CBO professional category per point/month; Numerator: total CBO tele-education participants per point/month; Unit: participation/ month; Source: data from tele-education offers;<br><br>Note: Tele-education activities: clinical meetings, courses, seminars, virtual library, etc. For recording purposes, synchronous and asynchronous activities must be monitored separately.	MSNT5, 2014 annex I, frame 4,*405.1*
832	training and disclosure	process	participation in phone activities team education	indicator	Description: number of activities performed by the team; Numerator: total tele-education activities carried out by team; Unit: activities/month; Source: data from tele-education offers;<br>Note: Tele-education activities: clinical meetings, courses, seminars, virtual library, etc. For recording purposes, synchronous and asynchronous activities must be monitored separately.	MSNT5, 2014 annex I, frame 4,*401.1*
833	training and disclosure	process	participation in phone activities education by state	indicator	Description: number of activities carried out by state; Numerator: total tele-education activities carried out by state; Unit: activities/month; Source: data from tele-education offers;<br>Note: Tele-education activities: clinical meetings, courses, seminars, virtual library, etc. For recording purposes, synchronous and asynchronous activities must be monitored separately.	MSNT5, 2014 annex I, frame 4,*398.1*
834	training and disclosure	process	participation in phone activities education by municipality	indicator	Description: number of activities carried out by municipality; Numerator: total tele-education activities carried out by municipality; Unit: activities/ month; Source: data from tele-education offers;<br><br>Note: Tele-education activities: clinical meetings, courses, seminars, virtual library, etc. For recording purposes, synchronous and asynchronous activities must be monitored separately.	MSNT5, 2014 annex I, frame 4,*399.1*
835	training and disclosure	process	participation in phone activities education per point	indicator	Description: number of activities performed per point; Numerator: total tele-education activities carried out per point; Unit: activities/month; Source: data from tele-education offers;<br>Note: Tele-education activities: clinical meetings, courses, seminars, virtual library, etc. For recording purposes, synchronous and asynchronous activities must be monitored separately.	MSNT5, 2014 annex I, frame 4,*400.1*
836	training and disclosure	process	production of learning objects	indicator	Description: number of learning objects available in ARES per month; Numerator: total CBO tele-education participants per point/month; Unit: objects/month; Source: data from tele-education offers;<br><br>Note: Collection of Educational Resources in Health ARES is the UNASUS digital repository in which all content produced by the centers must be deposited, according to Technical Note 93/2013. Learning objects: text or audiovisual document available for access by professionals in a virtual environment Ex. Virtual Library, Cletâne Telessaúde at ARES/UNASUS.	MSNT5, 2014 annex I, frame 4,*406.1*
837	training and disclosure	process	external promotion of telehealth	feature	There are communication mechanisms in the institution to inform and educate the population about the use of telehealth.	ARGMNMM, 2020 I.55,*597.1*
838	training and disclosure	process	internal promotion of telehealth		The institution has a strategy to promote feature for the health team the use of health services telehealth and remote consultations.	ARGMNMM, 2020 I.54,*596.1*
839	training and disclosure	process	use of tele education	indicator	Description: number of active points (at least one monthly service use) in tele-education; Numerator: total points participating in tele-education activities; Unit: points/ month; Source: request data; Note: 1 usage fee will be calculated from the information provided by the<br><br>Answered requests from active points and points registered as implanted; 2 Questions received and answered during tele-education activities should not be counted as teleconsulting. Note: Source: Telehealth Manual for Attention	MSNT5, 2014 annex I, frame 2,*390.1*

#	theme	category	aspect	nature	element(s)	ref
