# Supplementary material for: Diagnostic evaluation of institutions as a basis for designing the Brazilian maturity model of telehealth services
Source: BMC Health Serv Res. 2024 Mar 25;24:372. doi: 10.1186/s12913-024-10723-8 (PMC10964504; doi:10.1186/s12913-024-10723-8)
Supplement: Supplementary file 2 — Supplementary Material 2 [file 12913_2024_10723_MOESM2_ESM.pdf]

Supplementary data 2: Online survey applyed.

Data collection instrument for qualifying the telehealth center

starting block

1 respondent
Identification of the respondent(s).

Answer in text by topics:
a) As a respondent, please inform your full name. If there is more than one respondent associated with the same core, please leave each name on one line.
b) Inform preferred contact email. If there is more than one respondent please leave each email on a line.
c) As a respondent, please inform your preferred contact telephone number. If there is more than one respondent please leave each phone on a line.

two Core identification
Inform basic identification data of the telehealth center.

Answer in text by topics:
a) Full and official name of the telehealth center.
b) Acronym for identifying the telehealth center.
c) Popular name used to refer to the core.
d) Inform the date of creation of the nucleus.
e) Inform the core institution, if any.
f) Inform the complete address of the nucleus.
g) Inform the official e-mail of the nucleus.
h) Inform which web addresses (URLs) are formally used by the telehealth center.
i) Inform the addresses of the social media formally used by the nucleus.

3 Core coordination
Identification of members of the telehealth core coordination.

Answer in text by topics:
a) Full name of the person responsible for coordination.
b) Full name of the other people who act in the coordination,

indicating their functions.

4 Project identification with DESDMS
About the project associated with the Department of Digital Health of the Ministry of Health DESDMS

Answer in text by topics:
a) Title of the project/object with the DESDMS. If you have a current contract, please inform. If not, inform the last contract.
b) Indicate the interested party or the host institution of the telehealth center associated with this contract.
c) indicate the period of validity (month/year of beginning and end) of the contracts entered into with the DESDMS.
d) From the current contract with DESDMS, or related to the latter, inform the general objective of the contract/object.
e) Inform the specific objectives of the contract.

5 free item
Please feel free to use this item on the collection instrument to send comments, criticisms, suggestions or any other data you consider important in relation to the activities of the telehealth center.

Response in long text format. Supports sending a file in PDF format.

block 1 · structure and management

1 Services offered by the core
Indicate the status of each possible telehealth service listed below in terms of your offering in the telehealth hub. If necessary, feel free to submit comments below about any specificity or service not listed.

Reference:830,UN2030, 2016;348,MSPC5, 2017;302,308,HAOC3, 2019;770, 787,DESD, 2021;

Options:
1 Genetic counseling
2 Training - teleconsultant training 3
Communication - press support 4
Communication - news by email 5
Communication - website
6 Communication - social networks

7 Clinical guidelines - dissemination 8 Clinical guidelines - elaboration 9 Integration with networks and/or centers 10 Remote monitoring of patients 11 Ombudsman - own or from the host institution 12 Scientific and technological research

13 Telehealth point - advisory for implementation 14 Telehealth point - communication channel for doubts 15 Telehealth point - visitation for quality assurance 16 Referral protocol - disclosure

17 Referral protocol - preparation 18 Activity reports - public disclosure
19 Activity reports – for the exclusive use of the DESDMS 20 Second formative opinion - elaboration
21 Second formative opinion - publication, dissemination 22 User service - doubts, complaints, suggestions

23 Tele-education - class, course, short course 24 Tele-education - IES certificate 25 Tele-education - discussion forum
26 Tele-education - didactic-pedagogical material 27 Tele-education - lecture, dissemination, seminar 28 Teleconsultation
29 Asynchronous teleconsulting 30 Teleconsulting for ICU staff 31 Synchronous teleconsulting (by phone)
32 Synchronous teleconsulting (web conference with or without video)
33 Telediagnosis
34 Telediagnosis - national platform for telediagnostics 35 Telemonitoring
36 Teleguidance
37 Telerehabilitation
38 Teleregulation
39 Telescreening

Indicate for each option:
does not offer
under development
yes, in specific situations yes,
regularly

Response in long text format.

two Regulation and referrals
With regard to health promotion and prevention activities, respond to the items in the table indicating the related situation. If necessary, provide comments for clarification.
Reference:710,ESD28, 2020;

Options:
1 Does the center support health promotion and disease prevention activities?
2 Does the nucleus guarantee support to the Health Care Networks (referral and counter-referral)? Does the center integrate its telehealth services with the care flow?
3 Does the nucleus offer any support for epidemiological surveillance? 4 Does the core support continuity of care at all levels?
5 Does the center support care lines?

Indicate for each option:
does not offer
yes, but incipient, experimental or local at the host institution
yes, regionally
yes, nationally

Response in long text format.

3 organization chart and career
Regarding the organizational chart and career plan in digital health, respond by describing what is necessary:
Reference:587,ARGMNMM, 2020;453,BDHI, 2021;

Answer in text by topics:
a) Does the telehealth center have an organizational chart for the telehealth area?
b) Is the telehealth center included in the official organizational chart of the host institution?
c) Does the host institution have a specific professional career in the area of telehealth?
d) Are there roles and a career path for specific digital health professionals in the public and private sectors that are applicable to the core team?

4 Themes of the National Health Plan
Indicate from the list below the themes of the National Health Plan PNS2016 2019 for which the telehealth center offers service.

Services:
a) teleconsulting
b) teleconsultation
c) telediagnosis
d) tele-education

Options for each service (no/yes):
Outpatient care in the SUS Basic and specialized production Basic care

attention to emergencies
Pharmaceutical care Attention to
specific populations Hospital care in
the SUS
women's health
Health care for people with chronic diseases
Transplants
Immunizations
Mental health
Attention to the health of the indigenous population
Production complex and science, technology and innovation in health
Care for people with disabilities
child health
Other (please specify)

Response in long text format.

block 2 · financial and budget management

1 Financial plan
Does the host institution of the telehealth center implement a financial plan for telehealth/digital health services that considers the costs, benefits, accessibility and sustainability of telehealth services?

Reference:95,ISO13131, 2021;

Response in long text format. Supports sending a file in PDF format.

Complementary answer in scale:
0. Does not occur, does not apply, there is no information
1. Implement basic financial plan for core infrastructure.
2. Implements a plan for basic infrastructure and some services offered.
3. Implements a plan for all infrastructure and services offered.

two Core direct costs
Regarding the DIRECT costs of the telehealth center, indicate the approximate percentage of the typical financial impact that each item represents on the center's maintenance costs (totaling 100%. If there are others, please indicate in the text below.
Reference:320,HAOC5, 2020;

Options:
1 Permanent team (eg CLT wages and payments per work; 2 Fellow team: wages and payments per work;
3 Physical installations and infrastructure (property rent, depreciation rate, public services: energy, telecommunications and internet, water and sewage; cleaning material and snacks, office material and office supplies);

4 Other items related to service maintenance (management system software licenses, accounting service, employee and analyst training, taxes and mandatory licenses, permits, authorizations);

5 Foundation, autarky or similar services: taxes and administration fee;
6 Others (please specify below)

Indicate for each option:
approximate percentage

Response in long text format.

3 economic result
With regard to monitoring and evaluation by financial situation indicators, respond to the items. If there is any document specifying such analyses, please send it.
Reference:259,HAOC1, 2019;

Answer in text by topics:
a) Does the telehealth center monitor economic result indicators resulting from its activities? If yes, please comment specifying which indicators.

b) Has the center calculated the cost-minimization for its services (per capita cost/procedure/diagnosis with equal effectiveness)? Comment. c) Has the nucleus calculated the cost-utility for its services (impact on access or quality with the same similar cost)? Comment. d) What indicators seem to reflect the value resulting from the activities of the telehealth center? Please suggest up to 5 bookmarks, even if they are bookmarks that are not currently in use. It supports sending a file in PDF format.

block 3 · processes and activities

1 Flowchart and clinical protocols
Does the telehealth center establish flows, deadlines and protocols based on the best available scientific evidence? Please comment.
Reference:348,MSPC5, 2017;221,HAOC1, 2019;78,ISO13131, 2021;

Response in long text format.

Complementary answer in scale:
0. Does not occur, does not apply, there is no information
1. Establishes good practices and a culture of technical-scientific research to support the core's activities.
2. Establishes method based on the best scientific evidence to support the core activities.
3. Establish and fully document decisions based on the best scientific evidence.

two patient consent
Is there a procedure for obtaining and documenting patient consent before participating in a telehealth activity (in particular, teleconsultation and synchronous teleconsultation)? If yes, please specify for which services and which approach is implemented respectively. Indicate more than one alternative if it occurs for the same service. If the service is not offered, just leave it blank.

Reference:127,LGPD, 2018;229,HAOC1, 2019;646,ARGMNMM, 2020;4,ISO13131, 2021;

Services:
a) teleconsulting
b) teleconsultation
c) telediagnosis
d) tele-education

Options for each service (no/yes):
verbally with/without recording signed
paper document electronic registration
on the platform other method (specify)

Response in long text format.

3 failures and incidents
Regarding procedures to manage possible failures, answer.

Reference:167,PAHO, 2016;592,606,ARGMNMM, 2020;

Answer in text by topics:

a) Are there standardized procedures for communicating and documenting possible technical and technological failures that may affect the assistance activity during the provision of telehealth services (teleconsultation, teleconsultation, telediagnosis, etc.? Comment your answer. If yes, send a PDF document .
b) Is there a contingency plan for equipment and connectivity failures? Comment your answer. If so, send the PDF document.

c) What are the typical technical problems in the center's telehealth service? List and comment.
d) Typically, how long does it take on average to solve a technical problem in the center's telehealth service? What is the minimum and maximum time? It supports sending a file in PDF format.

block 4 · human resources

1 Core team composition
Describe the typical composition of the telehealth center team, indicating the number of professionals and their typical monthly workload. Consider typical values over the term of the contract.

Reference:375,MSNT5, 2014;758,MSNT50, 2015;204,PAHO, 2016;302, HAOC3, 2019;

Options:
1 general coordinator
2 Other coordinators, directors 3
Administrative assistant
4 Field Coordinator 5 Field
Monitor
6 Teleregulator (specify specialties) 7
Teleconsultant (specify specialties)
8 Computer professional, programmer, telecommunications, database

9 Health informatics professional, digital health, telehealth 10
Education/EaD professional
11 Technical-scientific researcher 12 Specialist in
AI, data analysis 13 Lawyer, professional in digital
law 14 Undergraduate and postgraduate students
15 Interns

16 Formal or informal external directors 17
Others (specify)

Indicate for each option:
number of professionals
typical monthly workload

Response in long text format.

two Core team training
Has the telehealth hub documented/listed competencies, experiences, knowledge and skills associated with each functional profile necessary for ICT professionals to be active participants in the functioning of the hub and the implemented digital health strategy?

Reference:723,ESD28, 2020;308,309,HAOC3, 2019;

Response in long text format. Supports sending a file in PDF format.

Complementary answer in scale:
0. Does not occur, does not apply, there is no information
1. Yes, informally with exchanges of news, information and materials.
2. Yes, with documentation of information/materials but with low impact on participation.
3. Yes, with documentation of information/materials and high impact on participation.

3 Core team qualification
Does the telehealth center have a telehealth team with clinical qualification in terms of experience and training, training in clinical practice in the specialty in practice? What is the procedure practiced by the nucleus to guarantee the adequate qualification of this team?

Reference:256,HAOC1, 2019;

Response in long text format. Supports sending a file in PDF format.

Complementary answer in scale:
0. Does not occur, does not apply, there is no information
1. The current clinical team is insufficient and/or has low qualifications for the services offered.
2. The current clinical team is sufficient and qualified for the services currently offered.
3. The current clinical team is sufficient and qualified for the services offered and expansion of activities.

block 5 · training and dissemination

1 Core team training; Training plan; Regarding the permanent education of your own team:
Reference:376,MSNT5, 2014;302,308,309,HAOC3, 2019;723,ESD28, 2020; 528,ABRASCO, 2020;642,ARGMNMM, 2020;449,BDHI, 2021;

Answer structured in subitems:
a) Does the telehealth center carry out continuing education activities? Please comment on typical periodicity.
b) The telehealth center encourages digital health - in its broadest sense - to be part of the continuing education curriculum of health workers who work in the center, including physicians, nurses, community health agents, dentists/ dentists, administrators , professionals in information and communication technology ICT, technical professionals and technologists?

c) Does the telehealth center carry out a training plan to achieve the proposed objectives that combines undergraduate, specialization, master's and doctoral efforts?
d) Does the telehealth center carry out technical training so that its team is qualified to use the telehealth tools related to the services offered? What is the typical periodicity? e) Do partner or external institutions participate in team training? To specify.

f) Does the telehealth center promote permanent education to facilitate the understanding of detailed protocols, such as, for example, teledermatology?

g) Does the telehealth center regularly promote and support the training of teleconsultants through periodic meetings and offering training activities?
h) Does the host institution establish some ongoing education process for the health team of the telehealth center regarding the use of mechanisms and structures to guarantee the authenticity of the records, the maintenance of these records in the chain of custody, and the security of these records in the health information systems?
i) Does the host institution establish a permanent education process for the core health team regarding the obligations arising from personal data protection laws, and which data are sensitive?

j) Does the host institution establish a permanent education process for the core health team regarding the national standards applicable to the teleconsultation modality?

Level response for each sub-item:
0. Does not occur, does not apply, there is no information
1. There are good practices and incentives for this, but informal and/or sporadic

2. There is a formal policy, but with partial impact and/or scope
3. There is a formal policy, with high impact and breadth

Response in long text format.

two Training of requesting professionals
Regarding the permanent education of requesting professionals, primary care teams and active teams:
Reference:376,MSNT5, 2014;348,MSPC5, 2017;308,309,HAOC3, 2019;723, ESD28, 2020;

Answer structured in subitems:
a) Does the telehealth center carry out training and qualification of requesting professionals, primary care teams or active teams in order to facilitate the use of services and the requests made?

b) Does the telehealth center identify the number of professionals to be trained by profile and size the demand for education and training? Answer below how often you do this and by what means you can obtain data to measure this demand.
c) Does the telehealth center systematically and formally disclose its flows, deadlines and protocols, based on the best available scientific evidence, to requesting professionals? Answer below how this is done and what are the main dissemination channels.

Level response for each sub-item:
0. Does not occur, does not apply, there is no information
1. There are good practices and incentives for this, but informal and/or sporadic
2. There is a formal policy, but with partial participation and/or scope
3. There is a formal policy, with high participation and breadth

Response in long text format.

block 6 · infrastructure and technology

1 Physical structure
What is the physical structure available for core activities?
Reference:256,HAOC1, 2019;1810,DESD, 2021;

Answer in text by topics:

a) In square meters:
b) How many work bays (or rooms, if applicable) are there for the secretariat:
c) How many work bays (or rooms, if applicable) are there for teleconsultants, mediators:
d) How many work bays (or rooms if applicable) are there for all coordination:
e) Number of rooms and meeting space capacity:
f) Number of rooms and space capacity for face-to-face consultations:
g) Other spaces available:
h) Where is the telehealth center located? Within the host institution or in another establishment? Please comment.

Complementary answer in scale:
0. Does not occur, does not apply, there is no information
1. The physical structure is minimal for the services currently offered
2. There is a satisfactory physical structure for the services offered at the moment
3. There is a satisfactory physical structure for the services offered and possible expansion of activities

two telehealth platform
Indicate the platforms that the telehealth center uses for its regular activities. Identify all situations that occur. Please inform the name of the platform, institution responsible or owner, and whether the use is free, paid or financially supported.
Reference:346,TICSAÚDE, 2021;

Answer in text by topics:
a) Platform(s) built locally
b) Platform(s) of other core(s)
c) Partner institution platform(s)
d) National platform(s)
e) Commercial platform(s)

Complementary answer in scale:
0. Does not occur, does not apply, there is no information
1. There is a platform, but incipient or under development
2. There is a platform, but it is unsatisfactory and/or specific to some services
3. There is a fully functioning and integrated platform

3 Technological structure
On the use of the SRES electronic health record system by the telehealth center:
Reference:610,ARGMNMM, 2020;

Answer structured in subitems:
a) Is there an SRES electronic health record or electronic patient medical history system at the host or partner institution?
b) Does this system integrate the patient's registration with the SUS?
c) Is this system integrated with the patient's clinical history in the SUS?

d) Is this system integrated with the patient's clinical history at the health provider at the host institution?

Level response for each sub-item:
0. Does not occur, does not apply, there is no information 1. Yes, but incipient or under development
2. Yes, but unsatisfactory and/or specific to some services
3. Yes, fully functioning and integrated

Response in long text format.

4 Technological structure
Does the telehealth center have technological storage capacity, with proper security, to document and record virtual meetings and other data from the services offered?
Reference:626,ARGMNMM, 2020;

Answer in text by topics:
a) Is the current capacity of storage space sufficient for the next 2 years?

b) Is it stored at the host institution, at a partner or in a cloud solution?
c) Is this storage capacity exclusive to core activities? Or is it shared with the host institution?

Complementary answer in scale:
0. Does not occur, does not apply, there is no information
1. The digital storage capacity is minimal and/or insufficient for the services offered.
2. There is sufficient digital storage capacity for the currently offered services.
3. There is digital storage capacity for the services offered and expansion of activities.

5 Technical support
Does the telehealth center have access to technical support for telehealth services, which includes the provision of specialized technical assistance? Identify the supports they have at their disposal by ticking the multiple options. Leave blank if you don't have specific support.
Reference:52,ISO13131, 2021;

Services:
a) teleconsulting
b) teleconsultation
c) telediagnosis
d) tele-education

Options for each service (no/yes):

informal support from individuals, collaborators
formal core support
formal support from the host institution
contracted or third-party support

Response in long text format.

block 7 · monitoring and evaluation

1 Accounting for activities
Of the services offered by the telehealth center, please indicate below which ones are periodically monitored and by which type of monitoring. If necessary, you can indicate more than one option, for example, in the case of using manual monitoring combined with automatic S1S2 methods.

Reference:302,HAOC3, 2019;648,ARGMNMM, 2020;103,783,ISO13131, 2021;

Services:
a) teleconsulting
b) teleconsultation
c) telediagnosis
d) tele-education

Options for each service (no/yes):
Service NOT monitored: N1. There is no follow-up agreed or performed

Service NOT monitored: N2. There is no associated indicator or method
Service NOT monitored: N3. Scheduled to be monitored Monitored service: S1. Manual, by observation, subjective
Monitored service: S2. automatic, systematic, objective
Monitored service: S3. In partnership with host institution and/or third party(s)
Monitored service: S4. Certification or accreditation process
Rated service: A1. Manual, by observation, subjective
Rated service: A2. automatic, systematic, objective
Rated service: A3. In partnership with host institution and/or third party(ies) Evaluated service: A4. Certification or accreditation process
Type of evidence: E1. Merging several trials (systematic review of trials)

Type of evidence: E2. Experiments – trials (clinical) Type of evidence: E3. Notes – cohort
Type of evidence: E4. Expert opinions, descriptive studies and baseline studies

Response in long text format.

two Accounting for activities
Indicate in the table by which categorizations the telehealth center performs the accounting of its activities.
Reference:379,380,383,386,387,391,392,393,394,395,MSNT5, 2014;308, HAOC3, 2019;210,PAHO, 2016;

Services:
a) teleconsulting
b) teleconsultation
c) telediagnosis
d) tele-education

Categorizations for each service: By telehealth point
per team
By professional (applicant/participant) By state
By municipality
By type of exam (when involved) By specialty
By synchronous vs asynchronous activities
% of activities involving regulatory protocols approved by the CIB

Scale response for each categorization: does not perform
yes, but in testing, development yes, in specific situations
yes, regularly

3 Satisfaction survey
Regarding the satisfaction survey carried out by the telehealth center:
Reference:413,MSNT5, 2014;494,NTSHUUFMA, 2019;

Answer in text by topics:
a) Does the telehealth center have any mechanism to record whether the service requester was satisfied with the response received immediately after completing an activity (or in a short period)?

b) Is there any satisfaction survey system that considers a longer period, not immediately after the completion of an activity? Which axes are evaluated? For example, twice a year, the core conducts a survey of all applicants in the last period, questioning their satisfaction, usefulness of the services and suggestions and

criticisms to improve the services offered.
c) What concept of “satisfaction” is applied in core assessments? What are the requester's response options, and which of these are considered “satisfied”?

Complementary answer in scale:
0. Does not occur, does not apply, there is no information
1. Satisfaction survey is carried out, but informal or sporadic
2. Satisfaction survey is carried out regularly in part of the services offered
3. Satisfaction survey is carried out, integrated into services and regularly audited

4 Execution cronogram
What are the main difficulties and/or barriers to carrying out the activities that subsidize the provision of services in the telehealth center? Reference:779 ,DESD, 2021;

Response in long text format.

block 8 · acceptability and suitability

1 Leadership engagement
With regard to the leaders of the telehealth center:
Reference:241,HAOC1, 2019;

Answer in text by topics:
a) Are they engaged with the core health professionals? If so, comment on how this engagement is.
b) Are they engaged with health professionals from telehealth points, primary care, etc.? If so, comment on how this engagement is.

c) Are they engaged with patients? If so, comment on how this engagement is.
d) Are they engaged with the leaders of the host institution and/or associated health provider (hospital)? If so, comment on how this engagement is.
e) Are they engaged with municipal, state and/or federal managers? If so, comment on how this engagement is.
f) Are they engaged with societies, associations and/or technical-scientific committees in the field of digital health? If so, comment on how this engagement is.

g) Are they engaged with regional or federal legislators with action in the area of telehealth? If so, comment on how this engagement is.

Complementary answer in scale:
0. Does not occur, does not apply, there is no information
1. There is engagement, but informal or individual
2. There is engagement, with core professionals and/or local groups
3. There is engagement, with professionals and patients

block 9 · protection and security

1 Consent
Does the telehealth center or the host institution notify the patient/citizen and collect the consent form to provide telehealth services in accordance with the legislation of the territory? Comment.
Reference:3,ISO13131, 2021;

Response in long text format.

Complementary answer in scale:
0. Does not occur, does not apply, there is no information 1. Yes, but rarely or upon request
2. Yes, regularly in part of the services and/or situations
3. Yes, regularly in most jobs and situations

two Eletronic security
Does the host institution guarantee to the telehealth center that processes are implemented to monitor the reliability of the representation, generation, collection, transmission, exchange and use of data by telehealth services? Comment your answer.
Reference:15,ISO13131, 2021;

Response in long text format.

Complementary answer in scale:
0. Does not occur, does not apply, there is no information
1. Yes, but transfer such responsibilities to the core
2. Yes, it partially offers such guarantees and processes
3. Yes, it guarantees and performs periodic monitoring and evaluation

block 10 · legal and ethical aspects

1 health information systems
Regarding the information systems that support core services:

Reference:123,151,LGPD, 2018;309,HAOC3, 2019;645,ARGMNMM, 2020;

Answer structured in subitems:
a) Does the telehealth center or the host institution implement strategies to know the correct geolocation of patients (country, state, municipality) who use telehealth services?
b) Are the systems used to process personal data at the core structured in such a way as to meet security requirements, good practice and governance standards and the general principles set out in the LGPD?

c) Are there procedures for discarding data from the electronic records of the telehealth center services? Comment your answer.
d) Does the telehealth center keep the data provided for in the legislation confidential?

Level response for each sub-item:
0. Does not occur, does not apply, there is no information
1. Incipient, insufficient and/or partial aspect
2. Sufficient, regular or full appearance
3. Aspect fully attended to

Response in long text format.

block 11 · network, innovation and research

1 Education and research
With regard to education and research activities at the telehealth center:
Reference:832,TICSAÚDE, 2021;802,PNIIS, 2021;

Answer in text by topics:
a) Does the nucleus formally have a teaching and research department or sector? Comment.

b) Does the host institution have teaching and research departments or sectors that have a formal link with the nucleus? Which?
c) Does the telehealth center offer a basic internship for undergraduate and graduate courses for permanent education developed in the public and private spheres on digital health and technologies for access to monitoring in individual and collective health? Comment.
d) Does the telehealth center have this stage recognized in the market?
e) Does the telehealth center or its host institution offer a certification recognized by HEIs?

Complementary answer in scale:
0. Does not occur, does not apply, there is no information
1. Education and research activities at the nucleus are incipient
2. Education and research activities at the nucleus are satisfactory and regular
3. Education and research activities at the core are full, recognized and integrated with partners

two Regulation; Innovation in technology;
Regarding the National Health Data Network RNDS
Reference:738,740,ESD28, 2020;

Answer structured in subitems:
a) Does the telehealth center participate in the discussion regarding the potential volume of data for the National Health Data Network RNDS with an inventory of beds related to Covid-19 and other infectious diseases?

b) Does the telehealth center or its host institution participate in the construction of the RNDS data lake (data lake) provided for in the Federal Government's Digital Health Strategy?

Level response for each sub-item:
0. Does not occur, does not apply, there is no information
1. Yes, but informally following news and information
2. Yes, participate formally and/or institutionally
3. Yes, it fully participates in decisions and implementations

Response in long text format.

3 health surveillance
Is the integration between the RNDS, Conecta SUS and the host institution in the procedures related to Covid-19 care being carried out or does it have the participation of the telehealth nucleus?
Reference:738,740,ESD28,;

Response in long text format.

Complementary answer in scale:
0. Does not occur, does not apply, there is no information
1. Incipient, insufficient and/or partial aspect
2. Sufficient, regular or full appearance
3. Aspect fully attended to

block 12 · citizenship and sustainable development

1 Sustainability
Regarding the sustainability of the telehealth center:
Reference:265,HAOC1, 2019;

Answer structured in subitems:
a) Is there any planning by the telehealth center or by its host institution to ensure the sustainability of the services offered? Comment.

Level response for each sub-item:
0. Does not occur, does not apply, there is no information
1. Yes, but core-restricted and/or limited, outdated
2. Yes, but core-restricted, updated and satisfying
3. Yes, there is planning in partnership with the host institution

Response in long text format. Supports sending a file in PDF format.

two Sustainability
Are there core funding sources other than the DESDMS? Please indicate in the respective field. Indicate approximately what is the relative percentage share of funding from each source (totaling 100%. Feel free to include comments.
Reference:308,309,HAOC3, 2019;

Options:
1 DESDMS
2 ministries/federal:
3 municipal health departments: 4
state health department: 5 municipal
consortiums:
6 intermunicipal consortia: 7
support foundations:
8 development agencies:
9 innovation and research agencies: 10
direct resources from the host institution:

11 core own resources: 12 others
(please specify):

Indicate for each option:
list of sources
approximate percentage

Response in long text format.

3 Citizenship
Indicate which actions to involve citizens are carried out by the telehealth center.
Reference:717,ESD28, 2020;

Answer structured in subitems:
a) The core has an instructional video channel on the use of applications for citizens and examples of real-life cases.
b) Conducts webinars in partnership with the National Health Council, with emphasis on forums for users and other representatives of civil society, to present social control with the benefits of using digital health solutions.

c) Develops and disseminates distance education courses free of charge for citizens and offered on the available Government digital platforms. d) Carries out or collaborates in advertising campaigns to disseminate digital health actions for citizens.
e) The core establishes a specific flow and service at the SUS Ombudsman for Digital Health services.

Level response for each sub-item:
0. Does not occur, does not apply, there is no information
1. Incipient, insufficient and/or partial aspect
2. Sufficient, regular or full appearance
3. Aspect fully attended to

Response in long text format.

© 2022Telehealth Expansionv4.0 · Aspects v1.08.830
