# Supplementary material for: Diagnostic evaluation of institutions as a basis for designing the Brazilian maturity model of telehealth services
Source: BMC Health Serv Res. 2024 Mar 25;24:372. doi: 10.1186/s12913-024-10723-8 (PMC10964504; doi:10.1186/s12913-024-10723-8)
Supplement: Supplementary file 3 — Supplementary Material 3 [file 12913_2024_10723_MOESM3_ESM.pdf]

Instructive for in-depth interviews

This instrument was conceived as a detailed step-by-step to help and align the application of in-depth interviews with the coordinators or focal points of the 19 centers participating in the project.

The objective of this action is to qualify the diagnosis of the nuclei, collecting information that was not completed in the questionnaire.

Composition of the interview groups:2 interviewers and 1 support. 1 to 2 interviewees maximum

Length of interviews:60 minutes, with possibility of extension to 90 minutes

Means of Interlocution:TEAMS Microsoft corporate software (It is recommended to download the application and conduct the interview on a computer)

Booking dates: Mondays, Tuesdays and Wednesdays from 4 pm to 7 pm.

Instructions:

- Interviewers and the technical support person must enter 30 minutes before the time communicated to the interviewee. The two interviewers must previously read the report on filling in the questions from the interviewed group.
- Interviewers must agree in advance who will conduct the interview. The three must have the telephone contact of the interviewee.
- The main interviewer should briefly introduce himself, introduce the second interviewer and the support person. The latter must enter the room with video and audio, greet the interviewee(s) and then turn off the video.
- The support will be responsible for reminding the interviewer to start, pause and end the recording. It should open a timer.
- Every 15 minutes, support must inform via chat or another agreed means how much is left to finish the interview.
- It is suggested that the interviewers have a pen and paper within reach to write down intercurrences.
- Support and second interviewer should pass their notes to the main interviewer.
- It will be the responsibility of the main interviewer to make a documentary account of the interview with the notes gathered.
- A staff member will transcribe the interviews.
- Each grouping of questions should take an average of 5 minutes.
- I suggest that there be a break every 20 minutes, but it is up to the interviewer according to the progress of the conversation.

- Everyone who is going to enter the meeting must have the answers sent by the interviewee open on the web or in PDF, and if possible keep the web page with the comparative (horizontal report),a very wide page, but that can support the conversation.

question script

The main interviewer must inform the interviewee that the interview will be recorded and transcribed. If he wants to say something that is not recorded, he can signal to pause the recording.

1. Please tell us your full name.<br>2. What is your profession?<br>3. How long have you been working with telehealth?

4. We found in your responses that your core [summarize what you read in the report]

5. What kind of telediagnostics does your center offer? Tell us a little about how this activity takes place. [block 1 – Structure and management]

6.In your experience as a manager, what challenges could you highlight for the development of the work? [block 1 – Structure and management]

7. How do you think the nucleus should be evaluated by funders? What kind of indicators do you think would be most suitable? Demographics, number of health establishments in the territory, number of health professionals per capita [Block 2 – Finance and Budget]

8. How is the patient's consent in procedures involving telehealth? Do you think it's the ideal way? [block 3 - processes and activities]

[BREAK]

9. Is there a policy for hiring people with disabilities and parity between male and female workers at the nucleus? If not, do you think there should be? [block 4 - human resources]

10. What is the ideal way for a telehealth center to communicate with the population in your opinion? Have you already hired services or is there someone on the core team who knows the language of Libras to deal with people with little or no hearing? [Block 5 - training and dissemination]

11. Is your telehealth system fully digital? Do you still use paper in any procedures? Do you use the E-SUS AB? Do you consider that your nucleus is already prepared to enter the National Health Data Network (RNDS)? [Block 6 - infrastructure and technology]

12. There is the saying that whoever does it must also evaluate himself. Do you think it would be interesting to create instances of telehealth evaluation in the SUS? [Block 7 - monitoring and evaluation]

[BREAK]

13. Do people in the community served know the nucleus? Do you think this approximation would be good? What are the potential core funders that could be the target of active funding, in your opinion? [Block 8 - acceptability and suitability]
14. Do you think there are specific security measures for telehealth activities? Why? [Block 9 - protection and security]
15. Is the group you work with aware of the LGPD (federal law 13,709/2018)? Have you had contact with the person in charge of the data? [Block 10 - legal and ethical aspects]
16. Do you have access to the literature with evidence on telehealth type*Update*or *cochrane*? Do you think it could help with your work? Do you go to congresses and scientific meetings to update yourself? What kind of courses do you think should take place for permanent in-service education? [Block 11 - network, innovation and research]
17. Have you heard of green telehealth (Richard Scott)? What do you think this expression would be? [Block 12 - citizenship and sustainable development]
18. Would you like to make any comments about the research carried out or any suggestion or proposal about the evaluation process, considering this maturity model?
