# Supplementary material for: Diagnostic evaluation of institutions as a basis for designing the Brazilian maturity model of telehealth services
Source: BMC Health Serv Res. 2024 Mar 25;24:372. doi: 10.1186/s12913-024-10723-8 (PMC10964504; doi:10.1186/s12913-024-10723-8)
Supplement: Supplementary file 4 — Supplementary Material 4 [file 12913_2024_10723_MOESM4_ESM.pdf]

Supplementary data 4: Informed consent to begin the online survey after login the website

Dear Telehealth Center Manager:

We are inviting you to participate in the survey by the Department of Digital Health of the Ministry of Health DESDMS within the scope of the project “Expansion and Standardization of Health Centers”. This study by the RNP National Teaching and Research Network is being carried out by consultants Dr. Angélica Baptista Silva Department of Human Rights, Health and Cultural Diversity DIHS/ENSP/FIOCRUZ and Dr. Ivan Torres Pisa Department of Health Informatics, Escola Paulista of Medicine DIS/EPM/UNIFESP.

Identification
Expansion and Standardization Project for Telehealth Centers
Department of Digital Health, Ministry of Health DESDMS National
Teaching and Research Network RNP
FioCuz FioTec Support Foundation)
Angélica Baptista Silva FioCruz), Ivan Torres Pisa UNIFESP
Contact: telessaude.painel@rnp.br
Web page:https://telessaude.fiocruz.br/ampliacao-telessaude
Step: Study on the digital health maturity of telehealth centers

goal
The objective of this research is to better understand the reality of SUS telehealth services provided in the territory. By completing the collection instrument, you will be collaborating to build a national model of digital health maturity in telehealth services and will effectively contribute to data collection for the construction of future public policies specific to telehealth and to support DESDMS actions.

Participants
Managers of nineteen telehealth centers were invited to participate in this survey by the DESDMS.
If you are the leader of a telehealth center, you can also participate. Contact us via email for more details.

Period
Completion of the collection instrument can be done within 10 days from the first access.

Expenses and Benefits
By participating in this research you will have no personal expenses at any stage of the study and there will also be no individual financial compensation.

Scratchs
Your participation is voluntary, which means you have the right to decide whether or not you want to participate. If you do not wish to participate, there will be no charge, that is, you will not be harmed in any way. There is no physical risk in participating in this study.
You can stop the search at any time. You may find that certain questions bother you because the information we collect is about experiences that reveal data from your work practice. So you can choose not to answer any questions that make you uncomfortable.

As for the social risks of participating in the study, special care will be taken to protect your privacy (see section below) so that your participation will only be known to you, the research team and those with whom you choose to share this information.
To minimize legal risks for participants, you will receive a password upon agreeing to this term. Data anonymization procedures are in accordance with Federal Law 13.709/2018, known as the General Law for the Protection of Personal Data LGPD.
If you have problems, concerns, doubts, or suggestions about the survey, contact the coordinators at telessaude.painel@rnp.br .

confidentiality
This is an identified survey and for telehealth managers. If you want to participate, you will need to provide your telephone number and email address, which will be archived with your survey responses.
The responses you provide will be used for DESDMS internal research and organizational purposes. Your contact information will be kept private and will not be shared with anyone outside the project, and will not be directly shared publicly with survey responses. All records of study and survey responses will be archived in secure databases. Your research information will be shared with researchers who are allocated at DESDMS, RNPRUTE, UNIFESP and Fiocruz.

When information about this study is presented at scientific or management meetings or in scientific journals, your identity will not be revealed without prior authorization.
Special care will be taken to protect your privacy in open responses so that other researchers can analyze your core data. Personal information will be removed or changed before study files are shared or results are published by placing the information in an accessible public file. However, you will be contacted later to make available information about your nucleus.

Costs to Participate
There is no cost to participate in this study.

Pay to Participate
There is no payment to participate in this research.

Withdrawing from the Study
You may choose to stop participating in this research at any time. To withdraw from the study, contact the research coordinators by email telessaude.painel@rnp.br.

Return of the Study
Feedback on this survey will be presented in online and face-to-face meetings. There will be publicity on social networks. You will be invited to participate.

Return of the Study
For questions about this study or if you have problems, concerns, questions, or suggestions about the research, contact the coordinators at telessaude.painel@rnp.br

We recommend that this term be printed immediately after signing.
